# Catalyst- and metal-free C(*sp*^2^)–H bond selenylation of (*N*-hetero)-arenes using diselenides and trichloroisocyanuric acid at room temperature

**DOI:** 10.1038/s41598-023-41430-9

**Published:** 2023-08-31

**Authors:** José S. S. Neto, Isis J. A. Granja, Marcos R. Scheide, Marcelo S. Franco, Cassio A. O. Moraes, Adilson Beatriz, Dênis P. de Lima, Giancarlo V. Botteselle, Tiago E. A. Frizon, Sumbal Saba, Jamal Rafique, Antonio L. Braga

**Affiliations:** 1 Departamento de Química, Universidade Federal de Santa Catarina–UFSC, Florianópolis, SC 88040-970 Brazil; 2 Instituto de Química, Universidade Federal de Goiás–UFG, Goiânia, GO 74690-900 Brazil; 3 Instituto de Química, Universidade Federal do Mato Grosso do Sul–UFMS, Campo Grande, MS 79074-460 Brazil; 4 Departamento de Química, Universidade Estadual do Centro-Oeste–UNICENTRO, Guarapuava, PR 85819-110 Brazil; 5 Universidade Federal de Santa Catarina–UFSC, Campus Araranguá, Araranguá, SC 88905-120 Brazil

**Keywords:** Synthetic chemistry methodology, Synthetic chemistry methodology, Green chemistry

## Abstract

In this paper, we report an eco-friendly approach for the C(*sp*^2^)–H bond selenylation of imidazopyridines and other *N*-heteroarenes as well as simple arenes at ambient temperature. This new protocol consists of the reaction between (*N*-hetero)-arenes and the diorganyl-diselenides and trichloroisocyanuric acid (TCCA)-ethanol reagent system. In a short reaction time, the desired selenylated products were obtained regioselectively in good yields, with tolerance for a wide range of functional groups.

## Introduction

The construction of the C–Se bond is gaining increasing interest in organic synthesis, as these compounds exhibit fascinating biological characteristics^1–4^. In this regard, diorganyl-selenides are well-known for their diverse biological properties, mainly their antioxidant, anti-inflammatory, anti-Alzheimer and anticancer activities^5–9^. These ubiquitous structures play a fundamental role in modern organic synthesis and are employed in several reactions as catalysts, ligands and synthetic intermediates in total synthesis, as well as in ionic liquids^10, 11^. They are also applied in materials science^12^. Therefore, research studies have led to important discoveries regarding selective C-Se bond formation and in this context, a notable approach is direct selenylation reactions^2, 3, 10, 13–17^. 

**Figure 1:**
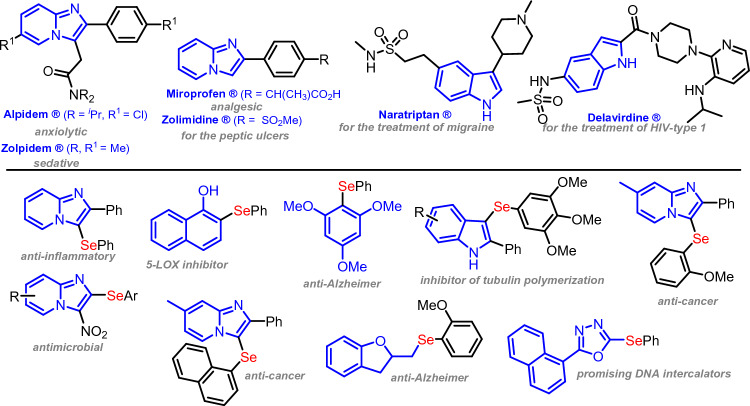
Biologically-relevant imidazo[1,2-*a*]pyridine (IP), indole and selenylated *N*-heteroarenes.

Similarly, *N*-heteroarenes, e.g., imidazo[1,2-*a*]pyridine (IP), imidazo[2,1-*b*]thiazole and indole are privileged scaffolds^18–20^, given their pharmaceutical, biological and materials science applications^18, 20–22^. These motifs are present in several commercially-available drugs (Fig. 1), highlighting the importance of these nuclei^19–21^. Therefore, they are considered as structures of interest in organic synthesis^23–28^.

Considering the therapeutic properties of (*N*-hetero)arenes and the biological relevance of organoselenium compounds, molecular hybridization of these structures could lead to molecules with promising biological properties (Fig. 1)^29–31^. In this regard, a new synthetic methodology to the construct the C–Se bond in (*N*-hetero)arenes has become a research hotspot^2–4, 36–43^.

In contrast to cross-coupling reactions, the formation of *N*-heteroarene containing organoselenides via C(*sp*^2^)−H bond functionalization provides a straightforward one-step bond formation route. This approach remains underexplored, although direct C–H functionalization is an atom-economical and greener alternative. For this type of direct C(*sp*^2^)−H bond selenylation of (*N*-hetero)arenes with diorganyl diselenides, there are two possible pathways: (a) nucleophilic species from arenes, generated in situ^44, 45^ and (b) electrophilic species from diselenides, generated in-situ^46, 47^. In terms of practicality, the former pathway is limited due to narrow substrate scope, while the development of a new method involving the activation of diselenides through the later pathway is highly desirable.

Although they offer good features, some of the previously used methods are associated with limitations in terms of applicability/sustainability, e.g., pre-functionalized coupling partners, non-green solvents, excess of organoselenium source, limited substrate scope, long reaction time and high temperature, low atom economy, transition metal catalyst, malodorous reagents, and multi-step processes.

On the other hand, trichloroisocyanuric acid (TCCA), a green chlorination agent^48^, is a stable and inexpensive reagent, commonly found in commercial products for swimming-pool disinfection^49^. Due to its highly electrophilic chlorine content and ease of handling, it is used as an efficient chlorine source in several reactions for the chlorination of organic compounds as well as in oxidation reactions^50^.

The development of a new alternative and benign method for the synthesis of organoselenyl containing IPs and other *N*-heteroarene hybrid structures with a broad scope, involving the use of a greener solvent at room temperature and ease of handling, which could provide high efficiency under neutral reaction conditions, would be highly desirable and advantageous.

**Figure 2:**
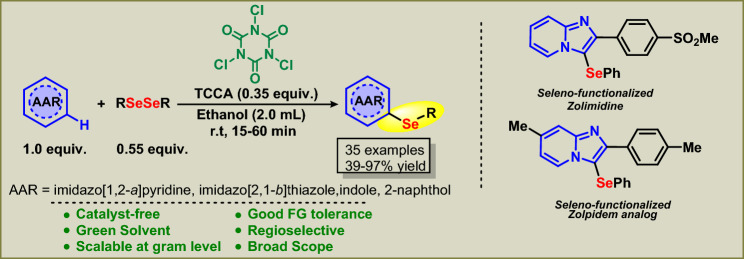
TCCA mediated C(*sp*^2^)–H bond selenylation of (*N*-hetero)-arenes using diselenides.

Continuing our research on direct organochalcogen functionalization and the development of eco-friendly processes^23, 27, 48, 51–53^, herein we describe, for the first time, the TCCA-mediated synthesis of biologically-relevant organoselenyl-indoles, -imidazoles and -arenes through C(*sp*^2^)–H bond selenylation, using diselenide. This new transition-metal free, alternative, and sustainable protocol offers ease of reagent handling and is operated in a short time at room temperature. It is applicable to a very broad scope of substrates, using EtOH as the solvent, and the procedure can be scaled up to the multi-gram scale (Fig. 2).

## Materials and methods

### General procedure for synthesis of selenylated-(*N*-hetero)-arenes by TCCA

In a Schlenk tube equipped with a stirring bar was charged with the TCCA (0.35 mol equiv.) and diselenide 2 (0.55 mol equiv.) in 1.0 mL of anhydrous ethanol and let to react for 5 min. After that, was added the respective (*N*-hetero)-arenes (0.25 mmol) and 1.0 mL of anhydrous ethanol. After the total consumption of starting materials, the reactional contend was diluted with 25.0 mL of ethyl acetate followed of extraction with distilled water (2 × 10 mL) and brine (1 × 10.0 mL). The organic phase was dried over MgSO_4_, filtered, and concentrated under reduced pressure. The residue was then subjected to purification on column chromatography of silica gel as stationary phase and eluate with appropriate solvent to afford the desired product.

### 2-phenyl-3-(phenylselanyl)imidazo[1,2-*a*]pyridine (**3a**)

Obtained as pale yellow solid (84.7 mg, 97%); Purified using hexane/ethyl acetate (80:20); mp:77–79 °C; ^1^H NMR (400 MHz, CDCl_3_) δ: 8.32 (d, *J* = 7.0 Hz, 1H), 8.18–8.12 (m, 2H), 7.71 (d, *J* = 9.0 Hz, 1H), 7.47–7.35 (m, 5H), 7.30–7.25 (m, 1H), 7.18–7.06 (m, 5H), 6.81 (t, *J* = 6.5 Hz, 1H). ^13^C NMR (100 MHz, CDCl_3_) δ: 151.8, 147.8, 133.8, 130.9, 129.7, 128.8, 128.5, 128.36, 128.3, 126.7, 126.5, 125.6, 117.5, 113.0, 102.9.

### 3-((4-chlorophenyl)selanyl)-2-phenylimidazo[1,2-*a*]pyridine (**3b**)

Obtained as yellow solid (78.8 mg, 82%); Purified using hexane/ethyl acetate (70:30); mp: 159–160 °C; ^1^H NMR (400 MHz, CDCl_3_) δ: 8.28 (d, *J* = 6.8 Hz, 1H), 8.17–8.09 (m, 2H), 7.71 (d, *J* = 9.0 Hz, 1H), 7.43 (t, *J* = 7.5 Hz, 2H), 7.37 (d, *J* = 7.0 Hz, 1H), 7.32–7.26 (m, 1H), 7.11 (d, *J* = 8.5 Hz, 2H), 7.00 (d, *J* = 8.5 Hz, 2H), 6.83 (t, *J* = 7.0 Hz, 1H). ^13^C NMR (100 MHz, CDCl_3_) δ: 151.96, 147.82, 133.62, 132.89, 129.81, 129.57, 129.13, 128.75, 128.62, 128.40, 126.67, 125.45, 117.64, 113.21, 102.48.

### 3-((4-fluorophenyl)selanyl)-2-phenylimidazo[1,2-*a*]pyridine (**3c**)

Obtained as yellow solid (66.2 mg, 72%); Purified using hexane/ethyl acetate (70:30); mp: 97–100 °C; ^1^H NMR (200 MHz, CDCl_3_) δ: 8.34 (d, *J* = 7.0 Hz, 1H), 8.14 (dd, *J* = 8.0, 1.5 Hz, 2H), 7.72 (d, *J* = 9.0 Hz, 1H), 7.52–7.22 (m, 5H), 7.16–7.03 (m, 2H), 6.93–6.80 (m, 3H). ^13^C NMR (50 MHz, Chloroform-*d*) δ: 151.66, 147.67, 133.62, 130.37 (d, *J* = 7.7 Hz), 128.73, 128.51, 128.31, 126.51, 125.41, 117.58, 116.84 (d, *J* = 22.0 Hz), 113.08.

### 2-phenyl-3-((3-(trifluoromethyl)phenyl)selanyl)imidazo[1,2-*a*]pyridine (**3d**)

Obtained as yellow solid (66.9 mg, 64%); Purified using hexane/ethyl acetate (70:30); mp: 99–101 °C;^1^H NMR (300 MHz, CDCl_3_) δ 8.28 (d, *J* = 6.9 Hz, 1H), 8.11 (d, *J* = 6.9 Hz, 2H), 7.71 (d, *J* = 9.0 Hz, 1H), 7.46–7.26 (m, 6H), 7.19 (t, *J* = 7.9 Hz, 1H), 7.07 (d, *J* = 8.0 Hz, 1H), 6.83 (t, *J* = 6.8 Hz, 1H). ^13^C NMR (50 MHz, CDCl_3_) δ: 152.25 (s), 147.86 (s), 133.34 (s), 132.27 (s), 131.04 (s), 129.96 (s), 128.64 (s), 128.30 (s), 126.75 (s), 125.74–124.63 (m), 124.81 (q, *J* = 3.5 Hz), 123.47 (q, *J* = 3.5 Hz), 117.60 (s), 113.27 (s), 101.73 (s).

### 2-phenyl-3-(*p*-tolylselanyl)imidazo[1,2-*a*]pyridine (**3e**)

Obtained as yellow solid (83.7 mg, 92%); Purified using hexane/ethyl acetate (70:30); mp: 110–113 °C; ^1^H NMR (300 MHz, CDCl_3_) δ 8.24 (d, *J* = 6.9 Hz, 1H), 8.08 (d, *J* = 8.3 Hz, 2H), 7.60 (d, *J* = 9.0 Hz, 1H), 7.39–7.25 (m, 3H), 7.21–7.14 (m, 1H), 6.97–6.83 (m, 4H), 6.72 (t, *J* = 6.8 Hz, 1H), 2.14 (s, 3H).^13^C NMR (75 MHz, CDCl_3_) δ 151.63, 147.71, 136.76, 133.92, 130.54, 128.86, 128.60, 128.47, 128.36, 127.03, 126.43, 125.70, 117.54, 113.00, 103.35, 21.00.

### 2-phenyl-3-(*o*-tolylselanyl)imidazo[1,2-*a*]pyridine (**3f**)

Obtained as yellow solid (81.0 mg, 89%); Purified using hexane/ethyl acetate (70:30); mp: 84–82 °C; ^1^H NMR (300 MHz, CDCl_3_) δ 8.12 (d, *J* = 6.9 Hz, 1H), 8.01 (d, *J* = 6.9 Hz, 2H), 7.61 (d, *J* = 9.0 Hz, 1H), 7.28 (dt, *J* = 14.9, 7.1 Hz, 3H), 7.19–7.10 (m, 1H), 7.04 (d, *J* = 7.8 Hz, 1H), 6.94 (t, *J* = 6.7 Hz, 1H), 6.82–6.62 (m, 2), 6.43 (d, *J* = 7.9 Hz, 1H), 2.34 (s, 3H). ^13^C NMR (75 MHz, CDCl_3_) δ 152.33, 147.98, 136.48, 133.81, 131.41, 130.68, 128.76, 128.46, 128.31, 127.17, 126.84, 126.45, 125.66, 117.52, 113.01, 101.83, 21.14.

### 3-((4-methoxyphenyl)selanyl)-2-phenylimidazo[1,2-*a*]pyridine (**3g**)

Obtained as yellow solid (91.1 mg, 96%); Purified using hexane/ethyl acetate (70:30); mp: 97–98 °C; ^1^H NMR (400 MHz, CDCl_3_) δ: 8.37 (d, *J* = 7.0 Hz, 1H), 8.24–8.12 (m, 2H), 7.69 (d, *J* = 9.0 Hz, 1H), 7.45 (t, *J* = 7.5 Hz, 2H), 7.38 (t, *J* = 7.5 Hz, 1H), 7.31–7.24 (m, 1H), 7.09 (d, *J* = 9.0 Hz, 2H), 6.86–6.80 (m, 1H), 6.71 (d, *J* = 9.0 Hz, 2H), 3.69 (s, 3H). ^13^C NMR (100 MHz, CDCl_3_) δ: 159.13, 151.19, 147.53, 133.91, 130.80, 128.90, 128.47, 128.37, 126.41, 125.66, 120.54, 117.52, 115.49, 112.99, 104.17, 55.35.

### 3-((2-methoxyphenyl)selanyl)-2-phenylimidazo[1,2-*a*]pyridine (**3h**)

Obtained as yellow solid (81.0 mg, 86%); Purified using hexane/ethyl acetate (70:30); mp: 154–155 °C; ^1^H NMR (400 MHz, CDCl_3_) δ: 8.31 (d, *J* = 7.0 Hz, 1H), 8.17–8.06 (m, 2H), 7.73 (d, *J* = 9.0 Hz, 1H), 7.44–7.27 (m, 4H), 7.17–7.10 (m, 1H), 6.89–6.76 (m, 2H), 6.72–6.59 (m, 1H), 6.42 (dd, *J* = 7.5, 1.5 Hz, 1H), 3.92 (s, 2H). ^13^C NMR (100 MHz, CDCl_3_) δ: 156.72, 152.51, 148.12, 133.93, 128.80, 128.43, 128.34, 127.54, 127.39, 126.45, 125.98, 122.11, 119.97, 117.53, 112.94, 110.72, 101.28, 55.95.; IR ν_max_: 3058, 2830, 1471, 1342, 1232, 1235, 752.; HRMS-ESI*: m*/*z* [M+H]^+^ calcd. for C_20_H_17_N_2_OSe 381.0501, found: 381.0505.

### 3-(naphthalen-1-ylselanyl)-2-phenylimidazo[1,2-*a*]pyridine (**3i**)

Obtained as yellow solid (63.0 mg, 64%); Purified using hexane/ethyl acetate (70:30); mp: 158–160 °C; ^1^H NMR (300 MHz, CDCl_3_) δ 8.25 (d, *J* = 6.9 Hz, 1H), 8.15 (dd, *J* = 15.4, 7.8 Hz, 3H), 7.83 (d, *J* = 7.9 Hz, 1H), 7.73 (d, *J* = 9.0 Hz, 1H), 7.64 (d, *J* = 8.1 Hz, 1H), 7.60–7.48 (m, 2H), 7.47–7.31 (m, 3H), 7.30–7.22 (m, 1H), 7.11 (t, *J* = 7.7 Hz, 1H), 6.87 (d, *J* = 7.3 Hz, 1H), 6.74 (t, *J* = 6.8 Hz, 1H). ^13^C NMR (75 MHz, CDCl_3_) δ 152.57, 148.12, 134.34, 133.84, 132.31, 129.27, 128.83, 128.59, 128.41, 127.25, 126.70, 126.55, 126.51, 126.34, 125.79, 125.63, 125.21, 117.61, 113.08, 101.67.

### 2-phenyl-3-(thiophen-2-ylselanyl)imidazo[1,2-*a*]pyridine (**3j**)

Obtained as yellow oil (80.0 mg, 90 %); Purified using hexane/ethyl acetate (70:30); ^1^H NMR (400 MHz, CDCl_3_) δ 8.56 (d, *J* = 7.0 Hz, 1H), 8.23–8.18 (m, 2H), 7.66 (d, *J* = 9.0 Hz, 1H), 7.50 (t, *J* = 7.5 Hz, 2H), 7.41 (t, *J* = 7.5 Hz, 1H), 7.31–7.26 (m, 1H), 7.26–7.23 (m, 1H), 7.13 (dd, *J* = 3.5, 1.0 Hz, 1H), 6.93–6.87 (m, 2H). ^13^C NMR (100 MHz, CDCl3) δ 150.88, 147.36, 133.94, 132.89, 129.87, 129.15, 129.12, 128.55, 128.40, 128.07, 126.45, 125.62, 124.44, 117.68, 113.05.

### 3-(butylselanyl)-2-phenylimidazo[1,2-*a*]pyridine (**3k**)

Obtained as yellow oil (79.1 mg, 96%); Purified using hexane/ethyl acetate (90:10); ^1^H NMR (400 MHz, CDCl_3_) δ: 8.54 (d, *J* = 7.0 Hz, 1H), 8.23 (d, *J* = 7.0 Hz, 2H), 7.68 (d, *J* = 9.0 Hz, 1H), 7.47 (t, *J* = 7.5 Hz, 3H), 7.37 (t, *J* = 7.5 Hz, 1H), 7.31–7.22 (m, 1H), 6.90 (t, *J* = 7.0 Hz, 1H), 2.66 (t, *J* = 7.5 Hz, 2H), 1.46 (dt, *J* = 15.0, 7.0 Hz, 3H), 1.28 (dq, *J* = 14.5, 7.5 Hz, 4H), 0.75 (t, *J* = 7.5 Hz, 3H).; ^13^C NMR (100 MHz, CDCl_3_) δ 150.12, 147.12, 134.10, 128.83, 128.27, 128.23, 126.00, 125.68, 117.42, 112.78, 104.46, 32.16, 29.29, 22.73, 13.48; IR ν_max_: 3065, 2958, 2929, 1463, 1343, 755, 694; HRMS-ESI*: m*/*z* [M+H]^+^ calcd. for C_17_H_19_N_2_OSe 331.0709, found: 331.0705.

### 2-(4-methoxyphenyl)-3-(phenylselanyl)imidazo[1,2-*a*]pyridine (**4a**)

Obtained as yellow solid (65.7 mg, 70 %); Purified using hexane/ethyl acetate (80:20); mp: 93–95 °C; ^1^H NMR (400 MHz, CDCl_3_) δ: 8.31 (d, *J* = 7.0 Hz, 1H), 8.12 (d, *J* = 9.0 Hz, 2H), 7.69 (d, *J* = 9.0 Hz, 1H), 7.27 (ddd, *J* = 9.0, 7.0, 1.0 Hz, 1H), 7.18–7.12 (m, 3H), 7.12–7.06 (m, 2H), 6.96 (d, *J* = 9.0 Hz, 2H), 6.85–6.75 (m, 1H), 3.82 (s, 3H).; ^13^C NMR (100 MHz, CDCl_3_) δ: 159.99, 151.72, 147.74, 131.09, 130.06, 129.73, 128.22, 126.69, 126.41, 125.58, 117.33, 113.83, 112.89, 102.07, 55.34.

### 2-(3-methoxyphenyl)-3-(phenylselanyl)imidazo[1,2-*a*]pyridine (**4b**)

Obtained as White solid (96.7 mg, 95%); Purified using hexane/ethyl acetate (70:30); mp: 103–105 °C; ^1^H NMR (400 MHz, CDCl_3_) δ: 8.35–8.30 (m, 1H), 7.81–7.66 (m, 3H), 7.33 (t, *J* = 8.0 Hz, 1H), 7.29–7.22 (m, 1H), 7.16–7.05 (m, 5H), 6.91 (dd, *J* = 8.0, 2.6 Hz, 1H), 6.83–6.74 (m, 1H), 3.77 (s, 3H). ^13^C NMR (100 MHz, CDCl_3_) δ: 159.52, 151.56, 147.63, 135.09, 130.96, 129.67, 129.30, 128.23, 126.68, 126.46, 125.58, 121.22, 117.50, 114.88, 113.60, 113.02, 103.05, 55.23; IR ν_max_: 3035, 2835, 1476, 1344, 1215, 1051, 734, 687, 459; HRMS-ESI*: m*/*z* [M+H]^+^ calcd. for C_20_H_17_N_2_OSe 381.0501, found: 381.0506.

### 2-(3,4-dimethoxyphenyl)-3-(phenylselanyl)imidazo[1,2-*a*]pyridine (**4c**)

Obtained as a yellow oil (73.8 mg, 72%); Purified using ethyl acetate/hexane (1:1); ^1^H NMR (200 MHz, CDCl_3_) δ: 8.37 (d, *J* = 7.0 Hz, 1H), 7.84–7.65 (m, 3H), 7.35–7.07 (m, 6H), 6.93 (d, J = 8.0 Hz, 1H), 6.84 (t, *J* = 7.0 Hz, 1H), 3.91 (s, 3H), 3.85 (s, 3H). ^13^C NMR (50 MHz, CDCl_3_) δ: 151.60, 149.45, 148.76, 147.69, 131.20, 129.75, 128.15, 126.73, 126.55, 125.57, 121.42, 117.32, 113.02, 111.95, 110.98, 102.27, 55.94, 55.85; IR ν_max_ : 3380, 2935, 1707, 1479, 1340, 891, 812, 577, 461; HRMS-ESI*: m*/*z* [M+H]^+^ calcd. for C_21_H_19_N_2_O_2_Se 411.0612, found 411.0629.

### 2-(4-bromophenyl)-3-(phenylselanyl)imidazo[1,2-*a*]pyridine (**4d**)

Obtained as Off White solid (78.6 mg, 74%); Purified using hexane/ethyl acetate (80:20); mp: 135–137 °C; ^1^H NMR (400 MHz, CDCl_3_) δ: 8.38–8.29 (m, 1H), 8.06 (d, *J* = 8.5 Hz, 2H), 7.70 (d, *J* = 9.0 Hz, 1H), 7.59–7.50 (m, 2H), 7.30 (ddd, *J* = 9.0, 7.0, 1.0 Hz, 1H), 7.16 (dd, *J* = 5.0, 1.5 Hz, 3H), 7.07 (dd, *J* = 6.5, 3.5 Hz, 1H), 6.85 (td, *J* = 7.0, 1.0 Hz, 1H). ^13^C NMR (100 MHz, CDCl_3_) δ: 150.59, 147.82, 132.81, 131.56, 130.69, 130.32, 129.85, 128.35, 126.94, 126.80, 125.71, 122.91, 117.62, 113.28, 103.16.

### 2-(4-(methylsulfonyl)phenyl)-3-(phenylselanyl)imidazo[1,2-*a*]pyridine (**4e**)

Obtained as pale yellow solid (104.8 mg, 94%); Purified using ethyl acetate; mp: 142–148 °C; ^1^H NMR (400 MHz, CDCl_3_) δ: 8.42 (d, *J* = 8.5 Hz, 2H), 8.38 (d, *J* = 7.0 Hz, 1H), 7.99 (d, *J* = 8.5 Hz, 2H), 7.72 (d, *J* = 9.0 Hz, 1H), 7.35 (ddd, *J* = 9.0, 7.0, 1.0 Hz, 1H), 7.18 (dd, *J* = 5.0, 1.5 Hz, 3H), 7.08 (dd, *J* = 6.5, 3.0 Hz, 2H), 6.90 (td, *J* = 7.0, 1.0 Hz, 1H), 3.07 (s, 3H).; ^13^C NMR (100 MHz, CDCl_3_) δ: 149.19, 147.84, 139.80, 139.27, 130.24, 129.85, 129.34, 128.35, 127.32, 127.13, 127.07, 125.71, 117.77, 113.61, 104.39, 44.55; IR ν_max_: 3071, 2921, 1573, 1301, 1159, 1142, 773, 544, 530; HRMS-ESI*: m*/*z* [M+H]^+^ calcd. for C_20_H_17_N_2_O_2_SSe 429.0171, found: 429.0176.

### 2-(naphthalen-2-yl)-3-(phenylselanyl)imidazo[1,2-*a*]pyridine (**4f**)

Obtained as white solid (45.1 mg, 45%); Purified using hexane/ethyl acetate (80:20–70:30); mp: 140–141 °C; ^1^H NMR (200 MHz, CDCl_3_) δ: 8.66 (s, 1H), 8.42–8.29 (m, 2H), 7.92–7.81 (m, 3H), 7.76 (d, *J* = 9.0 Hz, 1H), 6.90–6.81 (m, 1H). ^13^C NMR (50 MHz, CDCl_3_) δ: 151.72, 147.93, 133.45, 131.32, 131.04, 129.83, 128.76, 128.61, 128.33, 127.93, 127.74, 126.92, 126.68, 126.51, 126.43, 126.17, 125.75, 117.65, 113.18, 103.55.

### 2-(5-chlorothiophen-2-yl)-3-(phenylselanyl)imidazo[1,2*-a*]pyridine (**4g**)

Obtained as white solid (86.8 mg, 89%); Purified using hexane/ethyl acetate (80:20–70:30); mp: 134–135 °C; ^1^H NMR (200 MHz, CDCl_3_) δ: 8.28 (d, *J* = 6.5 Hz, 1H), 7.76 (d, *J* = 4.0 Hz, 1H), 7.63 (d, *J* = 9.0 Hz, 1H), 7.31–7.13 (m, 6H), 6.89 (d, *J* = 4.0 Hz, 1H), 6.81 (t, *J* = 7.0 Hz, 1H); ^13^C NMR (50 MHz, CDCl_3_) δ: 147.64, 146.10, 135.42, 131.42, 130.21, 129.79, 128.78, 127.12, 126.93, 126.79, 125.94, 125.40, 117.25, 113.24, 102.23.

### 3-(phenylselanyl)imidazo[1,2-*a*]pyridine (**4h**)

Obtained as yellow solid (19.5 mg, 29%); Purified using hexane/ethyl acetate (75:25); mp: 60–61 °C; ^1^H NMR (400 MHz, CDCl_3_) δ: 8.27 (d, *J* = 7.0 Hz, 1H), 7.98 (s, 1H), 7.70 (d, *J* = 9.0 Hz, 1H), 7.31–7.26 (m, 1H), 7.17 (tt, *J* = 5.5, 2.5 Hz, 5H), 6.89–6.83 (m, 1H).; ^13^C NMR (100 MHz, CDCl_3_) δ: 148.39, 143.12, 130.66, 129.63, 129.12, 126.98, 125.88, 125.35, 118.04, 113.18, 106.61.

### 7-methyl-2-phenyl-3-(phenylselanyl)imidazo[1,2-*a*]pyridine (**4i**)

Obtained as white off solid (84.6 mg, 93%); Purified using hexane/ethyl acetate (8:2); mp: 154–157 °C; ^1^H NMR (200 MHz, CDCl_3_) δ: 8.23–8.08 (m, 3H), 7.50–7.33 (m, 4H), 7.19–7.04 (m, 5H), 6.70–6.60 (m, 1H), 2.41 (s, 3H). ^13^C NMR (50 MHz, CDCl3) δ: 151.71, 148.15, 137.79, 133.93, 131.24, 129.72, 128.78, 128.42, 128.34, 128.23, 126.67, 124.81, 116.06, 115.72, 102.11, 21.41.

### 6-methyl-2-phenyl-3-(phenylselanyl)imidazo[1,2-*a*]pyridine (**4j**)

Obtained as a white solid (73.7 mg, 81%); Purified using hexane/ethyl acetate (8:2); mp:148–149 °C; ^1^H NMR (200 MHz, CDCl_3_) δ: 8.29–8.01 (m, 3H), 7.62 (d, *J* = 9.0 Hz, 1H), 7.48–7.31 (m, 3H), 7.20–7.01 (m, 6H), 2.28 (s, 3H). ^13^C NMR (50 MHz, CDCl_3_) δ: 151.66, 146.83, 133.94, 131.31, 129.71, 128.73, 128.31, 128.12, 126.62, 123.35, 122.87, 116.90, 102.41, 18.42.

### 8-methyl-2-phenyl-3-(phenylselanyl)imidazo[1,2-*a*]pyridine (**4k**)

Obtained as Off White solid (81.5 mg, 90 %); Purified using hexane/ethyl acetate (85:15); mp: 130–132 °C; ^1^H NMR (400 MHz, CDCl_3_) δ: 8.18 (d, *J* = 7.0 Hz, 1H), 8.15–8.11 (m, 2H), 7.42 (t, *J* = 7.5 Hz, 2H), 7.37–7.32 (m, 1H), 7.15–6.99 (m, 6H), 6.71 (t, *J* = 7.0 Hz, 1H), 2.70 (s, 3H). ^13^C NMR (100 MHz, CDCl_3_) δ: 151.54, 148.12, 134.19, 131.26, 129.68, 128.97, 128.34, 128.29, 127.63, 126.63, 125.22, 123.49, 112.97, 103.20, 16.95.

### 6-chloro-2-phenyl-3-(phenylselanyl)imidazo[1,2-*a*]pyridine (**4l**)

Obtained as yellow solid (42.0 mg, 44%); Purified using hexane/ethyl acetate (75:25); mp: 146–148 °C; ^1^H NMR (400 MHz, CDCl_3_) δ: 8.40 (s, 1H), 8.17–8.11 (m, 2H), 7.66 (d, *J* = 9.5 Hz, 1H), 7.44 (t, *J* = 7.5 Hz, 2H), 7.38 (d, *J* = 7.0 Hz, 1H), 7.26 (d, *J* = 8.0 Hz, 1H), 7.22–7.16 (m, 3H), 7.13–7.07 (m, 2H). ^13^C NMR (100 MHz, CDCl_3_) δ: 152.53, 146.09, 133.36, 130.47, 129.92, 128.83, 128.77, 128.50, 128.49, 127.99, 127.08, 123.68, 121.56, 117.98, 103.86.; IR ν_max_: 3067, 1574, 1439, 1315, 1077, 804, 747, 696, 456.; HRMS-ESI*: m*/*z* [M+H]^+^ calcd. for C_19_H_14_ClN_2_OSe 385.0003, found: 385.0004.

### 2-(4-methoxyphenyl)-7-methyl-3-(phenylselanyl)imidazo[1,2-*a]*pyridine (**4m**)

Obtained as yellow solid (77.7 mg, 78 %); Purified using hexane/ ethyl acetate (70:30); mp: 100–101 °C; ^1^H NMR (400 MHz, CDCl_3_) δ 8.16 (d, *J* = 7.0 Hz, 1H), 8.10 (d, *J* = 9.0 Hz, 2H), 7.44 (s, 1H), 7.13 (d, *J* = 6.5 Hz, 3H), 7.07 (dd, *J* = 7.5, 2.0 Hz, 2H), 6.95 (d, *J* = 9.0 Hz, 2H), 6.62 (d, *J* = 7.0 Hz, 1H), 3.80 (s, 3H), 2.39 (s, 3H).; ^13^C NMR (100 MHz, CDCl_3_) δ: 159.87, 151.46, 148.00, 137.60, 131.33, 129.94, 129.64, 128.10, 126.56, 126.48, 124.67, 115.80, 115.46, 113.75, 101.17, 55.28, 21.34.

### 7-chloro-2-(4-chlorophenyl)-3-(phenylselanyl)imidazo[1,2-*a*]pyridine (**4n**)

Obtained as yellow solid (83.4 mg, 80 %); Purified using hexane/ethyl acetate (70:30); mp: 146–148 °C; 1H NMR (400 MHz, CDCl_3_) δ 8.40 (d, J = 1.5 Hz, 1H), 8.10 (d, *J* = 8.5 Hz, 2H), 7.69 (d, *J* = 9.5 Hz, 1H), 7.44–7.33 (m, 2H), 7.29 (dd, *J* = 9.5, 2.0 Hz, 1H), 7.19 (dd, *J* = 6.5, 3.5 Hz, 3H), 7.10 (dt, *J* = 6.0, 3.0 Hz, 2H). ^13^C NMR (50 MHz, CDCl_3_) δ 150.77, 145.73, 134.85, 131.40, 129.86, 129.83, 128.60, 128.39, 127.14, 123.54, 121.81, 117.76, 77.00. IR ν_max_: 3047, 2362, 1738, 1452, 1323, 1073, 851, 732.; HRMS-ESI*: m*/*z* [M+H]^+^ calcd. for C_19_H_13_Cl_2_N_2_Se 418.9621, found: 418.9630.

### 4-(3-(phenylselanyl)imidazo[1,2-*a*]pyridin-2-yl)benzonitrile (**4p**)

Obtained as yellow solid (72.2 mg, 77 %); Purified using hexane/ethyl acetate (85:15); mp: 97–99 °C; ^1^H NMR (300 MHz, CDCl_3_) δ 8.35–8.24 (m, 3H), 7.72–7.59 (m, 3H), 7.32–7.26 (m, 1H), 7.07–6.99 (m, 3H), 6.84 (t, *J* = 7.4 Hz, 2H) 6.87–6.79 (m, 1H). δ ^13^C NMR (75 MHz, CDCl_3_) δ 149.08, 147.76, 138.26, 131.99, 130.14, 129.81, 128.95, 128.25, 127.07, 127.02, 125.62, 118.92, 117.70, 113.54, 111.57, 104.17.

### 7-methyl-3-(phenylselanyl)-2-(*p*-tolyl)imidazo[1,2-*a*]pyridine (**4o**)

Obtained as white solid (83.1 mg, 88%); Purified using hexane/ethyl acetate (80:20–70:30); mp: 109–110 °C; ^1^H NMR (200 MHz, CDCl_3_) δ: 8.13 (s, 1H), 8.02 (d, *J* = 8.0 Hz, 2H), 7.61 (d, *J* = 9.0 Hz, 1H), 7.26–7.09 (m, 8H), 2.37 (s, 3H), 2.30 (s, 3H); ^13^C NMR (50 MHz, CDCl_3_) δ: 151.83, 146.83, 138.24, 131.45, 131.13, 129.70, 129.55, 129.08, 128.61, 128.13, 126.58, 123.35, 122.74, 116.83, 102.09, 21.39, 18.42. IR ν_max_: 2919, 2356, 1534, 1332, 969, 687.; HRMS-ESI*: m*/*z* [M+H]^+^ calcd. for C_21_H_19_N_2_Se 379.0713, found: 379.0703.

### 2-phenyl-3-(phenylselanyl)imidazo[1,2-*a*]pyrimidine (**6a**)

Obtained as yellow solid (70.1 mg, 81%); Purified using hexane/ethyl acetate (1:1); mp: 98–99 °C; ^1^H NMR (400 MHz, CDCl_3_) δ: 8.64–8.57 (m, 2H), 8.31–8.27 (m, 2H), 7.48–7.38 (m, 3H), 7.20–7.17 (m, 3H), 7.11 (dd, *J* = 6.5, 3.0 Hz, 2H), 6.92 (dd, *J* = 7.0, 4.0 Hz, 1H). ^13^C NMR (100 MHz, CDCl_3_) δ: 153.07, 151.49, 150.81, 133.21, 130.05, 129.93, 129.06, 128.99, 128.55, 128.44, 127.15, 109.45, 101.73.

### 2-methyl-6-phenyl-5-(phenylselanyl)imidazo[2,1-*b*]thiazole (**6b**)

Obtained as White solid (92.8 mg, 83%); Purified using hexane/ethyl acetate (95:5); mp: 138–139 °C; ^1^H NMR (400 MHz, CDCl_3_) δ 8.08–8.04 (m, 2H), 7.38 (t, *J* = 7.5 Hz, 2H), 7.29 (t, *J* = 7.5 Hz, 1H), 7.20–7.14 (m, 6H), 2.35 (d, *J* = 1.5 Hz, 3H). ^13^C NMR (100 MHz, CDCl_3_) δ: 151.74, 151.02, 134.06, 131.78, 129.70, 128.40, 128.33, 127.88, 127.69, 126.69, 115.37, 102.03, 14.21.

### 6-(4-methoxyphenyl)-2-methyl-5-(phenylselanyl)imidazo[2,1-*b*]thiazole (**6c**)

Obtained as Yellow solid (105.3 mg, 97 %); Purified using hexane/ethyl acetate (9:1); mp: 88–89 °C; ^1^H NMR (400 MHz, CDCl_3_) δ: 7.99 (d, *J* = 9.0 Hz, 2H), 7.23–7.11 (m, 6H), 6.92 (d, *J* = 9.0 Hz, 2H), 3.81 (s, 3H), 2.37 (s, 2H).; ^13^C NMR (100 MHz, CDCl_3_) δ: 159.49, 151.79, 150.93, 131.98, 129.71, 128.98, 128.32, 126.78, 126.64, 126.38, 115.41, 113.79, 101.10, 55.36, 14.22.; IR ν_max_: 3107, 2831, 1608, 14668, 1246, 1031, 836, 727, 686, 453; HRMS-ESI*: m*/*z* [M+H]^+^ calcd. for C_19_H_17_N_2_OSSe 401.0221, found: 401.0218.

### 6-(4-fluorophenyl)-2-methyl-5-(phenylselanyl)imidazo[2,1-*b*]thiazole (**6d**)

Obtained as White solid (94.4 mg, 97 %); Purified using hexane/ethyl acetate (9:1); mp: 159–162 °C; ^1^H NMR (400 MHz CDCl_3_) δ: 8.06–8.01 (m, 2H), 7.21–7.14 (m, 6H), 7.06 (t, *J* = 8.5 Hz, 2H), 2.39–2.33 (m, 3H).; ^13^C NMR (100 MHz, CDCl_3_) δ: 162.65 (d, *J*_C-F_ = 247.16 Hz), 150.97 (d, *J*_C-F_ = 15.81), 131.63, 130.26 (d, *J*_C-F_ = 3.27 Hz), 129.77, 129.43 (d, *J*_*C-F*_ = 8.0 Hz), 128.38, 126.81, 126.80, 115.37, 115.36, 115.14, 101.82, 14.22.; IR ν_max_: 3056, 2909, 1527, 1465, 1218. 840, 728, 555, 408.; HRMS-ESI*: m*/*z* [M+H]^+^ calcd. for C_18_H_14_FN_2_SSe 389.0021, found: 389.0023.

### 2-phenyl-3-(phenylselanyl)-1*H-*indole (**6e**)

Obtained as Brown Oil (33.9 mg, 39%);Purified using hexane/ethyl acetare (95:5); ^1^H NMR (400 MHz, CDCl_3_) δ: 8.59 (s, 1H), 7.73 (dd, *J* = 8.0, 1.5 Hz, 2H), 7.65 (d, *J* = 8.0 Hz, 1H), 7.46–7.37 (m, 4H), 7.29–7.17 (m, 4H), 7.15–7.07 (m, 3H).; ^13^C NMR (100 MHz, CDCl_3_) δ: 142.21, 136.30, 132.22, 132.17, 131.66, 129.18, 128.77, 128.65, 128.43, 127.39, 125.55, 123.42, 121.25, 121.07, 111.12.

### 1-(phenylselanyl)naphthalen-2-ol (**6f**)

Obtained as Orange solid (44.8 mg, 60%); Purified using hexane/ethyl acetate (95:5); mp: 75–77 °C; ^1^H NMR (400 MHz, CDCl_3_) δ 8.27 (d, *J* = 8.5 Hz, 1H), 7.87 (d, *J* = 9.0 Hz, 1H), 7.78 (d, *J* = 8.0 Hz, 1H), 7.51–7.45 (m, 1H), 7.40–7.31 (m, 2H), 7.12 (s, 6H).; ^13^C NMR (100 MHz, CDCl_3_) δ: 156.34, 135.92, 132.90, 130.66, 129.56, 129.19, 128.59, 128.03, 127.04, 126.71, 123.89, 116.68, 109.11.

### 1-(*p*-tolylselanyl)naphthalen-2-ol (**6g**)

Obtained as Brown solid (41.3 mg, 53%); Purified using hexane/ethyl acetate (95:5); mp: 57–58 °C; ^1^H NMR (400 MHz, CDCl_3_) δ: 8.28 (d, *J* = 8.5 Hz, 1H), 7.86 (d, *J* = 9.0 Hz, 1H), 7.78 (d, *J* = 8.0 Hz, 1H), 7.52–7.44 (m, 1H), 7.39–7.31 (m, 2H), 7.16 (s, 1H), 7.07 (d, *J* = 8.0 Hz, 2H), 6.94 (d, *J* = 8.0 Hz, 2H), 2.22 (s, 3H).; ^13^C NMR (100 MHz, CDCl_3_) δ: 156.20, 136.73, 135.90, 132.73, 130.36, 129.52, 128.57, 127.96, 127.08, 126.82, 123.82, 116.65, 109.63, 21.07.

### 1-((4-chlorophenyl)selanyl)naphthalen-2-ol (**6h**)

Obtained as Brown solid (36.4 mg, 50%); Purified using hexane/ethyl acetate (95:5); mp: 108–109 °C; ^1^H NMR (400 MHz, CDCl_3_) δ: 8.21 (d, *J* = 8.5 Hz, 1H), 7.88 (d, *J* = 9.0 Hz, 1H), 7.79 (d, *J* = 8.0 Hz, 1H), 7.53–7.43 (m, 1H), 7.36 (td, *J* = 9.0, 8.5, 3.0 Hz, 2H), 7.12–7.02 (m, 5H). ^13^C NMR (100 MHz, CDCl_3_) δ: 156.34, 135.68, 133.17, 132.88, 130.48, 129.66, 129.60, 128.86, 128.69, 128.20, 126.78, 124.02, 116.74, 108.82.

## Results and discussion

The optimization of the reaction conditions was conducted using IP **1a** and diphenyl diselenide **2a** as model substrates, in the presence of TCCA at room temperature. Screening under various conditions was carried out (Table 1). Initially, the reactions were performed for a duration of 1 h. Considering that TCCA has 3 chlorine atoms, we focused on the stoichiometric quantity of TCCA (entries 1–5). On using 0.6 molar equiv. of TCCA, the selenylated product **3a** was obtained in 74% isolated yield (entry 1). There was an improvement in the yield of the reaction when we further decreased the quantity of TCCA (entry 2–3). The yield of **3a** remained constant when 0.35 molar equiv. of TCCA was used (entry 4), while a further decrease in the quantity of TCCA had a negative impact on the reaction (entry 5).

**Table 1:** Optimisation of reaction conditions.


Entry	**2a** (equiv)	Solvent (mL)	Time (min)	TCCA (equiv)	Yield (%)^a^
1	1.0	EtOH (5 mL)	60	0.6	74
2	1.0	EtOH (5 mL)	60	0.5	85
3	1.0	EtOH (5 mL)	60	0.4	95
4	1.0	EtOH (5 mL)	60	0.35	96
5	1.0	EtOH (5 mL)	60	0.25	70
6	0.75	EtOH (5 mL)	60	0.35	96
7	0.55	EtOH (5 mL)	60	0.35	97
8	0.45	EtOH (5 mL)	60	0.35	78
9	0.55	CH_2_Cl_2_ (5 mL)	60	0.35	54
10	0.55	THF (5 mL)	60	0.35	45
11	0.55	EtOAc (5 mL)	60	0.35	60
12	0.55	Ethylene glycol (5 mL)	60	0.35	89
13	0.55	Glycerol (5 mL)	60	0.35	20
14	0.55	H_2_O (5 mL)	60	0.35	traces
15	0.55	MeOH (5 mL)	60	0.35	70
16	0.55	EtOH/H_2_O 50% (5 mL)	60	0.35	5
17	0.55	EtOH (5 mL)	60	0.35	90^a^
15	0.55	EtOH(5 mL)	60	0.35	81^b^
16	0.55	EtOH (5 mL)	60	0.35	94^c^
17	0.55	EtOH (2 mL)	60	0.35	96
18	0.55	EtOH (1 mL)	60	0.35	90
19	0.55	EtOH (0.5 mL)	60	0.35	84
20	0.55	EtOH (2 mL)	45	0.35	96
21	0.55	EtOH (2 mL)	30	0.35	97
22	0.55	EtOH (2 mL)	15	0.35	97
23	0.55	EtOH (2 mL)	10	0.35	54

Conditions: **1a** 0.25 mmol, r.t., room temperature, solvent (5 mL) for 1 h. ^a^Reaction at 50 °C; ^b^reaction with reflux; ^c^inert atmosphere.

After determining the appropriate quantity of TCCA, in the next step the stoichiometric quantity of diselenide **2a** was screened for this transformation (entries 5–8) and the ideal quantity of 0.55 molar equiv. of **2a** was obtained (entry 7). With regard to the influence of the solvent on the selenylation of **1a** (Table 1, entries 9–16), EtOH was found to be the most effective solvent. Increasing the reaction temperature to 50 °C (entry 17) or applying reflux (entry 18) had a negative impact on the yield of **3a**. Subsequently, when the reaction was performed under an inert atmosphere, no influence on the yield of selenylated product was observed (entry 7 vs. 16).

To minimize the quantity of solvent used, we then screened the effect of the solvent quantity, 1 mL (entries 7, 17–18) and 2 mL of EtOH (entries 7, 17–18) resulted in the best yield of **3a** (entry 17). Lastly, the effect of the reaction time was screened for this transformation (entries 7, 20–23). On decreasing the reaction time from 60 to 15 min, the desired product **3a** was isolated in almost constant yields. However, with a further decrease in the reaction time from 15 to 10 min, a significant decrease in the yield of **3a** was observed (entry 22 vs. 23).

**Figure 3:**
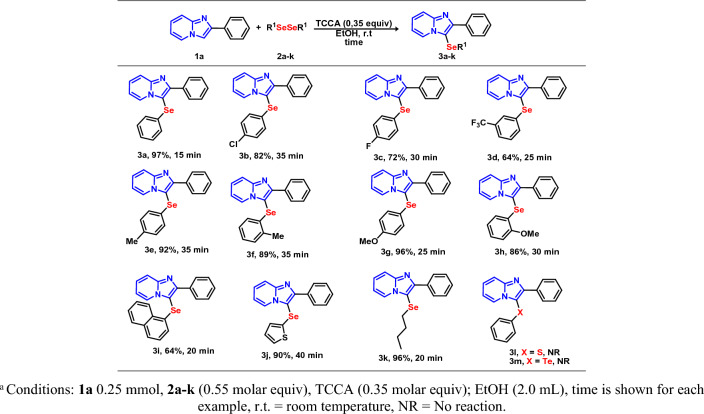
Scope of diorganyl diselenides **3**. Conditions: **1a** 0.25 mmol, **2a**–**k** (0.55 molar equiv), TCCA (0.35 molar equiv); EtOH (2.0 mL), time is shown for each example, r.t., room temperature; NR, No reaction.

**Figure 4:**
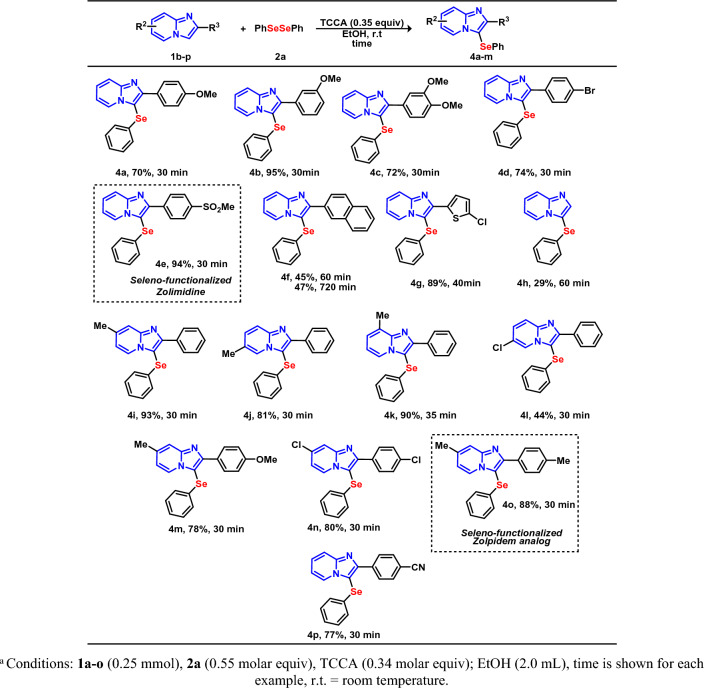
Scope of IP **1**. Conditions: **1a**–**o** (0.25 mmol), **2a** (0.55 molar equiv), TCCA (0.34 molar equiv); EtOH (2.0 mL), time is shown for each example, r.t., room temperature.

After ascertaining the best reaction parameters (Table 1, entry 22), the generality and scope of the C(*sp*^2^)–H bond selenylation of various other diorganyl diselenides **2** (Fig. 3) and IPs **1** (Fig. 4) were investigated. We first evaluated the efficiency of different diorganyl diselenides **2** while keeping IP **1a** constant (Fig. 3).

The reaction worked well for several diorganyl diselenides containing both electron-donating (EDG) (Me, OMe) and electron-withdrawing (EWG) (F, Cl, CF_3_) groups as well as bulky groups, verifying the tolerance and broad scope regarding the electronic and steric effects of several different substituents. All of the desired selenylated products were obtained in good to excellent yields. In general, EWGs at the phenyl ring afforded the respective product in a slightly lower yield as compare to the EDGs (**3b**–**d** vs. **3e,g**). These results revealed a small dependence on the electronic effect of the substituents bonded to the aromatic ring at the selenium atom. A greater stability of the electrophilic species generated can explain the higher yields when substituted rings with electronic density donor groups were used. In order to investigate the effect of steric hindrance, *ortho*-substituted aryl substrates were used and a weaker influence on the yields was observed as compared to the respective *para* derivatives (**3e,g** vs. **3f,h** respectively). Sterically-bulkier substrates (2-napthyl) resulted in the desired product **3i** in 64% yield. It should be noted that the reaction demonstrated a great tolerance to heteroaromatic diselenide and C-2 heteroaryl diselenide afforded the desired product **3j** with 90% yield.

Considering the importance of aliphatic selenides, the protocol was then extended to butylated organoselenides, since they play an important role in cross-coupling reactions^54^. Gratifyingly, diselenides with *n*-butyl groups directly bonded to the selenium atom, producing the corresponding products **3k** in excellent yield. Lastly, when diphenyl disulfide and ditelluride were tested as substrates under the optimized reaction conditions, no reaction was observed. Similarly, when thiophenol was used as the source of chalcogen, the expected product was not observed.

To further broaden the scope in relation to the substrate, the influence of the IP **1** moiety was evaluated with **2a** (Fig. 4), under the optimized reaction conditions. The IP nucleus was tested with different functionalities, e.g., Cl, Br, Me, MeO, attached at the aryl moiety as well as the heteroaryl substituent. It should be noted that the compounds **1b**–**p** are well tolerated in this transformation and resulted in the respective products **4a**–**o** with up to 95% yield. Electronics effects of the substituents attached to the aryl moiety at position C-2 demonstrate great tolerance for the selenylation reaction. OMe (EDG) at the *para*, *meta* and *para* and *meta* positions, for example, can be tolerated with short reaction times and satisfactory yields. In the case of an EWG at the aromatic ring, attached to the C-2, elevated yields were obtained for products **4d**–**e** and **4p**. Selenylated products with the bromo-substituent attached are important as they can be post-functionalized in other organic transformations. Moderate yields were obtained in the synthesis of **4f** and **4h**. In the case of **4f**, we postulate that steric hindrance by the naphthyl ring contributed to the decrease in the yield. Even when a longer reaction time was applied (720 min), there was no increase in the yield of the respective product. Notably, the product containing the heteroaromatic group at the C-2 position (**4g**) was also synthesized in high yield (88%), demonstrating the versatility of our protocol.

In the next step, the effect due to a variation in the functionalization of positions 6, 7 and 8 of IP was tested. The reaction tolerated the electronic effect and resulted in the selenylated product **4i**–**l** in 74-93% yields. In addition, on exploring the double electronic effects of the substituent on IP, the products **4m** and **4n** were also achieved in good yields. Encouraged by the results obtained from the selenylation of IP, we applied this transformation to the IP **1o**, which is the core for a commercially available drug with the trade name “Zolpidem”, affording the corresponding selenylated product **4o** with high efficiency and high yield. Similarly, it is also noteworthy to mention that by using Zolmidine (commercial drug, used in the treatment of peptic ulcer and gastro-oesophageal reflux disease) as substrate, afforded the desired selenylated product **4e** in 94% yield. These products are extremely relevant since it contains the same molecular scaffolds as Zolpidem and Zolmidine, which could have importance regarding the pharmacophoric characteristics of a possible newly discovered drug.

**Figure 5:**
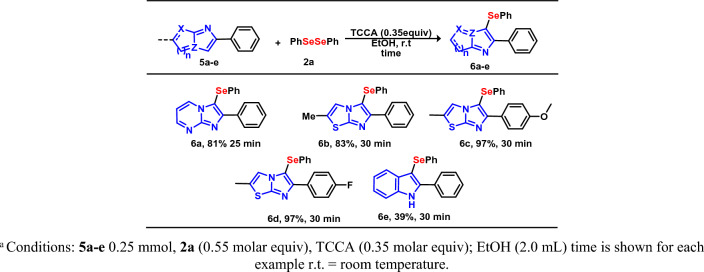
Synthesis of selenylated *N*-aromatic products **6a**–**e**. Conditions: **5a**–**e** 0.25 mmol, **2a** (0.55 molar equiv), TCCA (0.35 molar equiv); EtOH (2.0 mL) time is shown for each example r.t., room temperature.

Following the success in the TCCA-mediated C(*sp*^2^)–H bond selenylation of the IP indole **2**, this method was extended to structurally diverse *N*-heteroarenes **5a**–**e**, using diselenide **2a** under ideal reaction conditions (Fig. 5). It was observed that 2-phenylimidazo[1,2-*a*]pyrimidine **5a** furnished the corresponding C-3 selenylated product in 81% yield. When imidazo[2,1-*b*]thiazoles **5b**–**d** were tested as substrates, the respective products **6b**–**d** were achieved in very good to excellent yields. Furthermore, to extend the scope of the work to other heteroarenes, we tested indole **5c** as the substrate for selenylation, resulting in **6c** in 39% isolated yields. These findings demonstrate the potential application of this methodology to a diversity of heteroaromatics.

**Figure 6:**
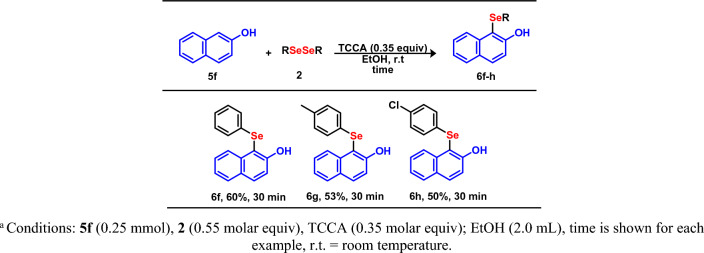
Scope of selenylated naphthol **6f**–**h**. Conditions: **5f** (0.25 mmol), **2** (0.55 molar equiv), TCCA (0.35 molar equiv); EtOH (2.0 mL), time is shown for each example, r.t., room temperature.

Following the success in the TCCA-mediated C(*sp*^2^)–H bond selenylation of *N*-heteroarenes, this method was extended to 2-naphthol **5f** and diorganyl diselenides **2** as the coupling partner (Fig. 6). To our delight, the reaction furnished the corresponding selenylated products **6f**–**h** in moderate to good yields, highlighting the potentially broad scope of this methodology.

**Figure 7:**
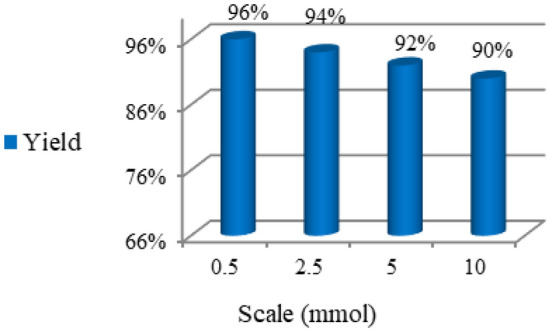
Results for the reaction at different scales.

To demonstrate the potential and the synthetic utility of our methodology, a series of reactions was performed at different scales in a normal laboratory set-up (Fig. 7; up to 10 mmol). For this, IP **1a** and diselenide **2a** were selected as substrates and were tested under optimized conditions, affording **3a** with no major decrease in yield. Thus, this protocol represents a practical synthetic method for the synthesis of biologically-relevant lead compounds on a larger scale.

**Figure 8:**
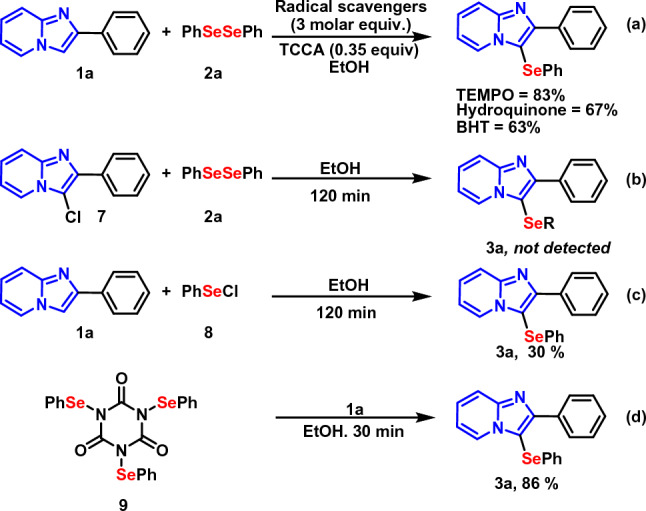
Investigation of the reaction mechanism.

Lastly, in order to gain further insights regarding the reaction and to tentatively propose a mechanism, some control experiments were conducted (Fig. 8). Firstly, the standard reaction was conducted in the presence of 3.0 molar equiv. of radical inhibitor (TEMPO, hydroquinone, BHT). It was found that radical scavengers did not hamper the reaction (Fig. 8a), excluding the possibility of a radical pathway. In the next step, some reactions were carried out in order to ascertain if a chlorinated species is involved in the reaction. In this experiment, 3-chloro-2-phenylimidazo[1,2-*a*]pyridine **7** was used as the substrate together with **2a**, without the presence of TCCA (Fig. 8b). In this case, no reaction was observed, eliminating the possibility of **7** as the intermediate.

Subsequently, when **1a** was treated PhSeCl **8**, under the optimized reaction conditions, the desired product was obtained in 30% isolated yield (Fig. 8c). In case of reaction with the selenium derivative of isocyanuric acid **9**, the selenylated product **3a** was obtained in 86% yield (Fig. 8d). This indicates that the electrophilic selenium species **9** could be involved in the reaction. These results, clearly highlights the active involvement of **9** as an intermediate.

**Figure 9:**
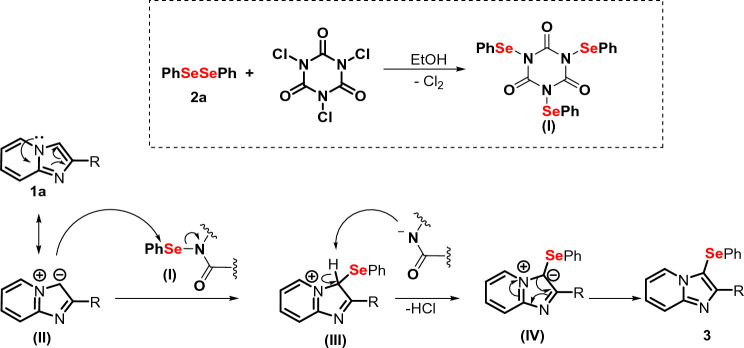
Proposed mechanism for the reaction.

Based on the results obtained from the control experiments and reported in the literature^55^, a possible mechanism was proposed using **1a** and **2a** as examples (Fig. 9). In the first step the electrophilic species **I** is most likely formed from the reaction of diselenide **2a** and TCCA. Subsequently, species **I** could react with IP **1a** via a canonical structure **II** at the C-3 position, generating the species **III**. Ethanol, used as a solvent, could play an important role in the reaction, by stabilizing species **II**. The selenylated species **III** would undergo deprotonation and restoration of the aromaticity, resulting in the desired product **3a**.

## Conclusions

In conclusion, we have developed a robust and straightforward method for the preparation of selenylated-imidazopyridines from the corresponding imidazopyridines and diorganyl diselenides in a very short reaction time. Under the optimized reaction conditions, which involve the use of a trichloroisocyanuric acid (TCCA)-ethanol system, this eco-friendly approach afforded the desired products in good yields. The reaction demonstrated tolerance for the electronic and steric effects of substituents, without the need for the exclusion of air and moisture. Moreover, this method could be applied to other *N*-heteroarenes as substrates. This is an important contribution considering the potential therapeutic application of these hybrid compounds.

The important features of this benign protocol are: (1) open to the air atmosphere; (2) very short reaction time; (3) inexpensive reagents; (4) gram-scalable; (5) green oxidant (6) greener solvent; and (7) applicability to structurally diverse *N*-heteroarenes.

### Supplementary Information

Supplementary Information.

## Data Availability

All data generated or analyzed during this study are included in this published article and its supplementary information file.
