# Supplementary material for: Catalyst- and metal-free C(sp2)–H bond selenylation of (N-hetero)-arenes using diselenides and trichloroisocyanuric acid at room temperature
Source: Sci Rep. 2023 Aug 31;13:14251. doi: 10.1038/s41598-023-41430-9 (PMC10471583; doi:10.1038/s41598-023-41430-9)
Supplement: Supplementary file 1 — Supplementary Information. [file 41598_2023_41430_MOESM1_ESM.pdf]

Supplementary Information

Catalyst- and metal-free C(sp^2)-H bond selenylation of (*N*-hetero)-arenes using diselenides and trichloroisocyanuric acid at room temperature.

José S. S. Neto[1], Isis J. A. Granja[2], Marcos R. Scheide[1], Marcelo S. Franco[1], Cassio A. O. Moraes[3], Adilson Beatriz [3], Dênis P. de Lima[3], Giancarlo V. Botteselle[4], Tiago E. A. Frizon[5], Sumbal Saba[2], Jamal Rafique[2,3*], and Antonio L. Braga[1,*]

[1] *Departamento de Química, Universidade Federal de Santa Catarina - UFSC, Florianópolis, 88040-970, SC-Brazil. http://labselen.ufsc.br; E-mail: braga.antonio@ufsc.br*

[2] *Instituto de Química, Universidade Federal de Goiás - UFG, Goiânia, 74690-900, GO-Brazil*

[3]*Instituto de Química, Universidade Federal do Mato Grosso do Sul - UFMS, Campo Grande, 79074-460, MS-Brazil. https://sintmol.ufms.br/; E-mail: jamal.rafique@ufms.br, jamal.chm@gmail.com*

[4] *Departamento de Química, Universidade Estadual do Centro-Oeste - UNICENTRO, 85819110, Guarapuava, PR - Brazil*

[5] *Universidade Federal de Santa Catarina – UFSC, Campus Araranguá, 88905120 Araranguá, SC-Brazil*

*****Corresponding author: jamal.chm@gmail.com (J.R.), braga.antonio@ufsc.br (A.L.B.)

TABLE OF CONTENTS

General Remarks S2
Synthesis of Starting Materials S2
Characterization Data Products S3
^{1}H and ^{13}C NMR Spectra S4
References S37

General Remarks

Starting materials obtained from commercial suppliers were used unless otherwise stated. Column chromatography was performed using silica gel 60 (diameter 0.05 - 0.10 mm) Macherey-Nagel. Thin layer chromatography (TLC) was performed using Macherey-Nagel pre-coated TLC sheets ALUGRAM® Xtra SIL with layer of 0.20 mm. Visualization was achieved by UV fluorescence, iodine chamber and acidic vanillin.

Synthesis of Starting Materials

The substrates imidazo[1,2-*a*]pyridines, imidazo[1,2-a]pyrimidines and imidazo[2,1-*b*]thiazoles were prepared according to the literature reports.[1-12]

Characterization Data Products

General considerations: The melting points were taken on a MQAPF-301 melting point apparatus, uncorrected. ^{1}H and ^{13}C NMR spectra were recorded on Varian NMR AS 400 spectrometer and Brucker NMR AC 200, with the samples dissolved in $CDCl_3$. Chemical shifts are informed in ppm downfield from the signal of TMS, used as internal standard, and the coupling constants (*J*) are expressed in Hertz (Hz). High-resolution mass spectral data were obtained on a Bruker microTOF-Q IIT instrument and Xevo G2-S QTOF (Waters) on ESI^+ mode. Infrared spectra were recorded on Bruker Alpha using KBr pellets.

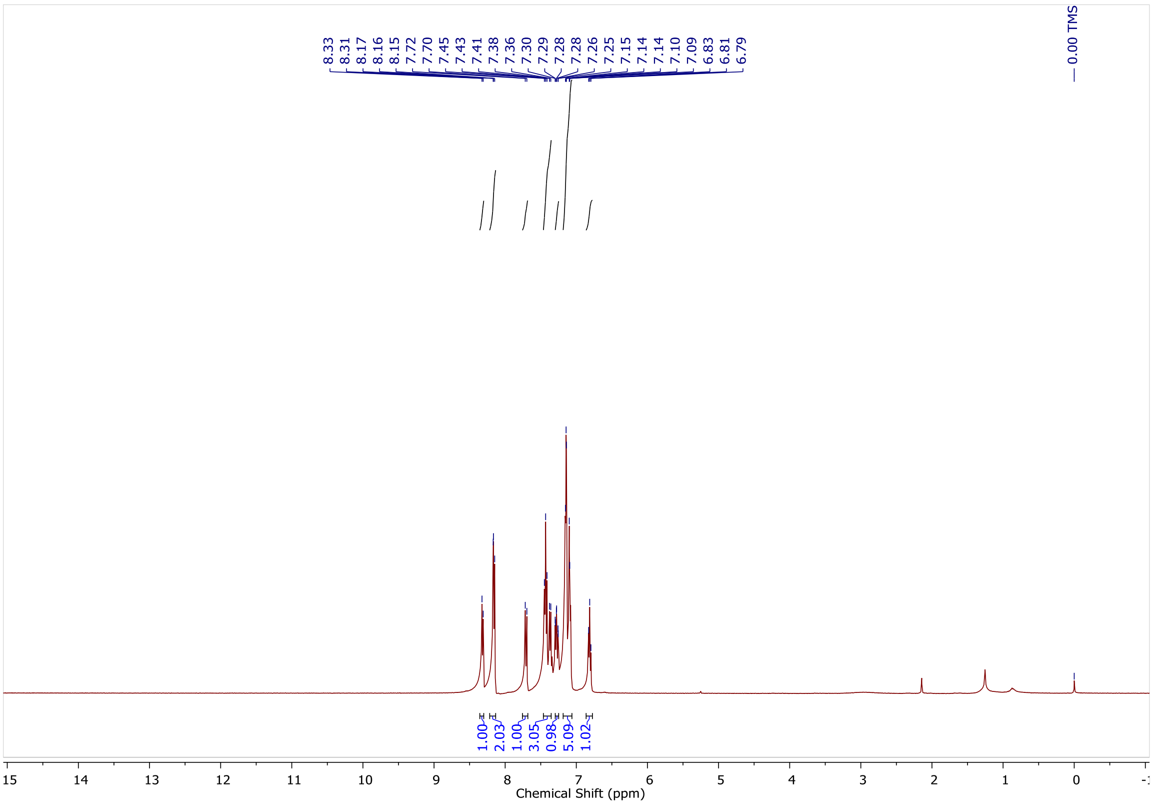

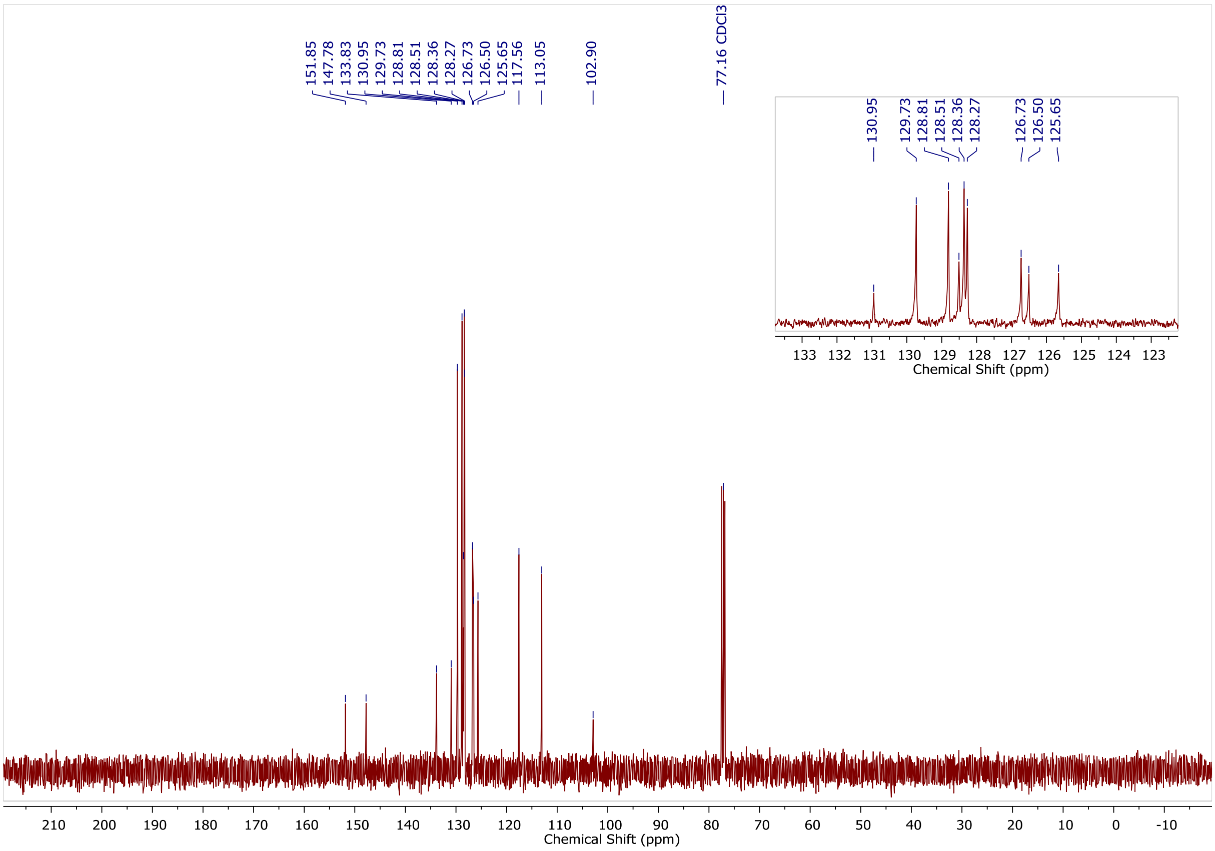

^{1}H NMR (top) ^{13}C NMR (bottom) $CDCl_3$ spectra of compound **3a**

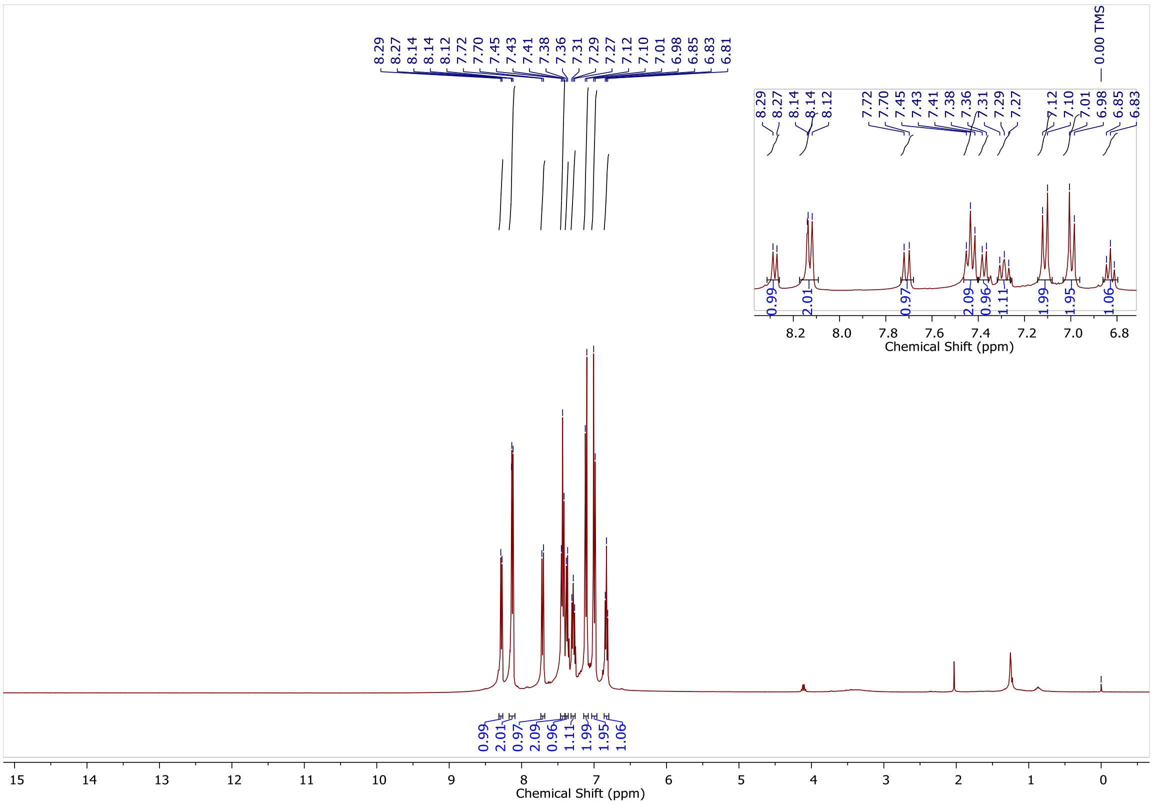

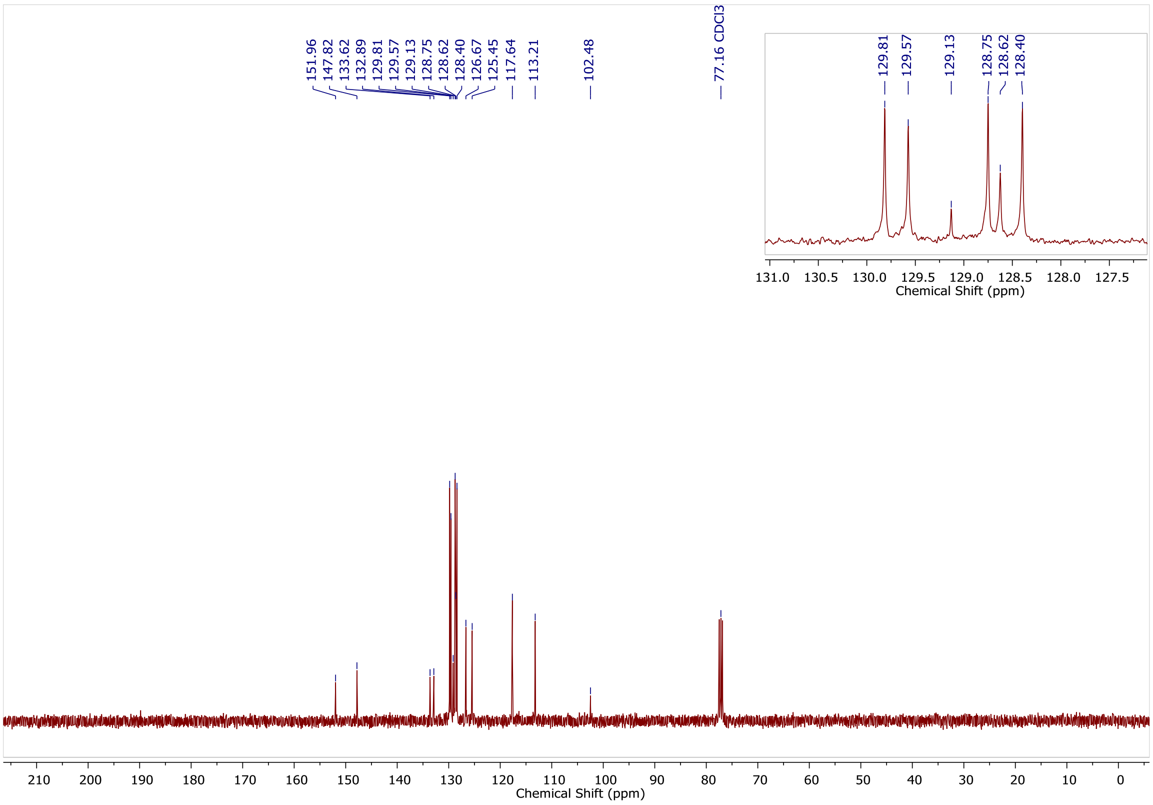

^{1}H NMR (top) ^{13}C NMR (bottom) $CDCl_3$ spectra of compound **3b**

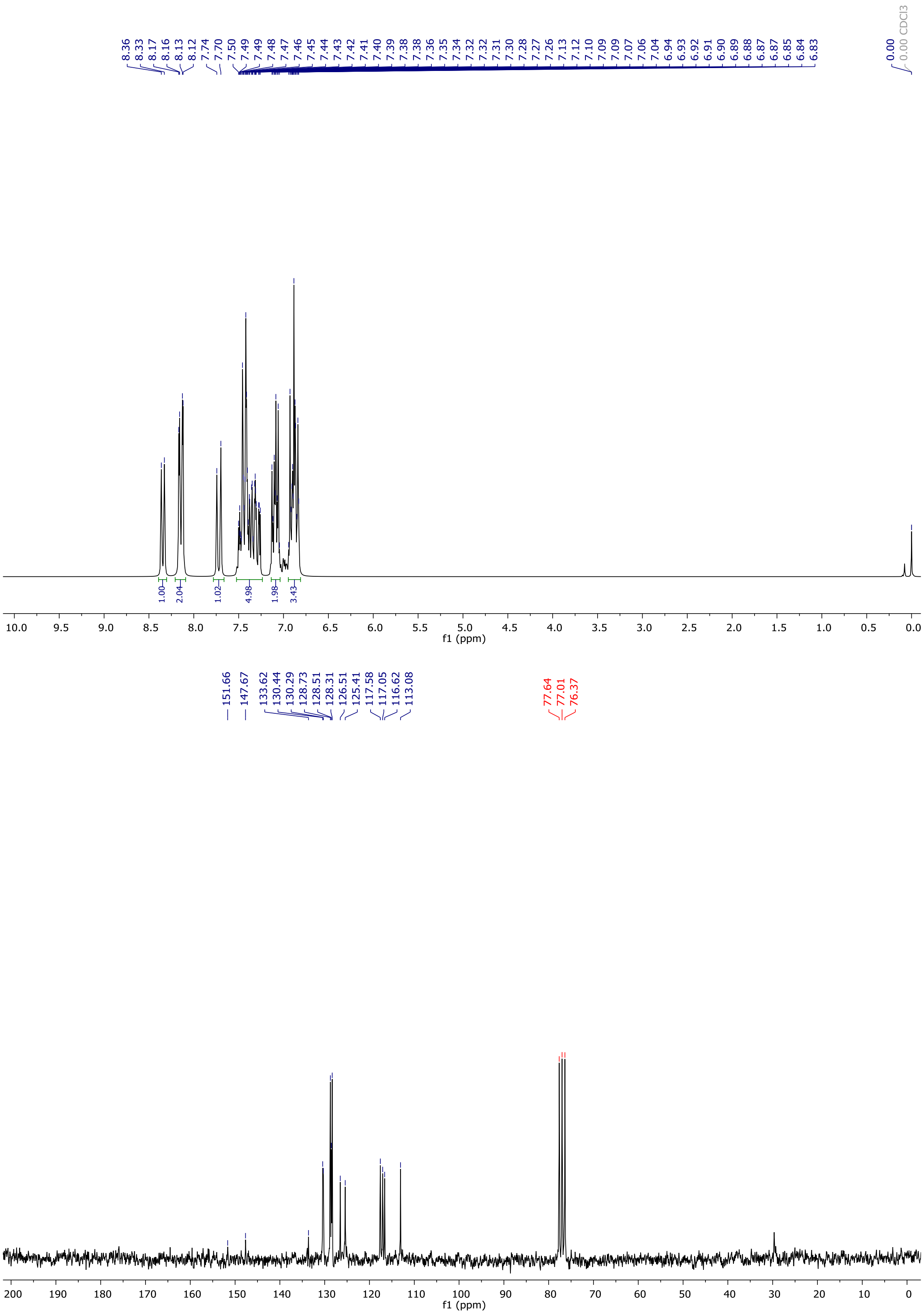

^{1}H NMR (top) ^{13}C NMR (bottom) $CDCl_3$ spectra of compound **3c**

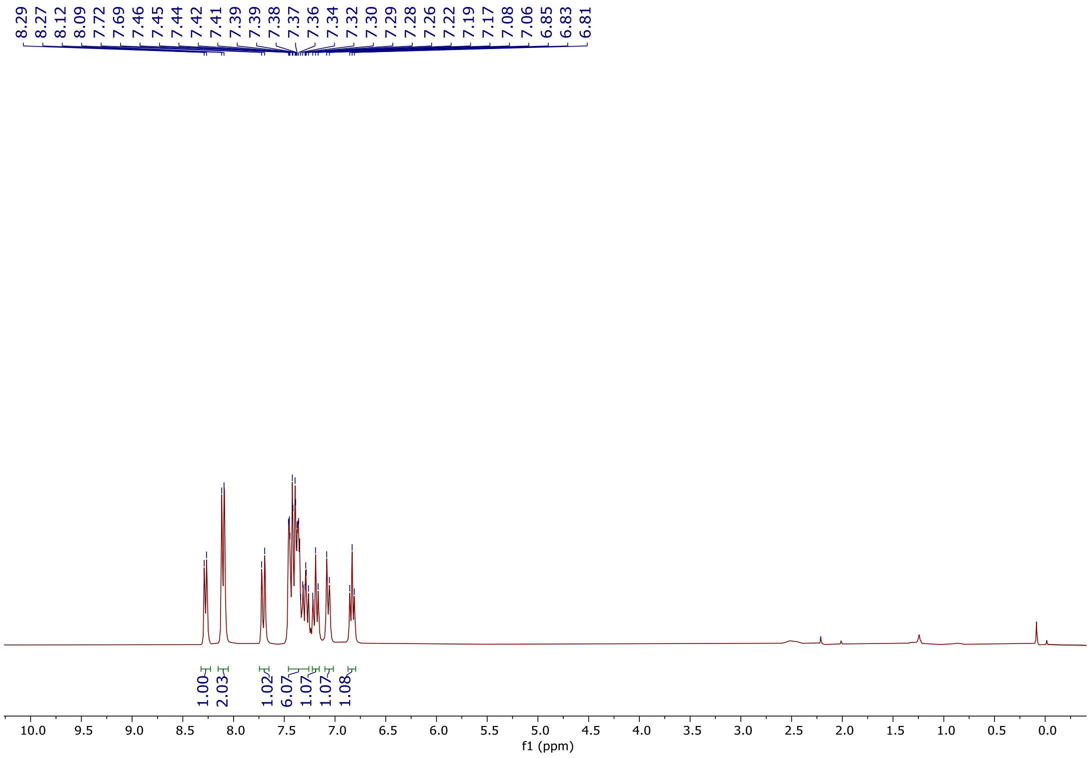

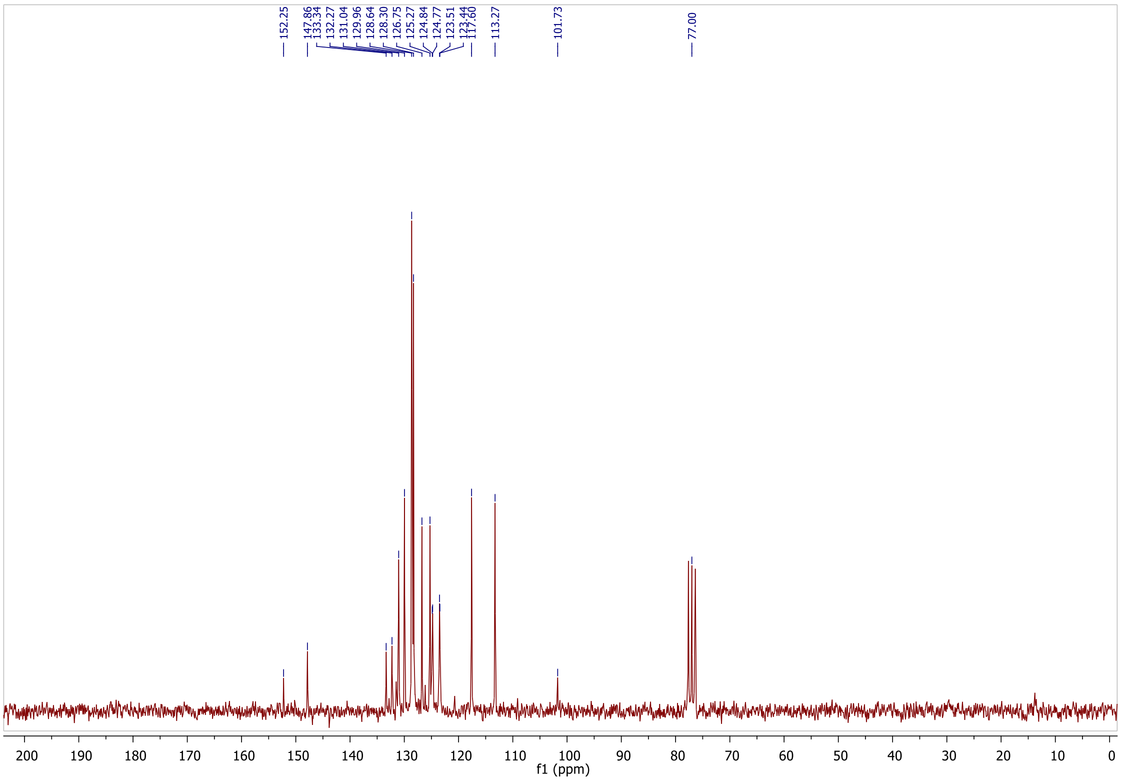

^{1}H NMR (top) ^{13}C NMR (bottom) $CDCl_3$ spectra of compound **3d**

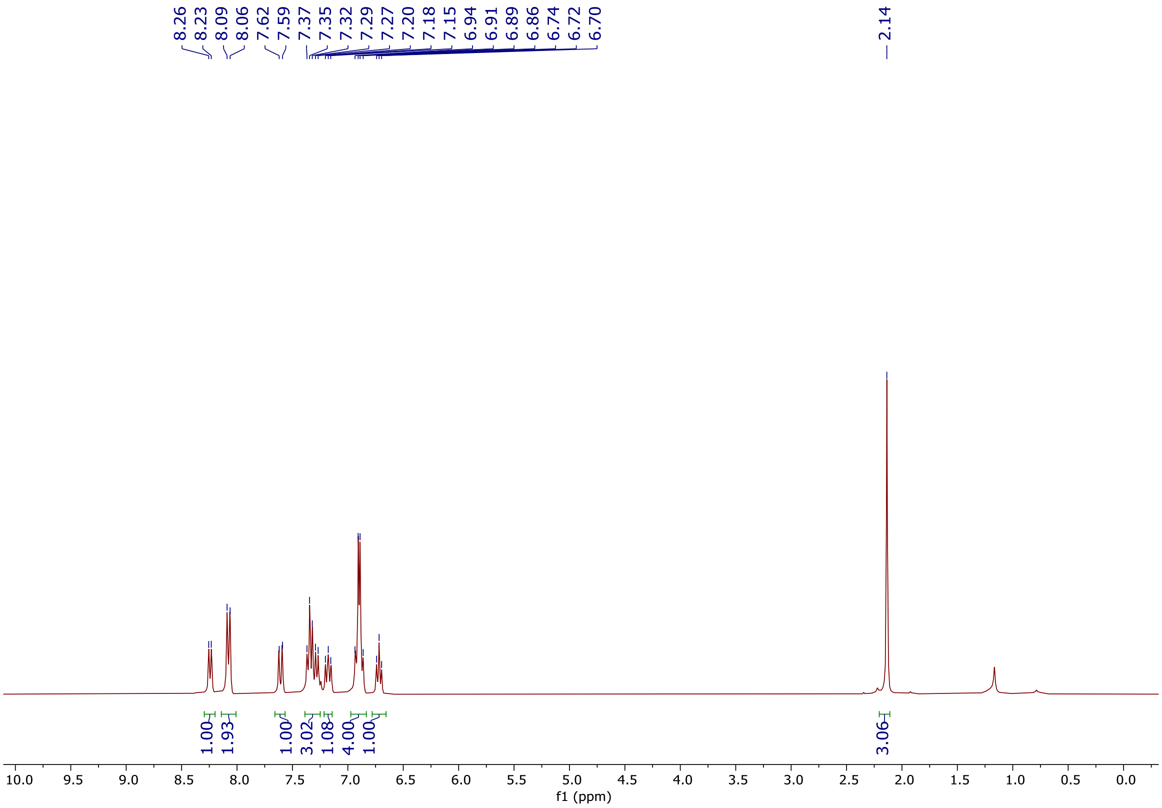

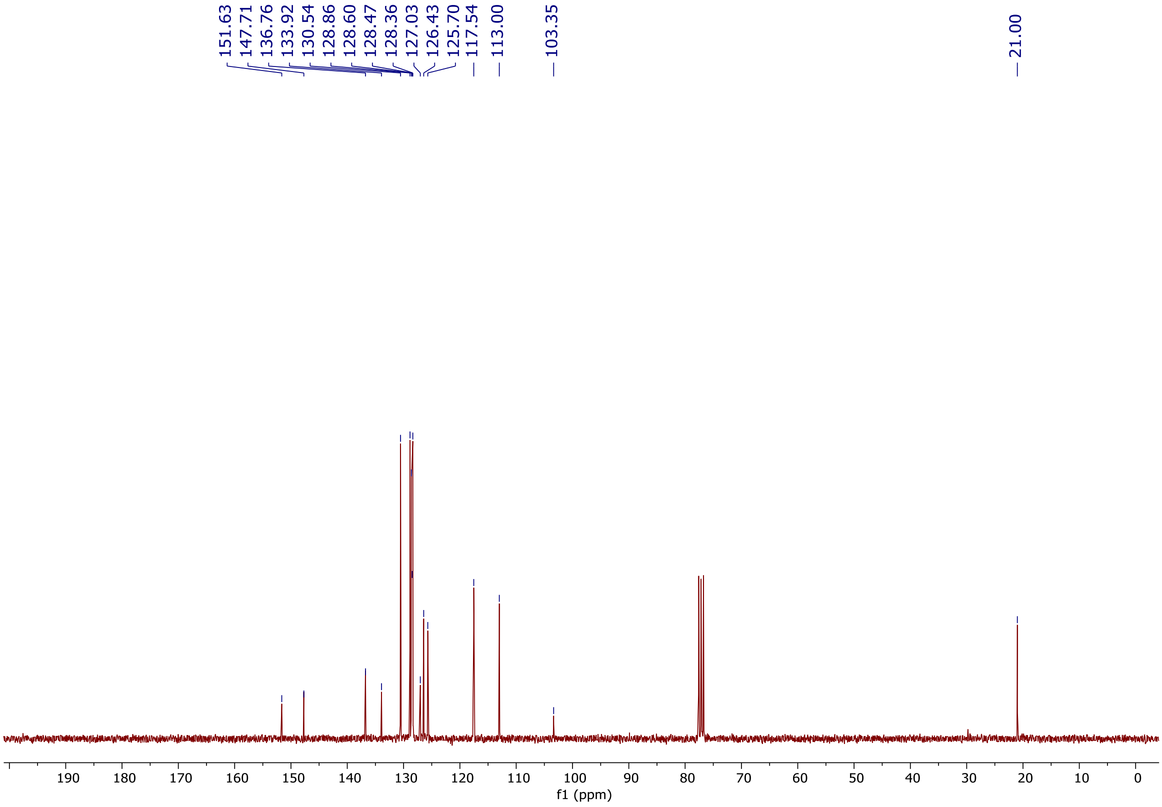

^{1}H NMR (top) ^{13}C NMR (bottom) $CDCl_3$ spectra of compound **3e**

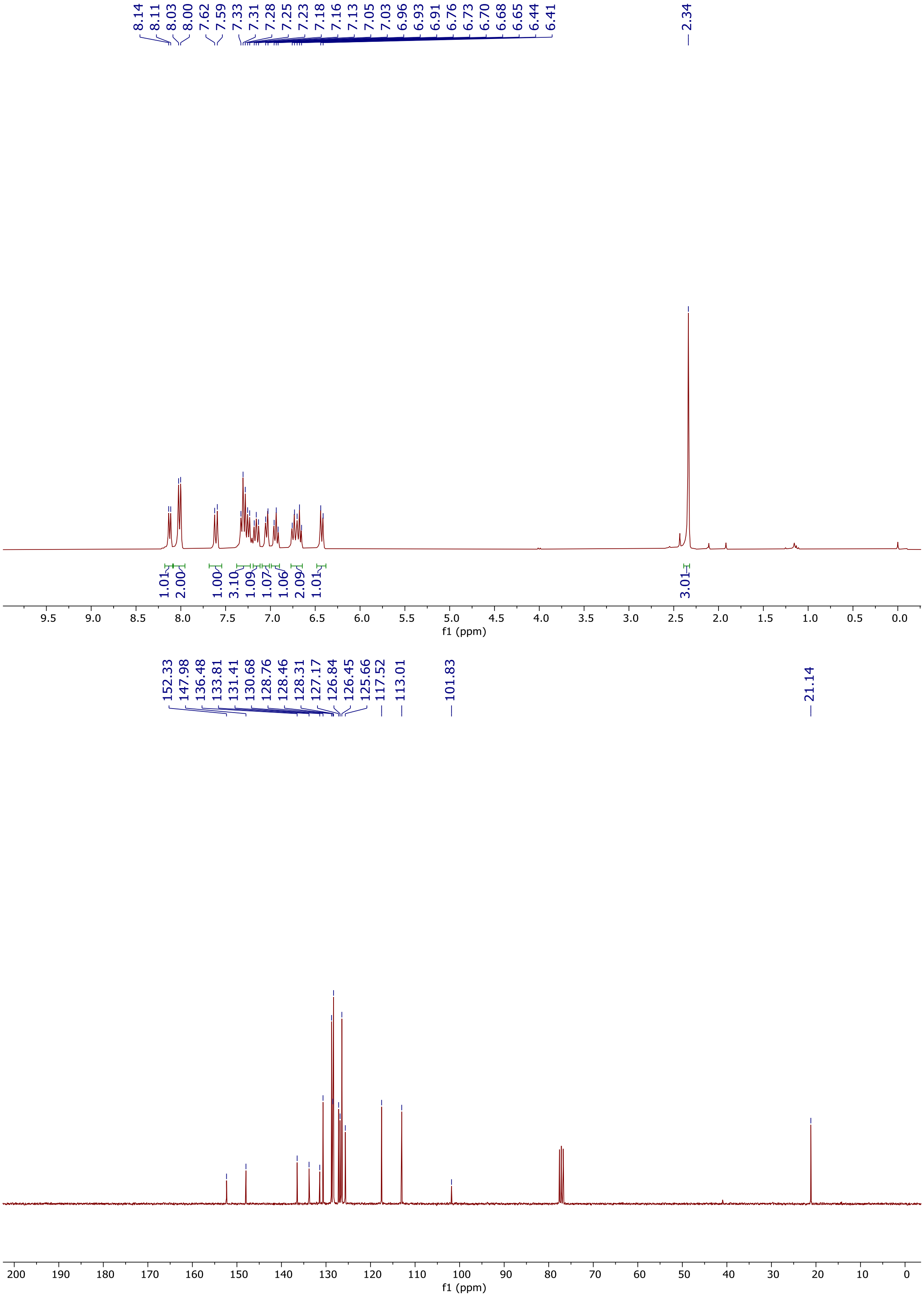

^{1}H NMR (top) ^{13}C NMR (bottom) $CDCl_3$ spectra of compound **3f**

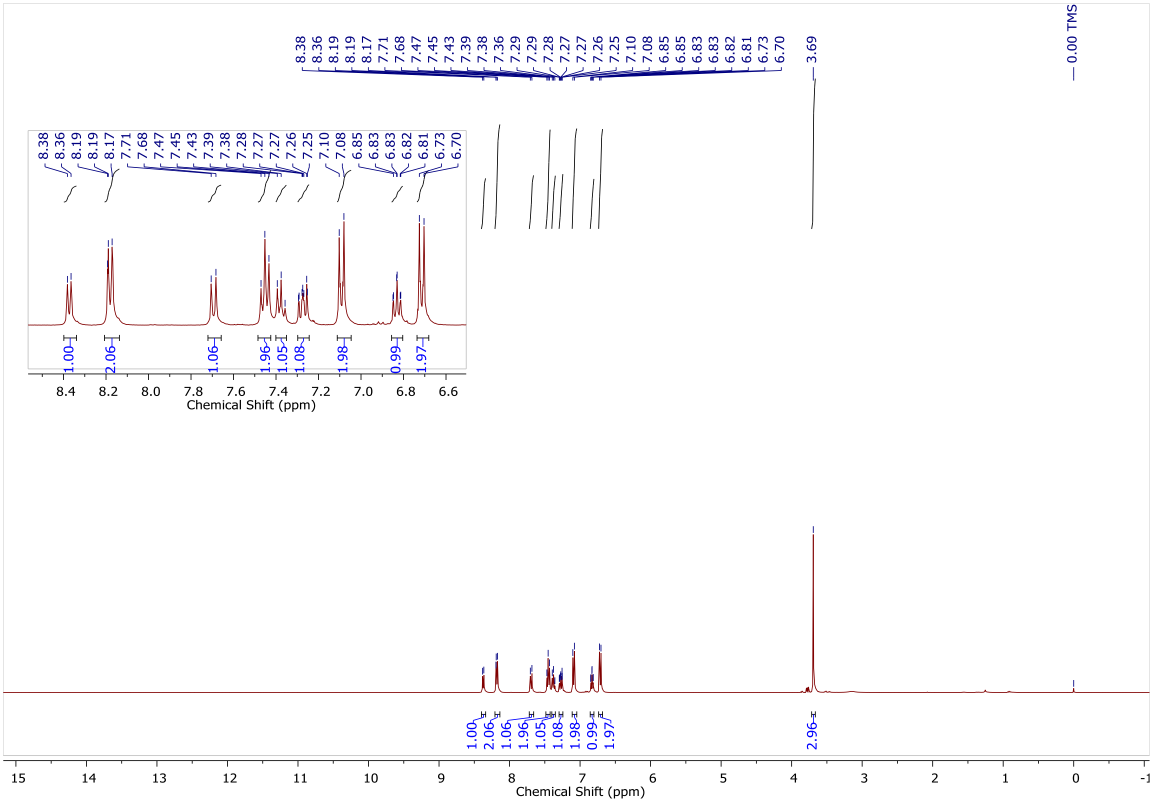

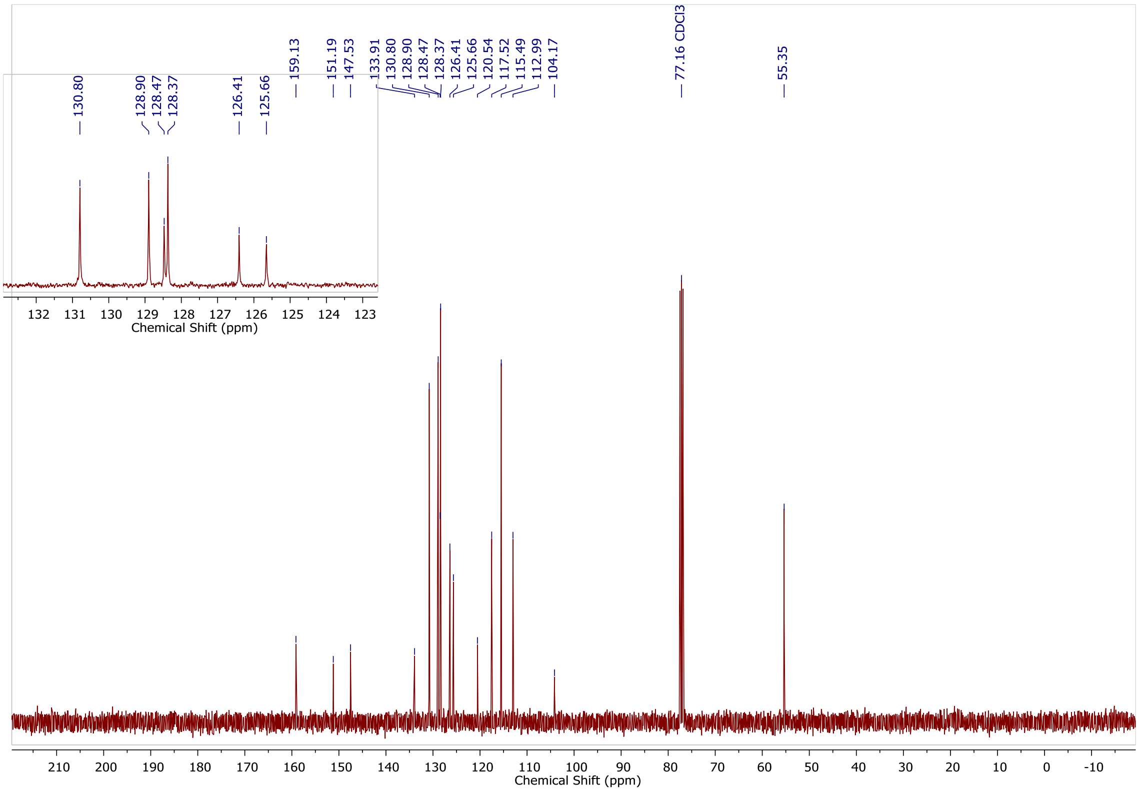

^{1}H NMR (top) ^{13}C NMR (bottom) $CDCl_3$ spectra of compound **3g**

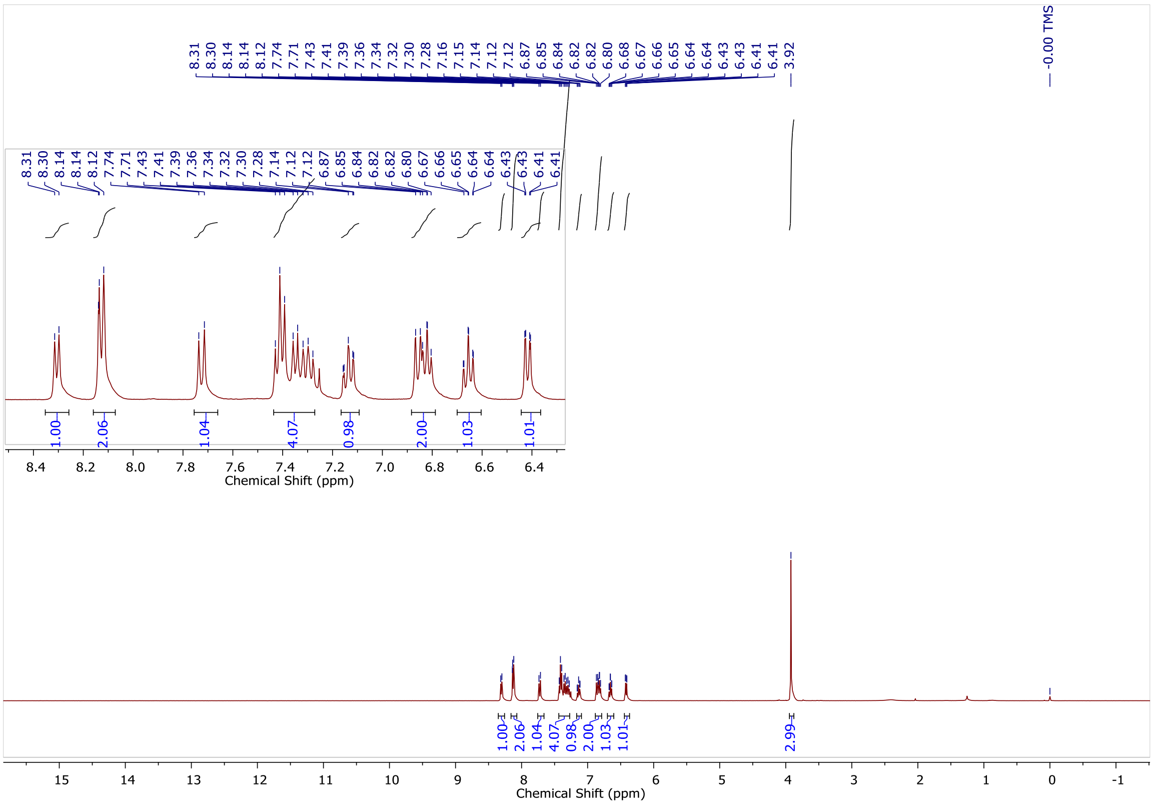

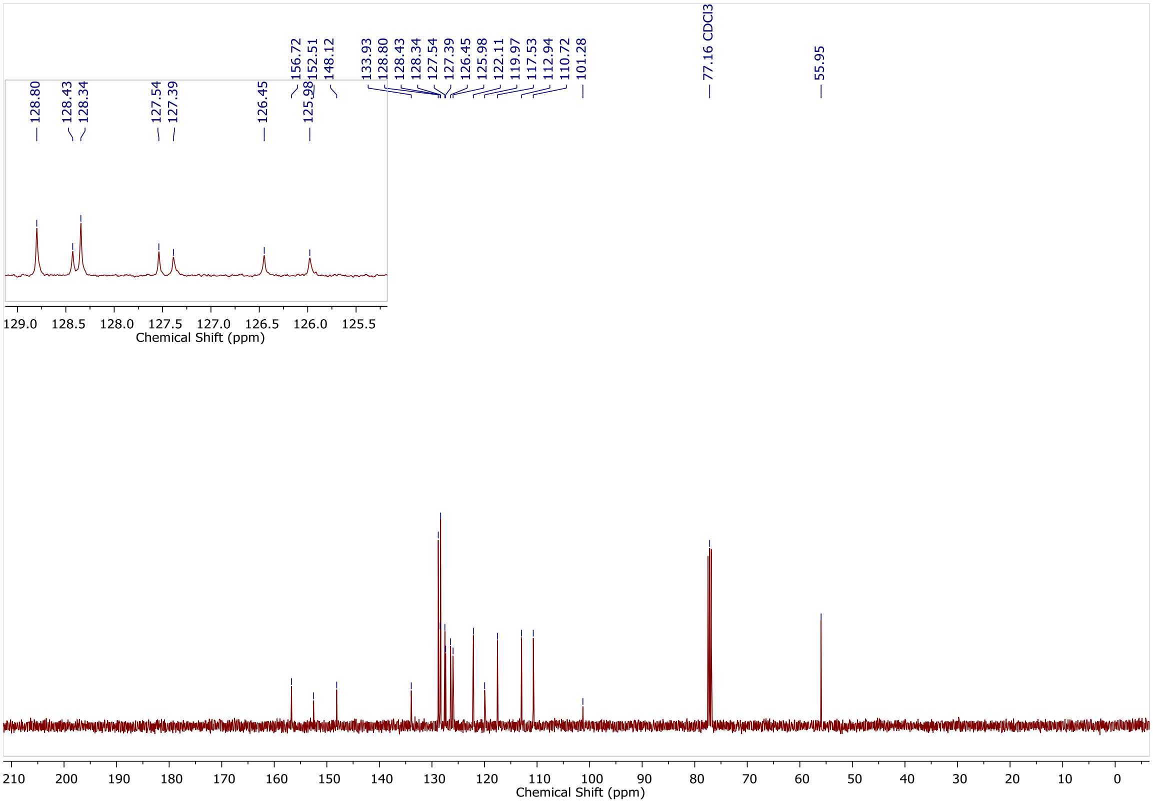

^{1}H NMR (top) ^{13}C NMR (bottom) $CDCl_3$ spectra of compound **3h**

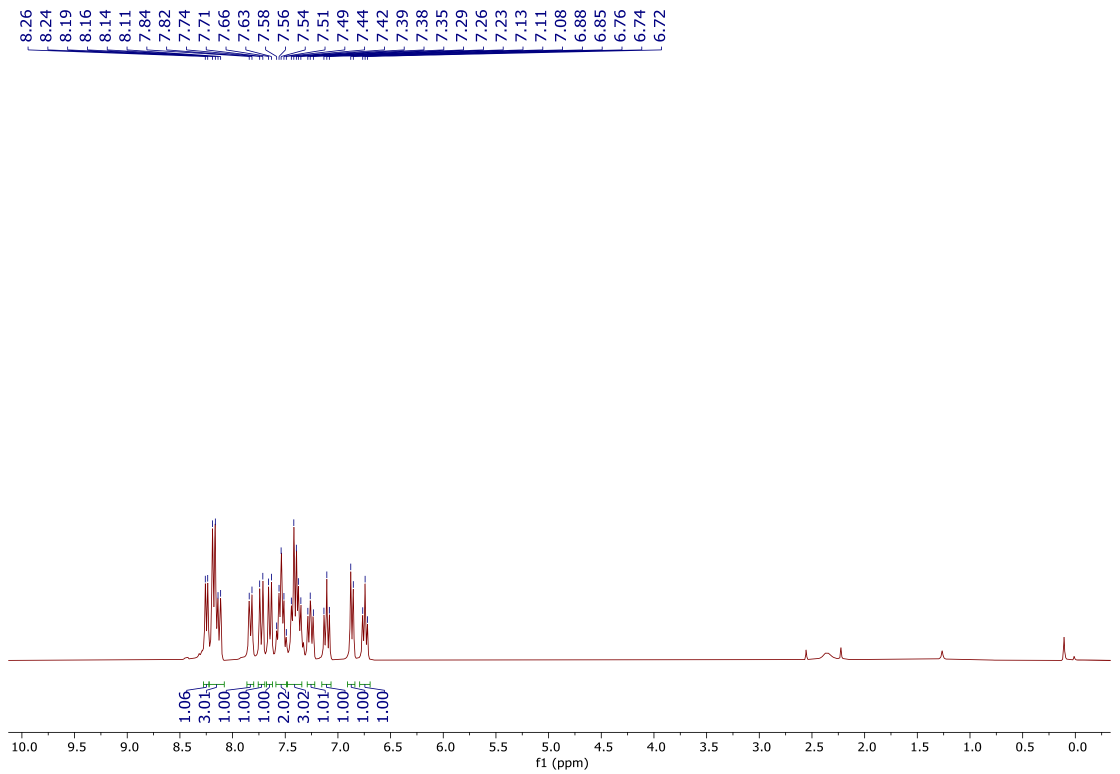

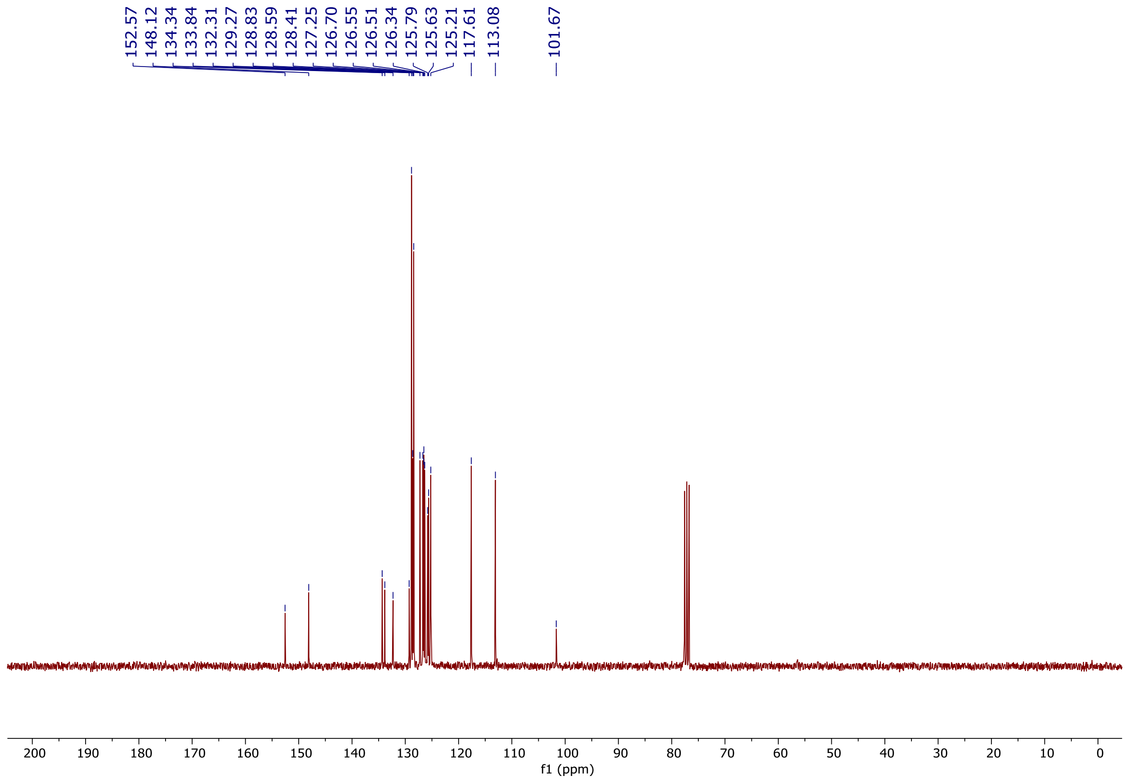

^1H NMR (top) ^{13}C NMR (bottom) $CDCl_3$ spectra of compound **3i**

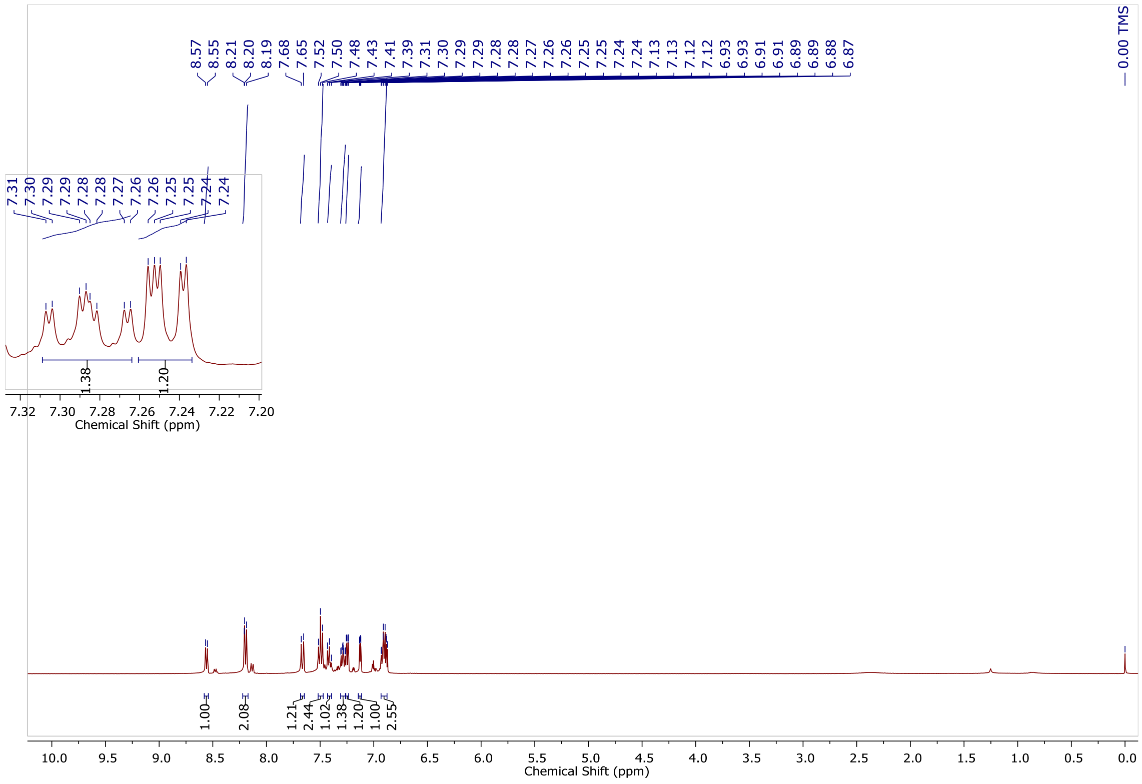

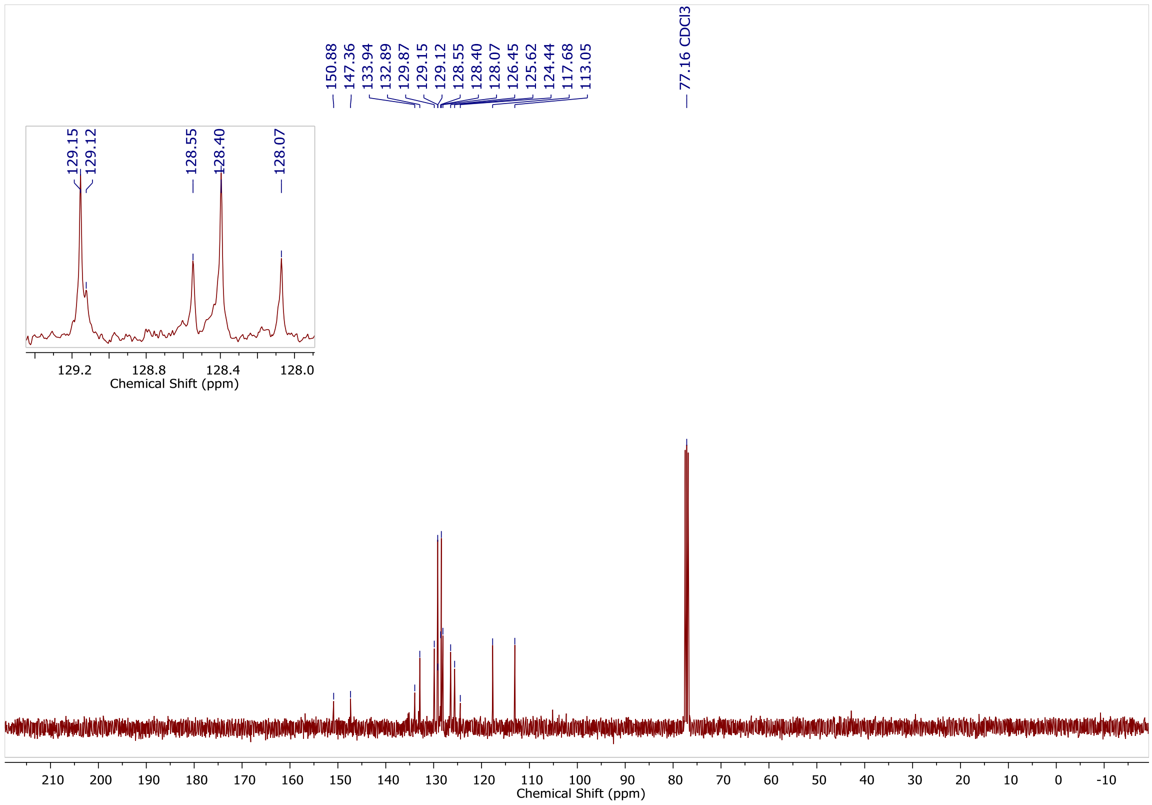

^{1}H NMR (top) ^{13}C NMR (bottom) $CDCl_3$ spectra of compound **3j**

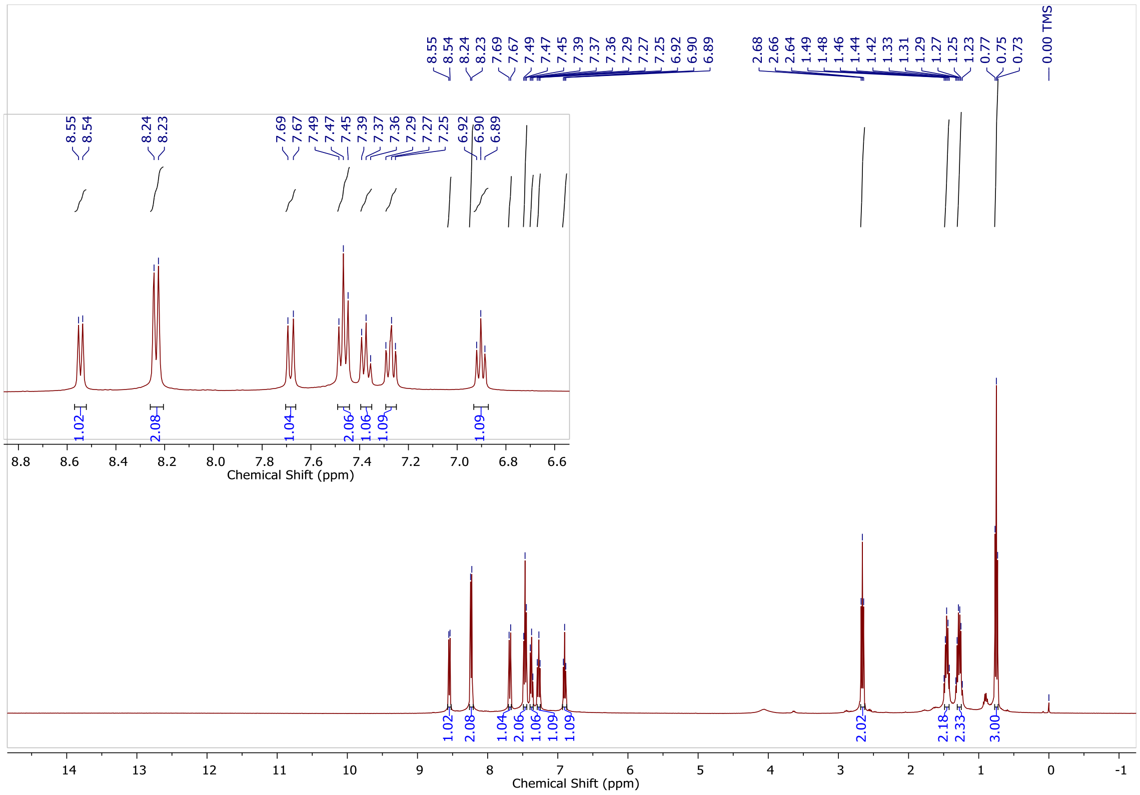

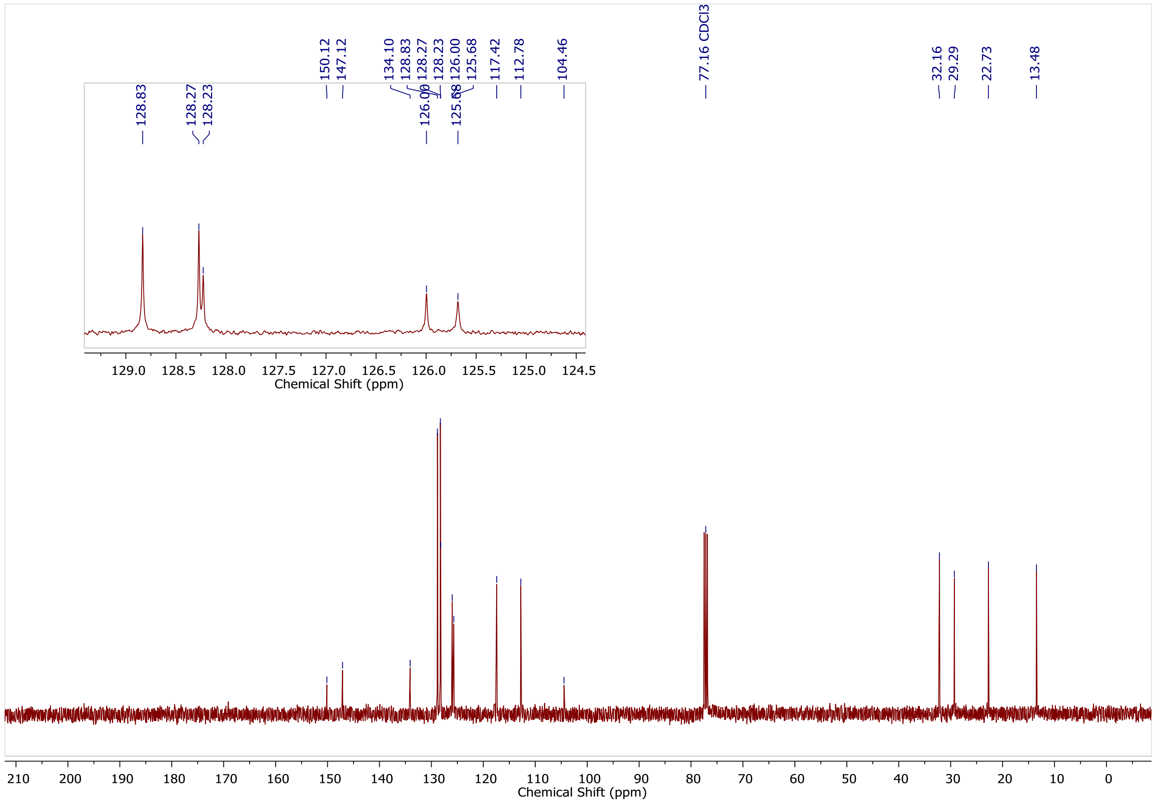

^{1}H NMR (top) ^{13}C NMR (bottom) $CDCl_3$ spectra of compound **3k**

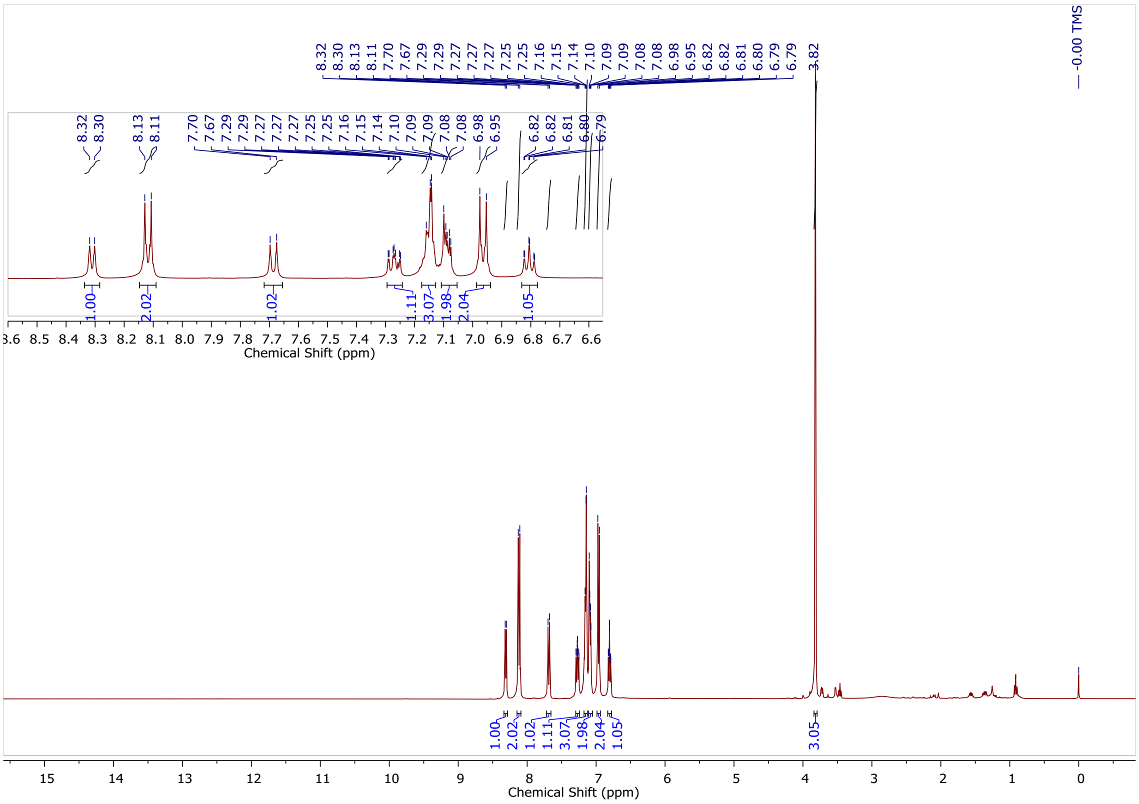

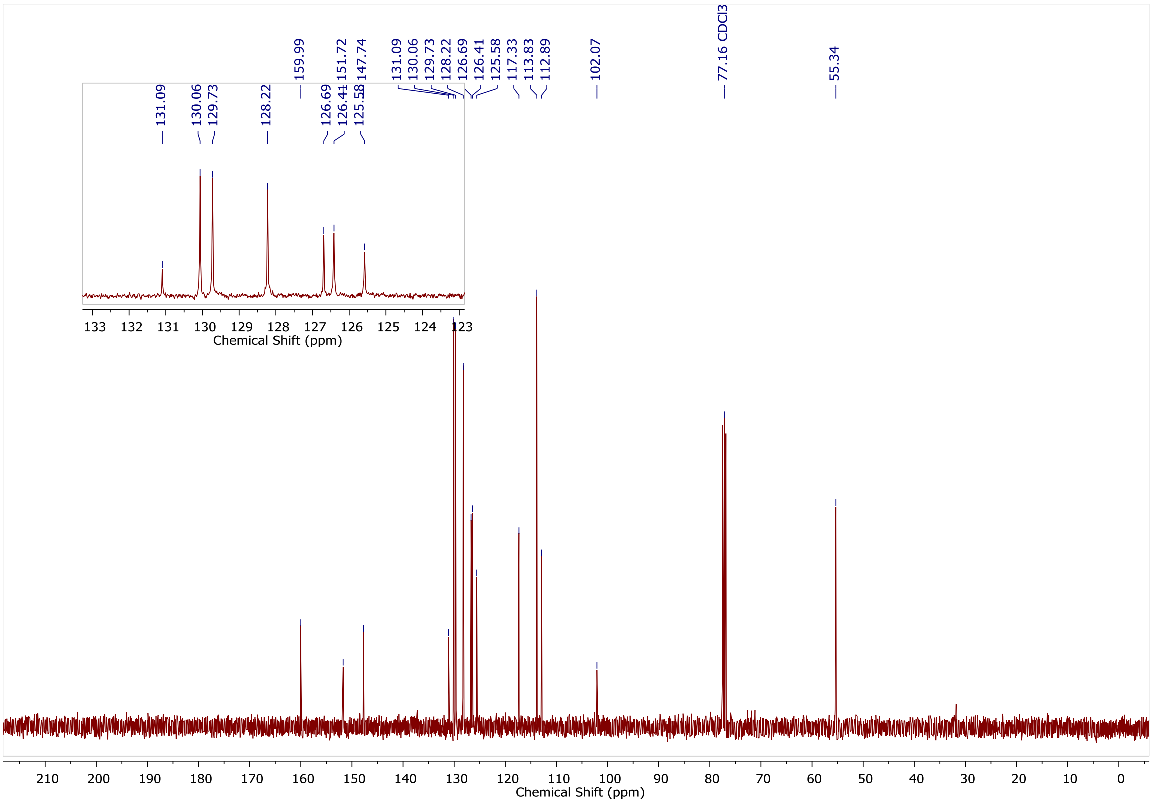

^{1}H NMR (top) ^{13}C NMR (bottom) $CDCl_3$ spectra of compound **4a**

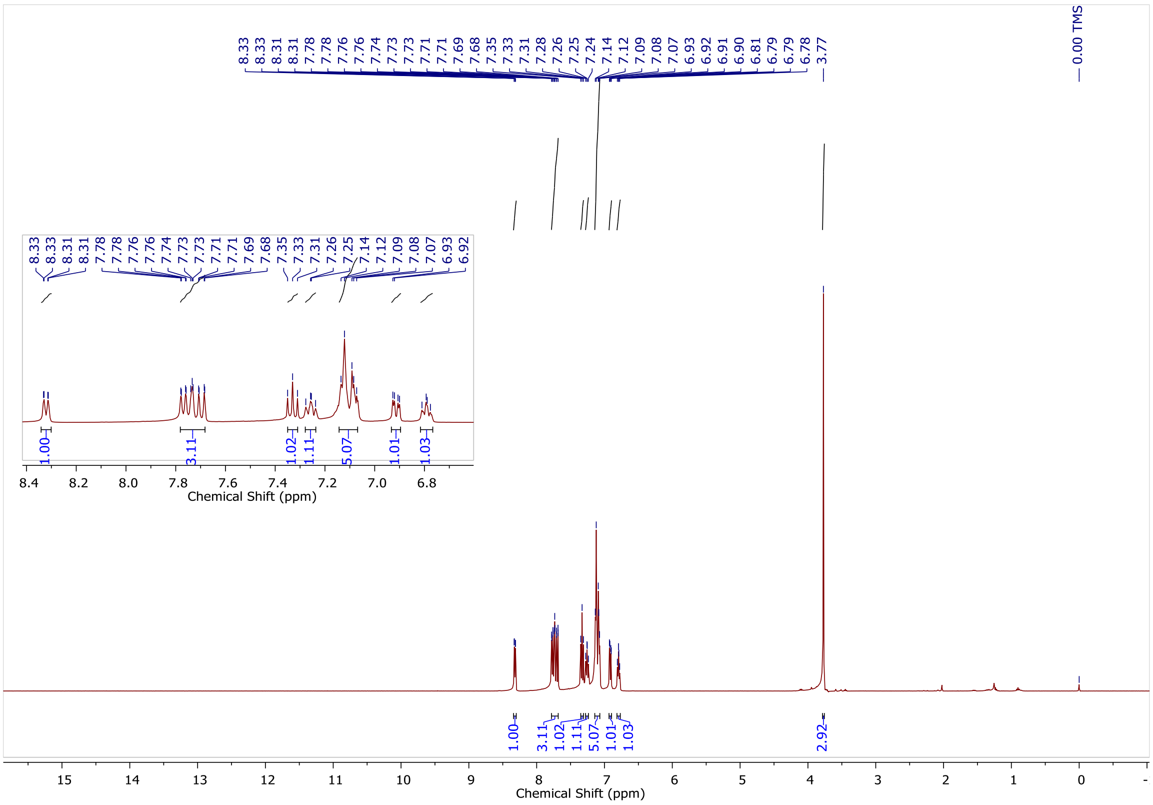

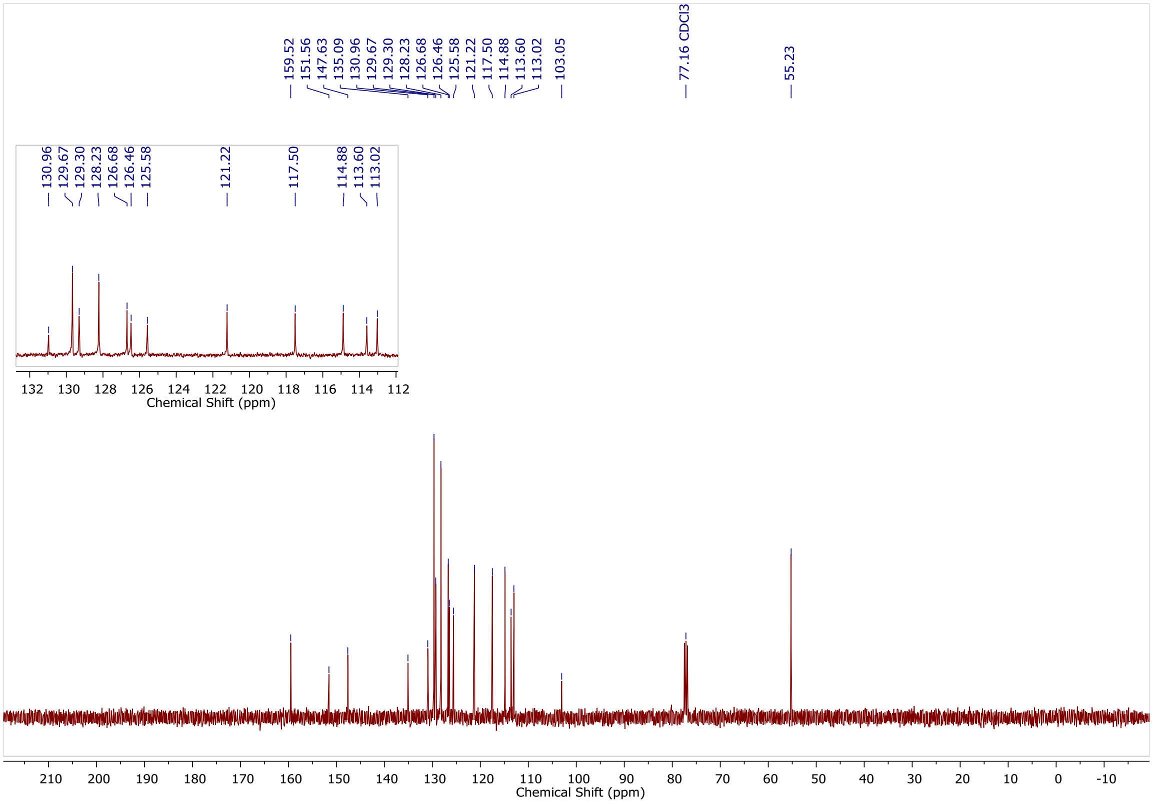

^{1}H NMR (top) ^{13}C NMR (bottom) $CDCl_3$ spectra of compound **4b**

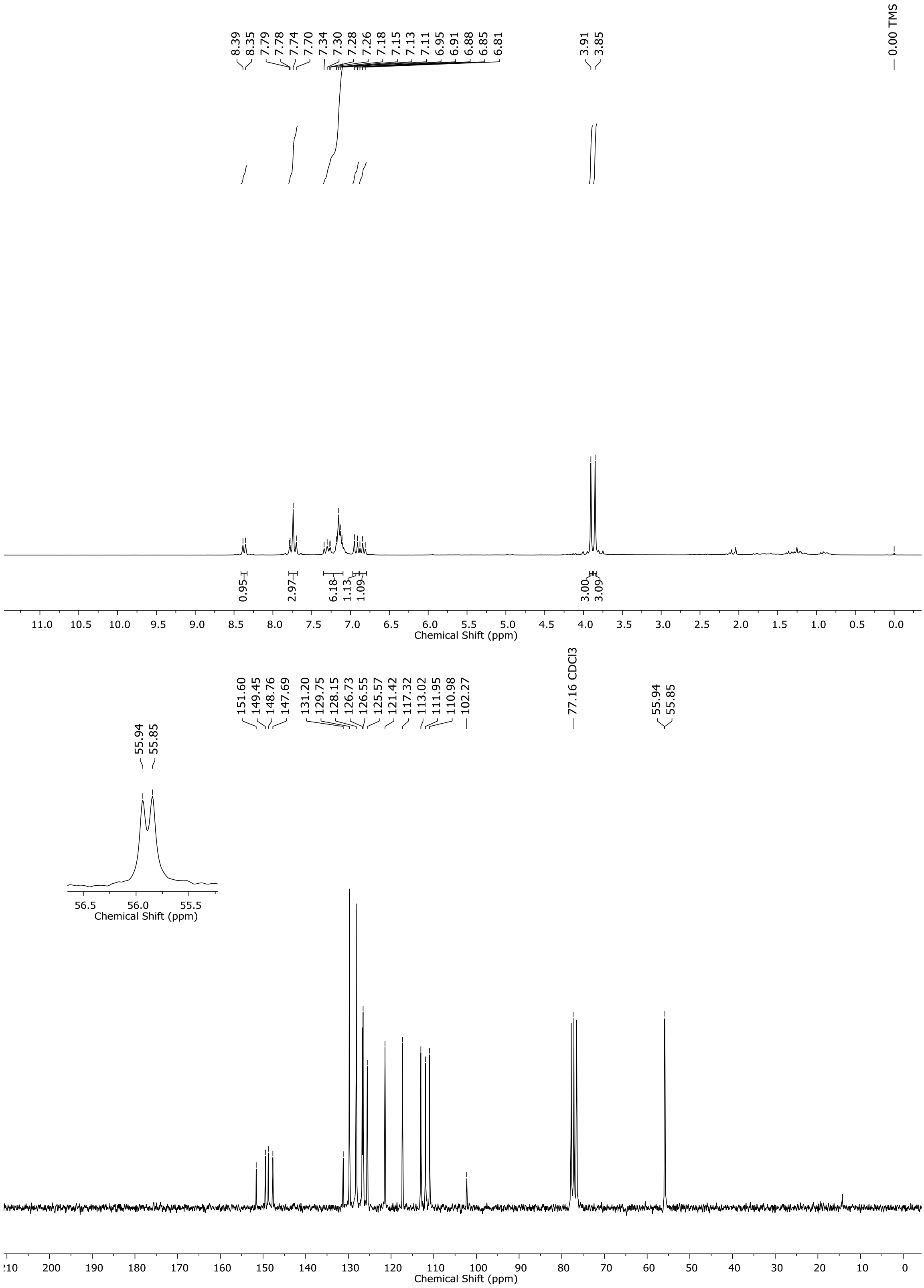

^{1}H NMR (top) ^{13}C NMR (bottom) $CDCl_3$ spectra of compound **4c**

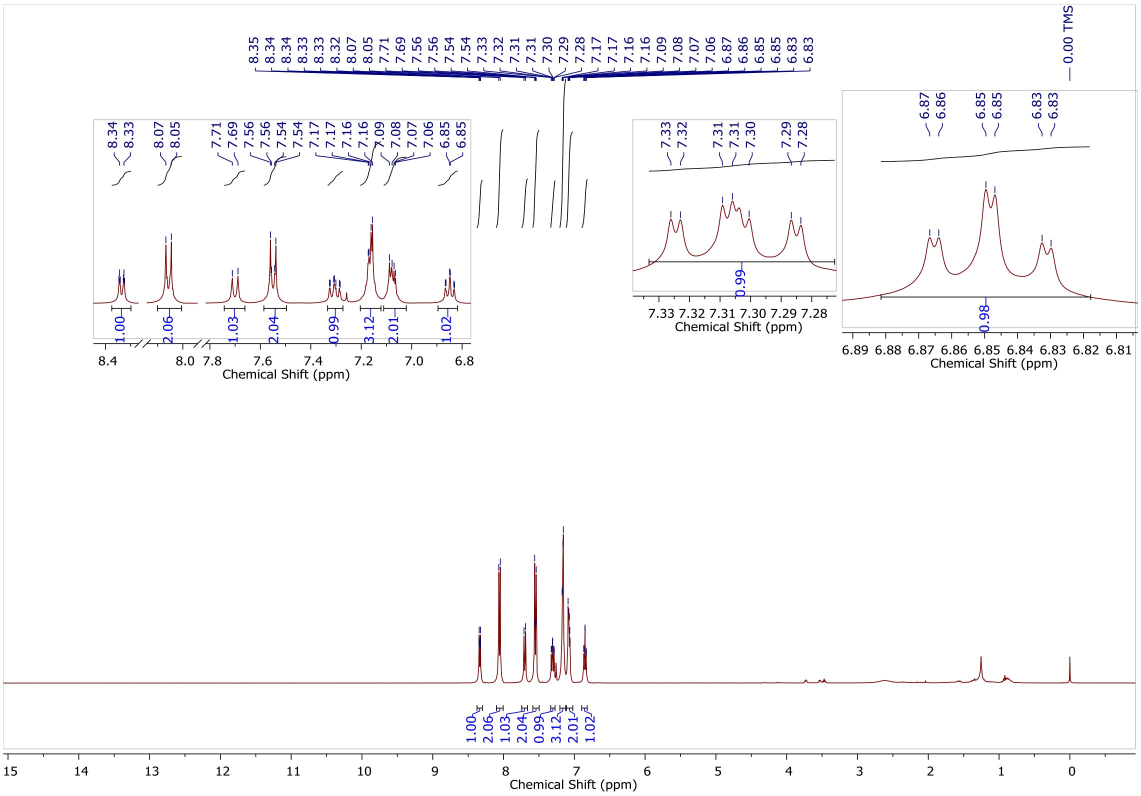

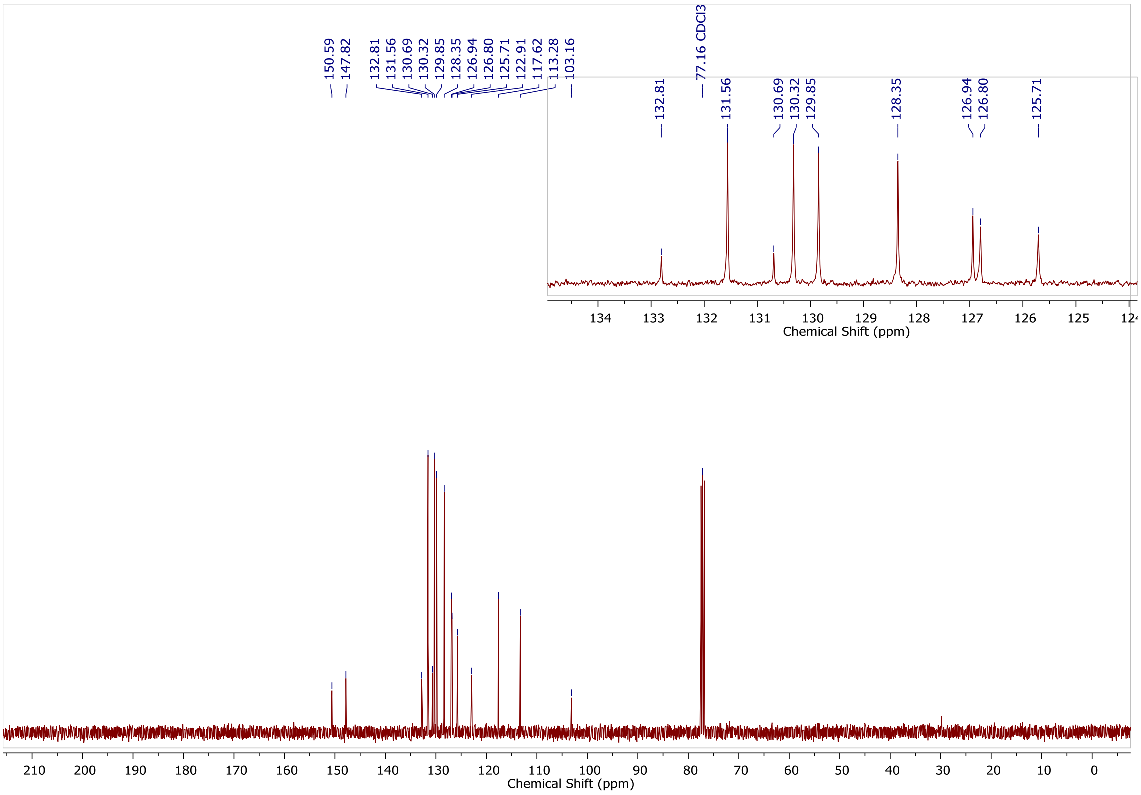

^{1}H NMR (top) ^{13}C NMR (bottom) $CDCl_3$ spectra of compound **4d**

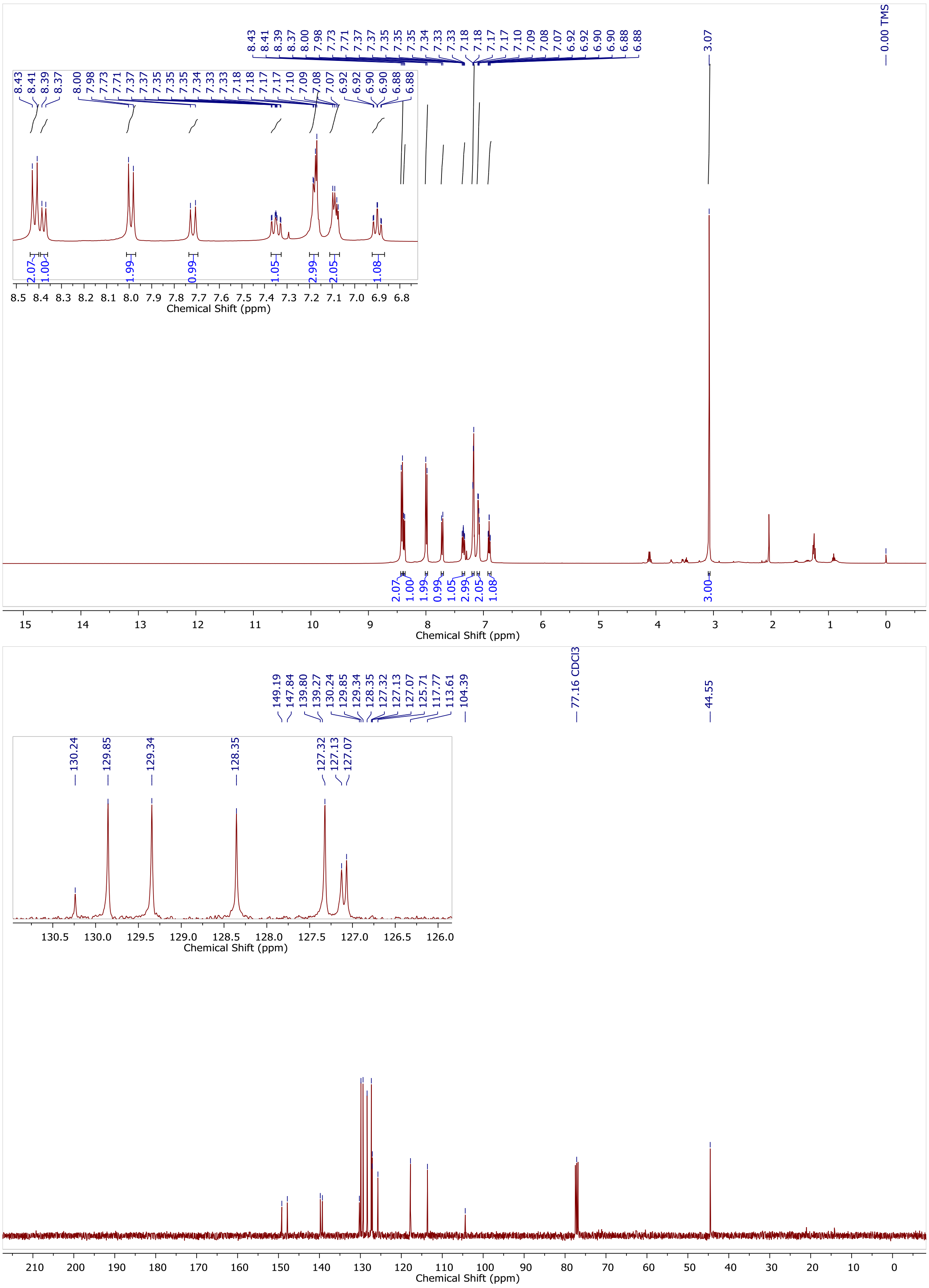

^{1}H NMR (top) ^{13}C NMR (bottom) $CDCl_3$ spectra of compound **4e**

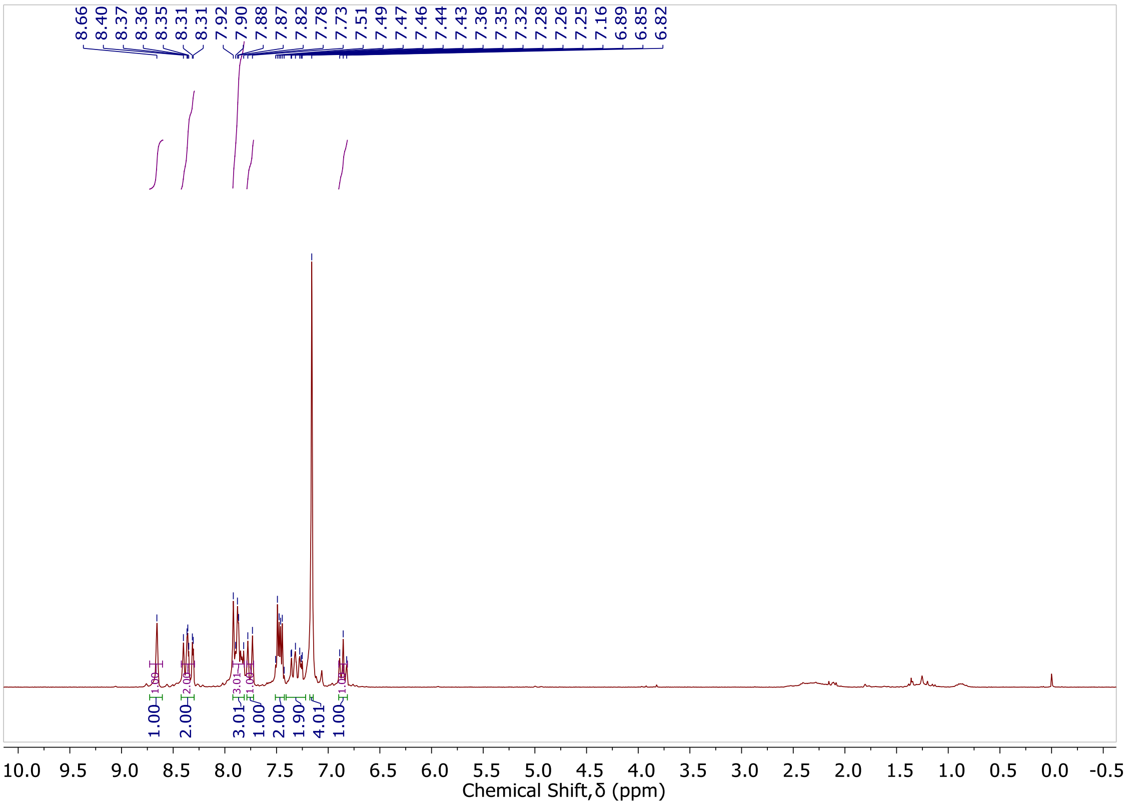

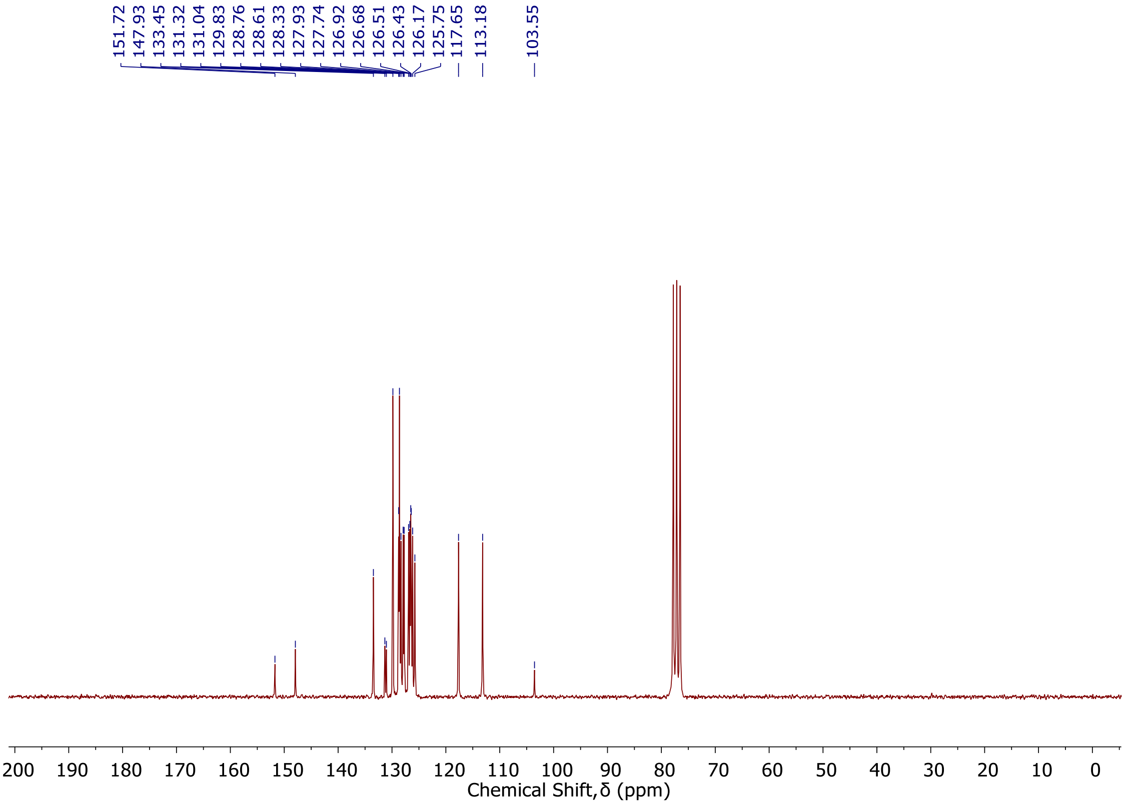

^{1}H NMR (top) ^{13}C NMR (bottom) $CDCl_3$ spectra of compound **4f**

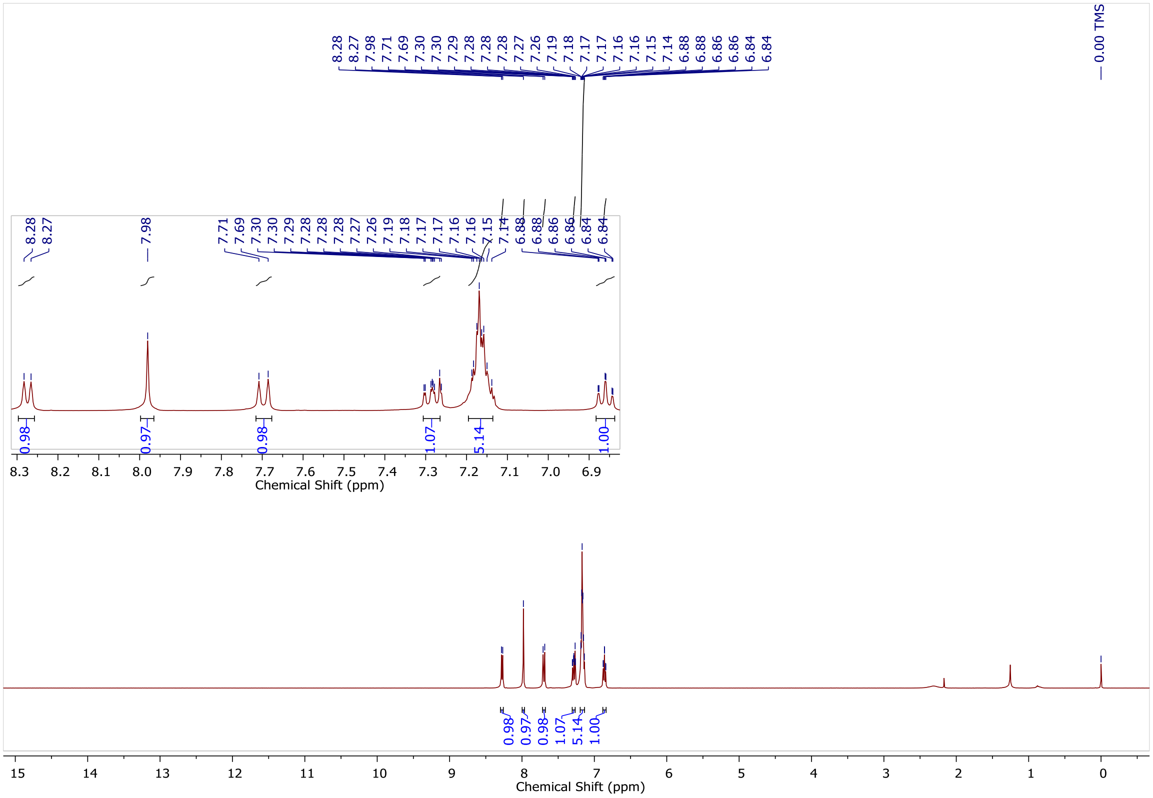

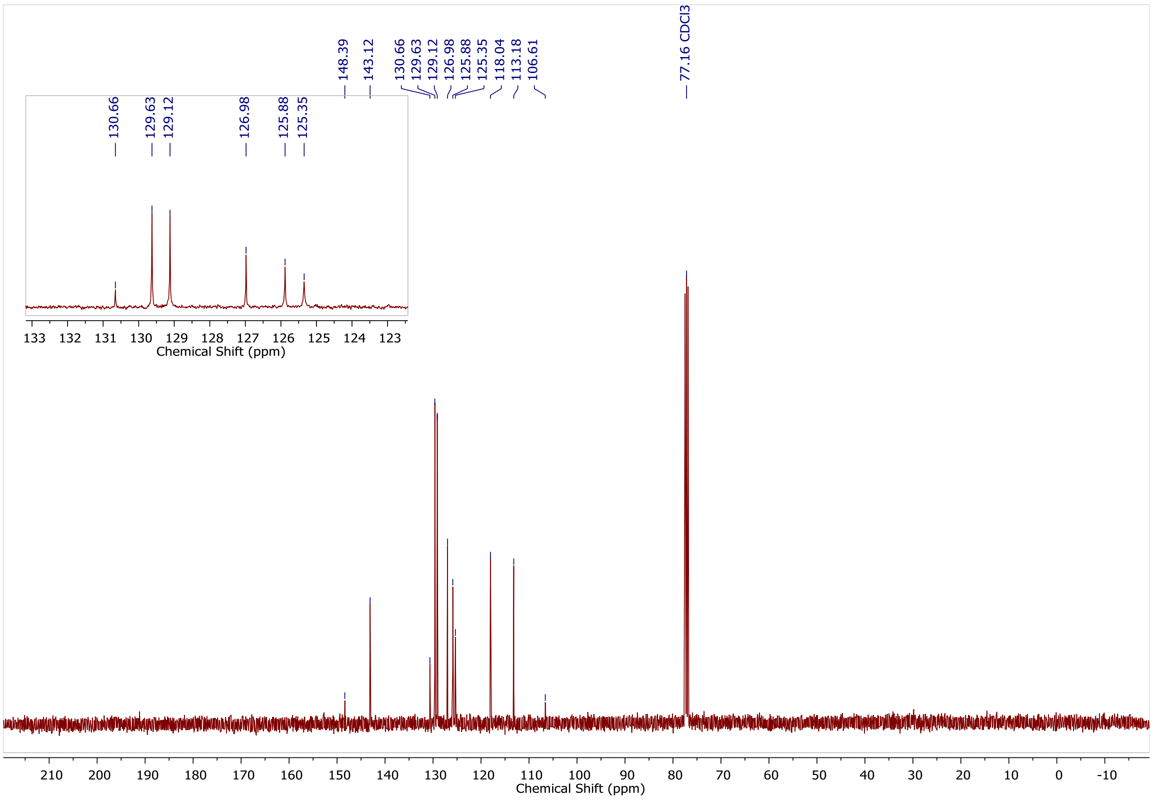

^{1}H NMR (top) ^{13}C NMR (bottom) $CDCl_3$ spectra of compound **4g**

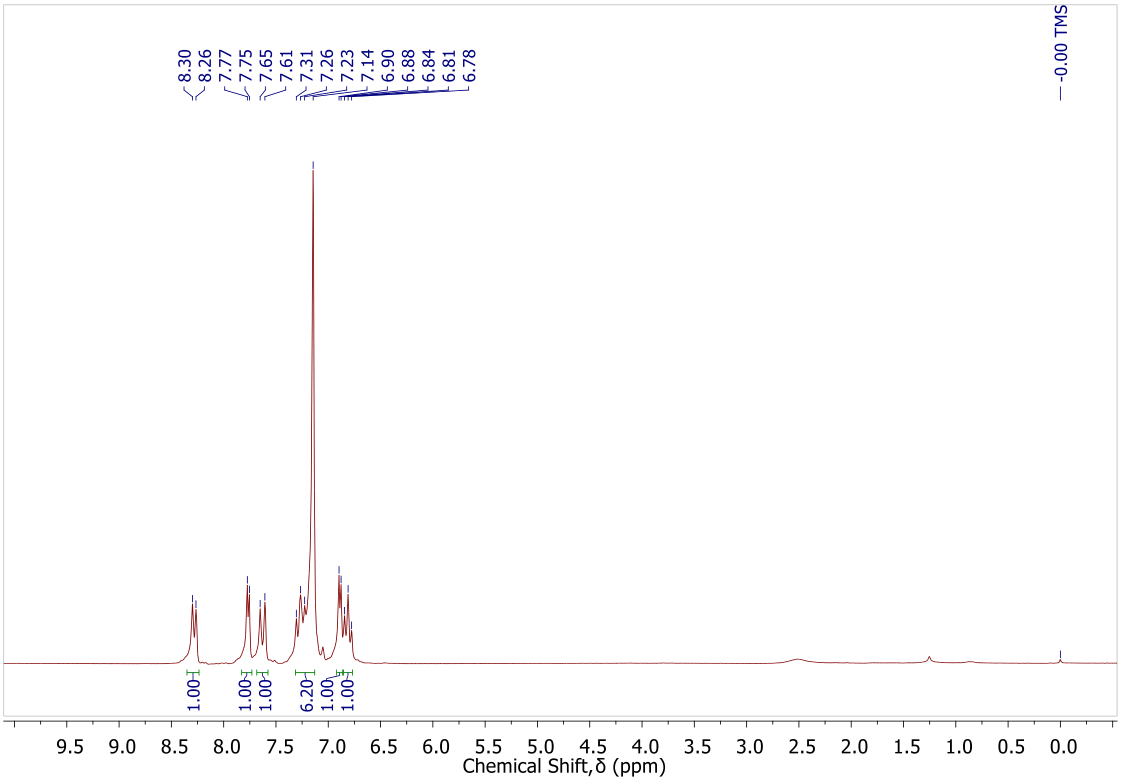

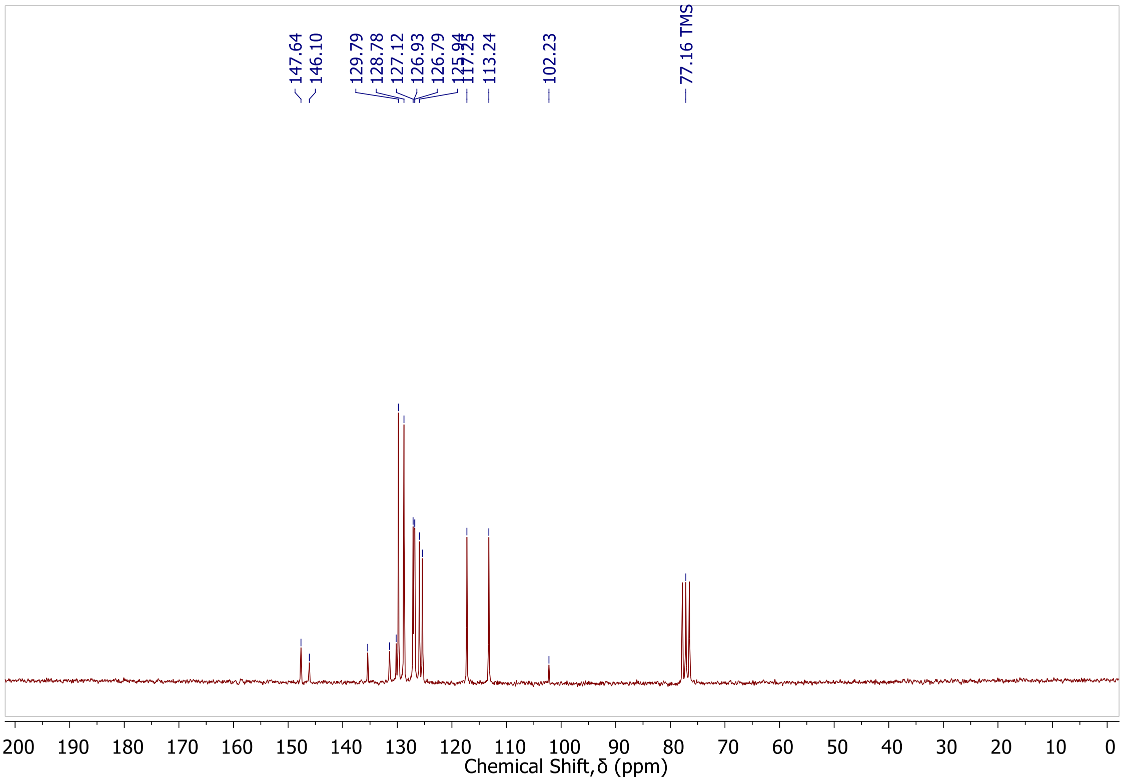

^{1}H NMR (top) ^{13}C NMR (bottom) $CDCl_3$ spectra of compound **4h**

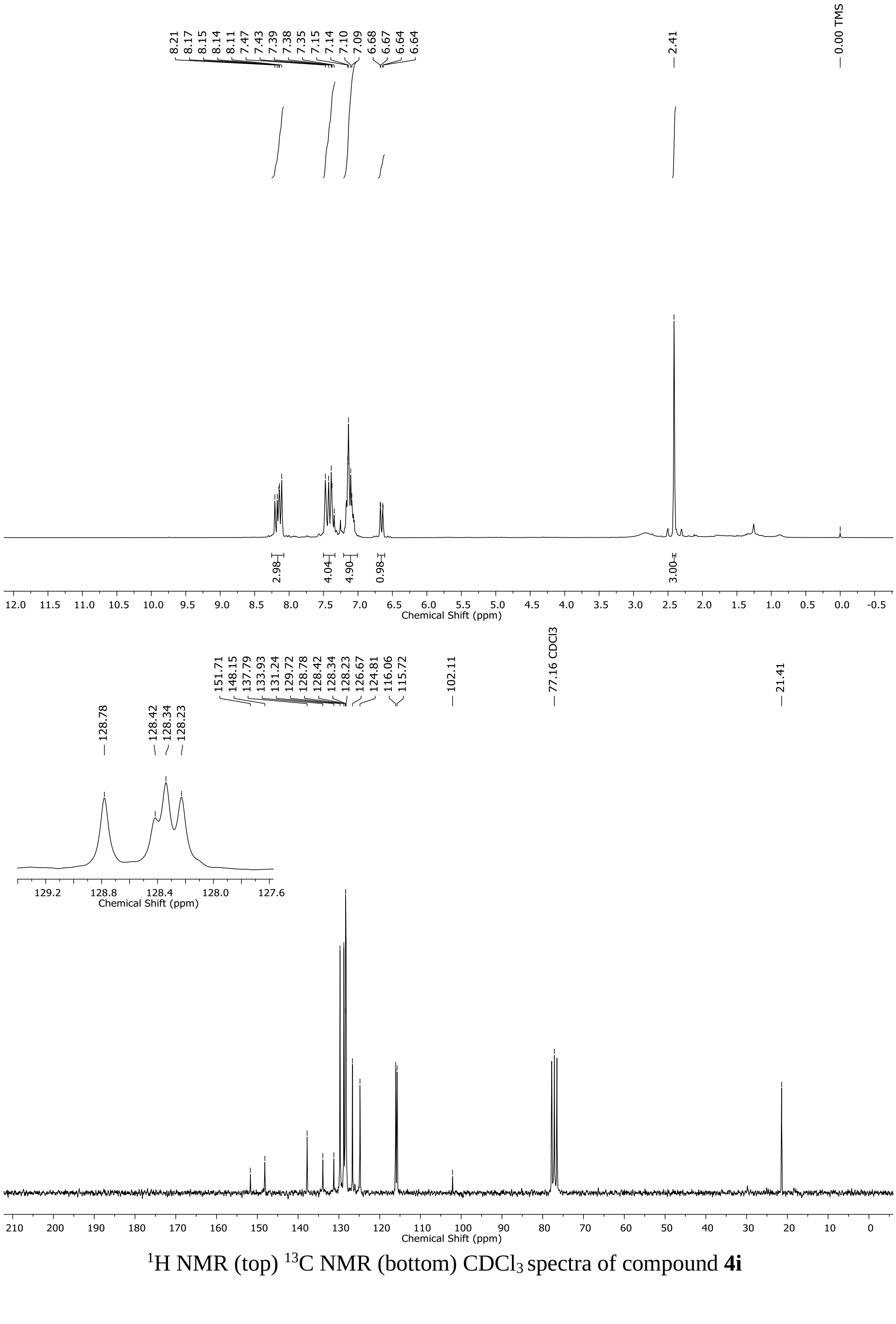

^{1}H NMR (top) ^{13}C NMR (bottom) $CDCl_3$ spectra of compound **4i**

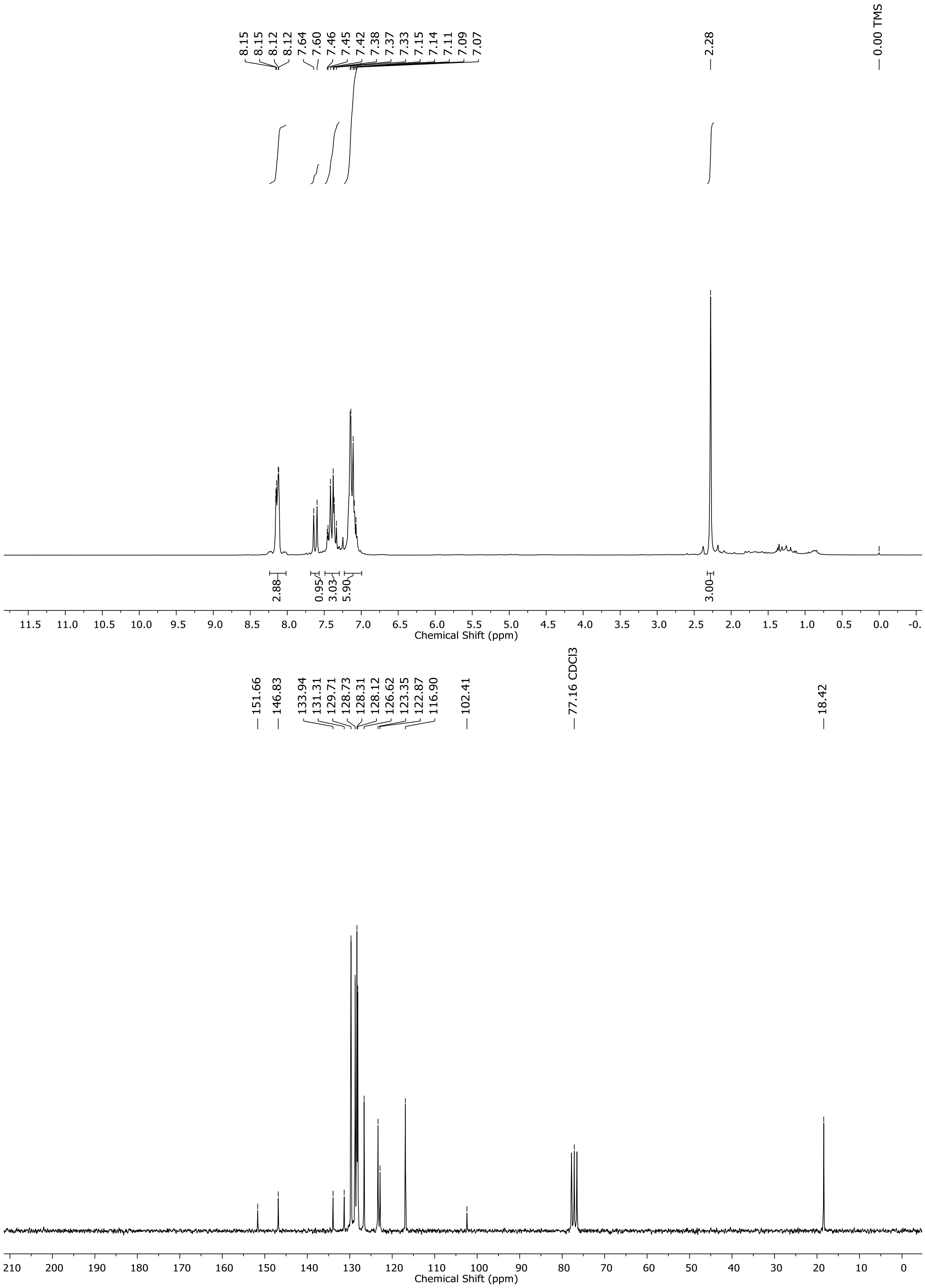

^{1}H NMR (top) ^{13}C NMR (bottom) $CDCl_3$ spectra of compound **4j**

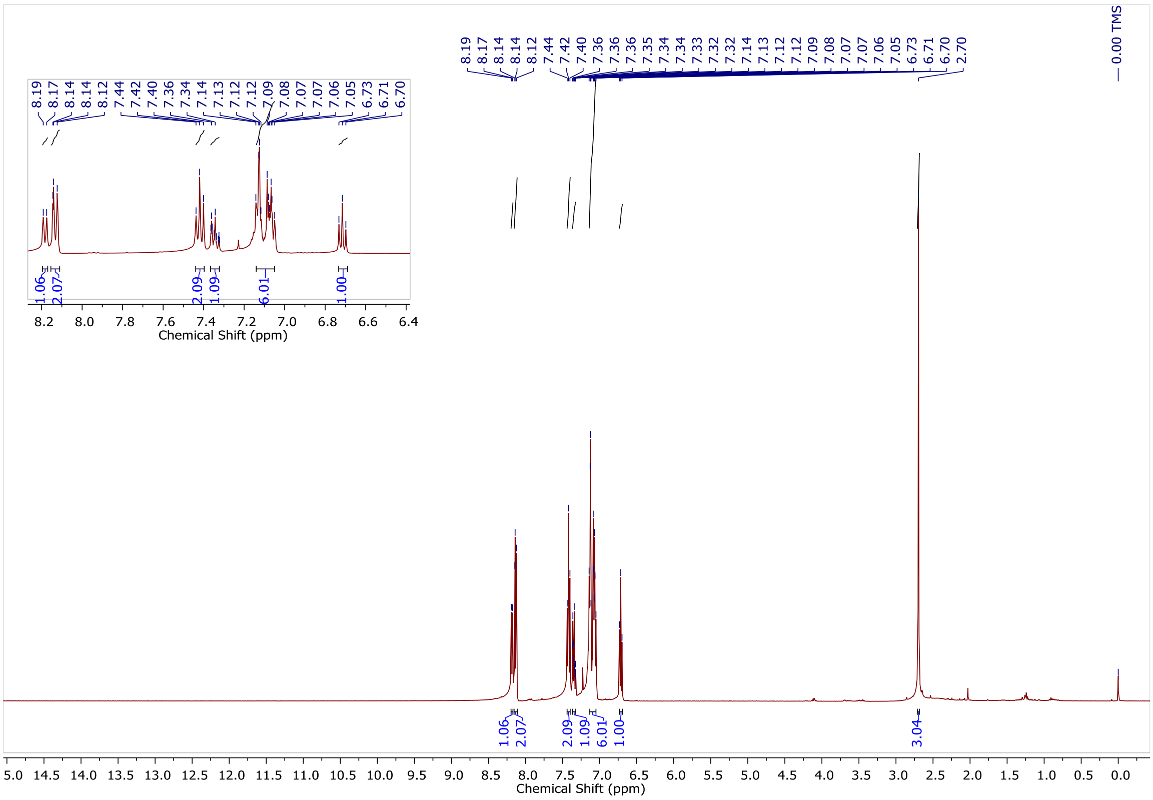

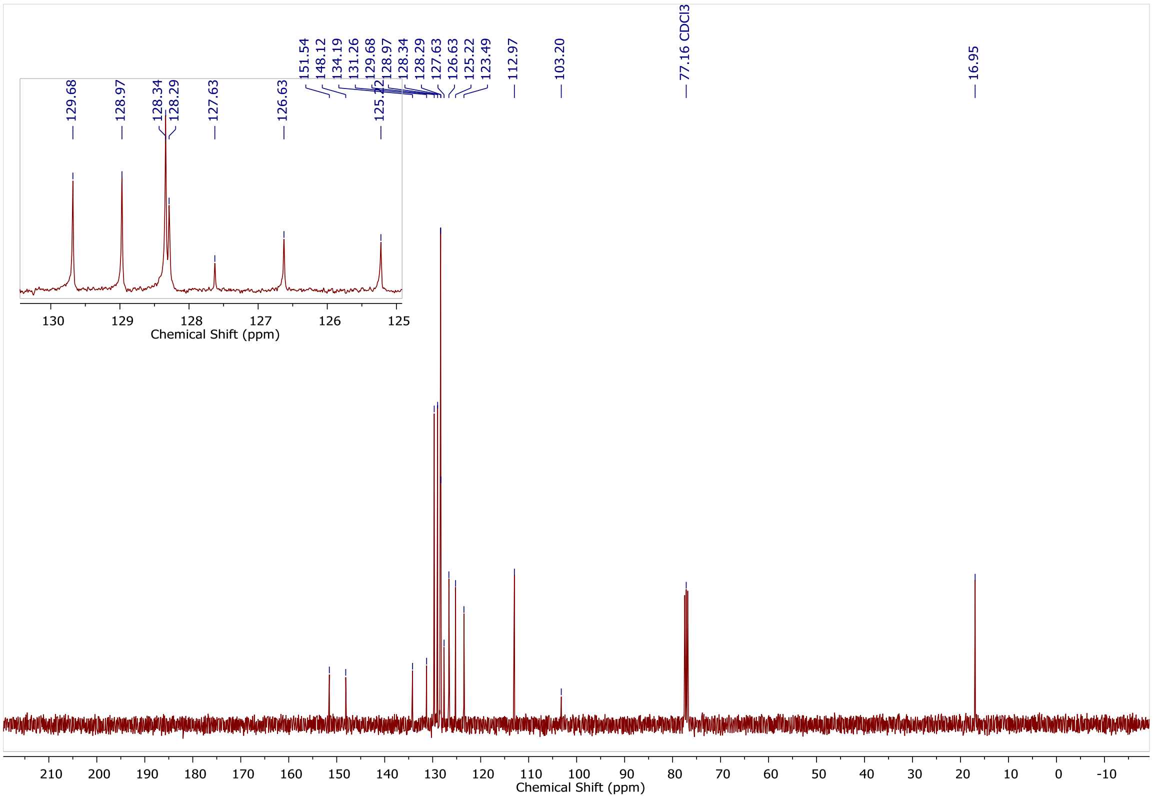

^{1}H NMR (top) ^{13}C NMR (bottom) $CDCl_3$ spectra of compound **4k**

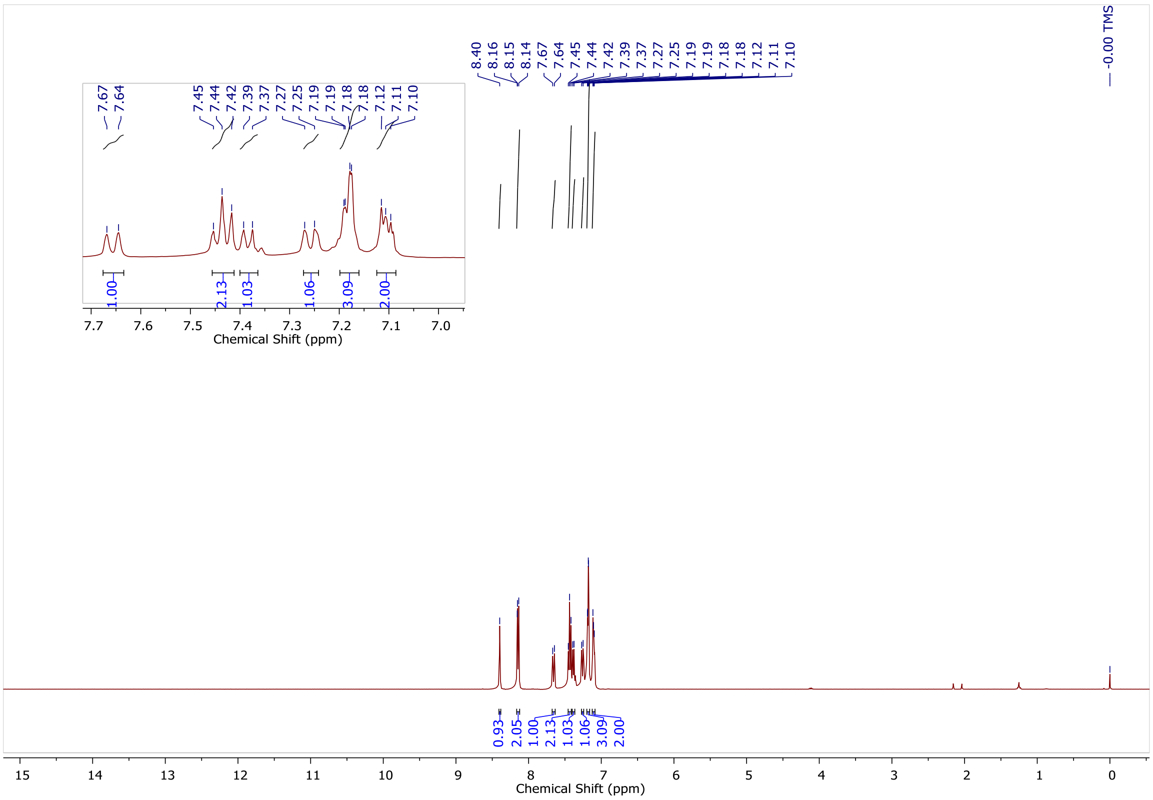

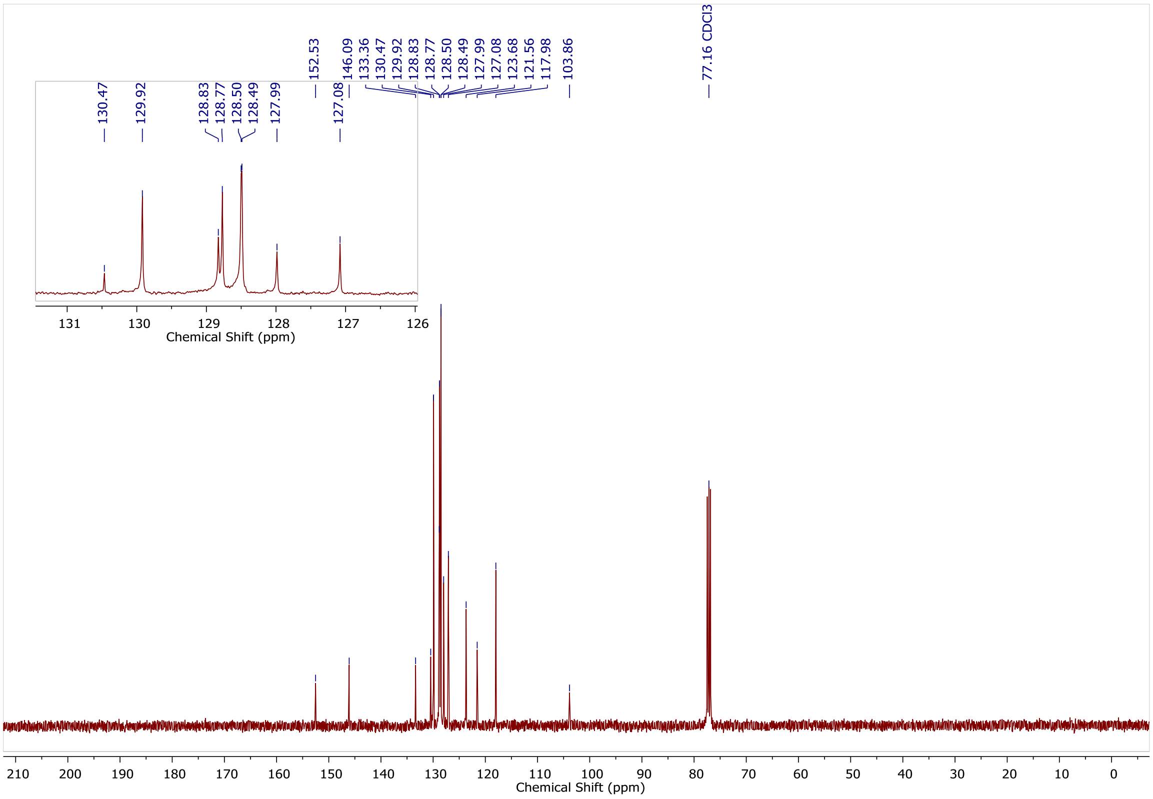

^{1}H NMR (top) ^{13}C NMR (bottom) $CDCl_3$ spectra of compound **4l**

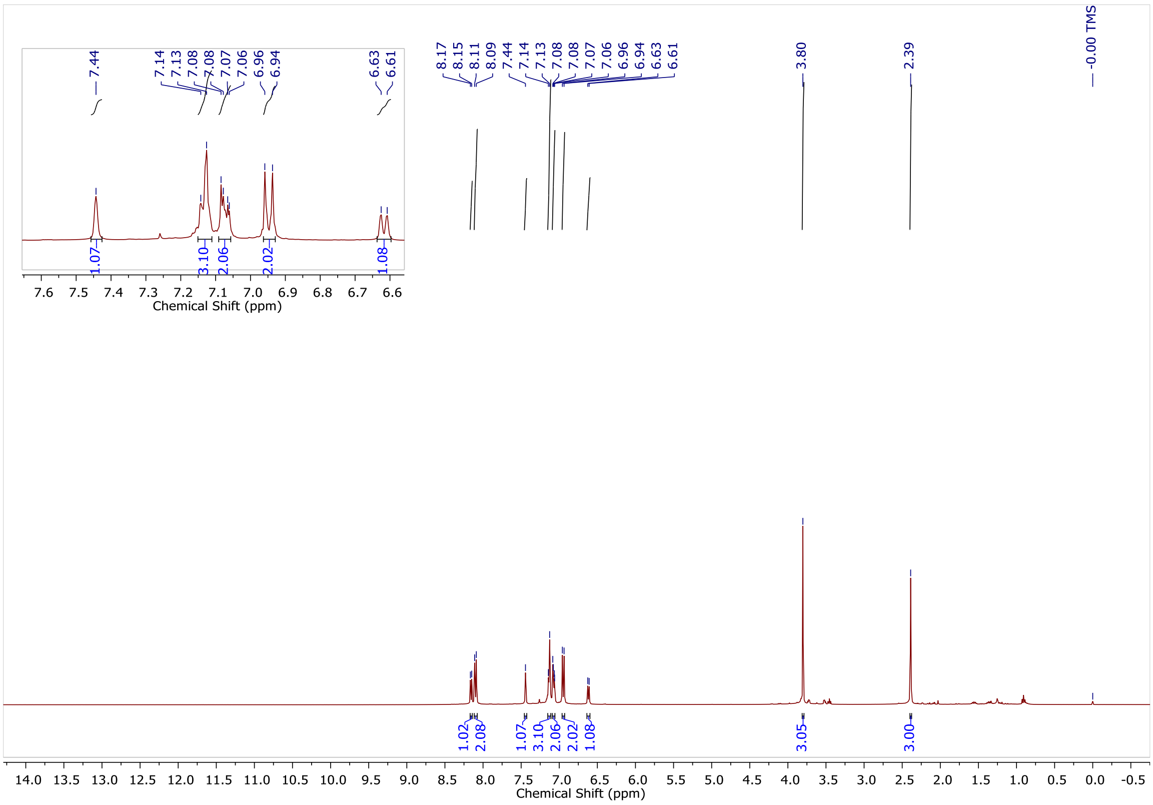

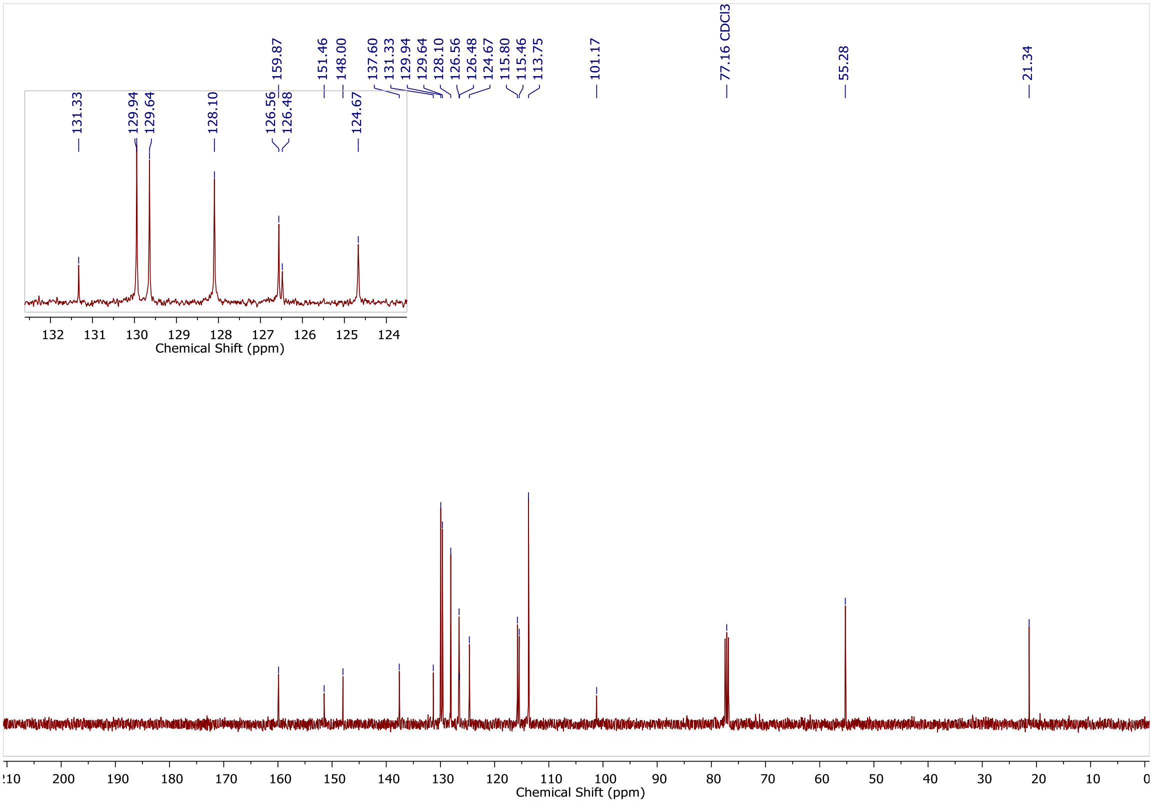

^{1}H NMR (top) ^{13}C NMR (bottom) $CDCl_3$ spectra of compound **4m**

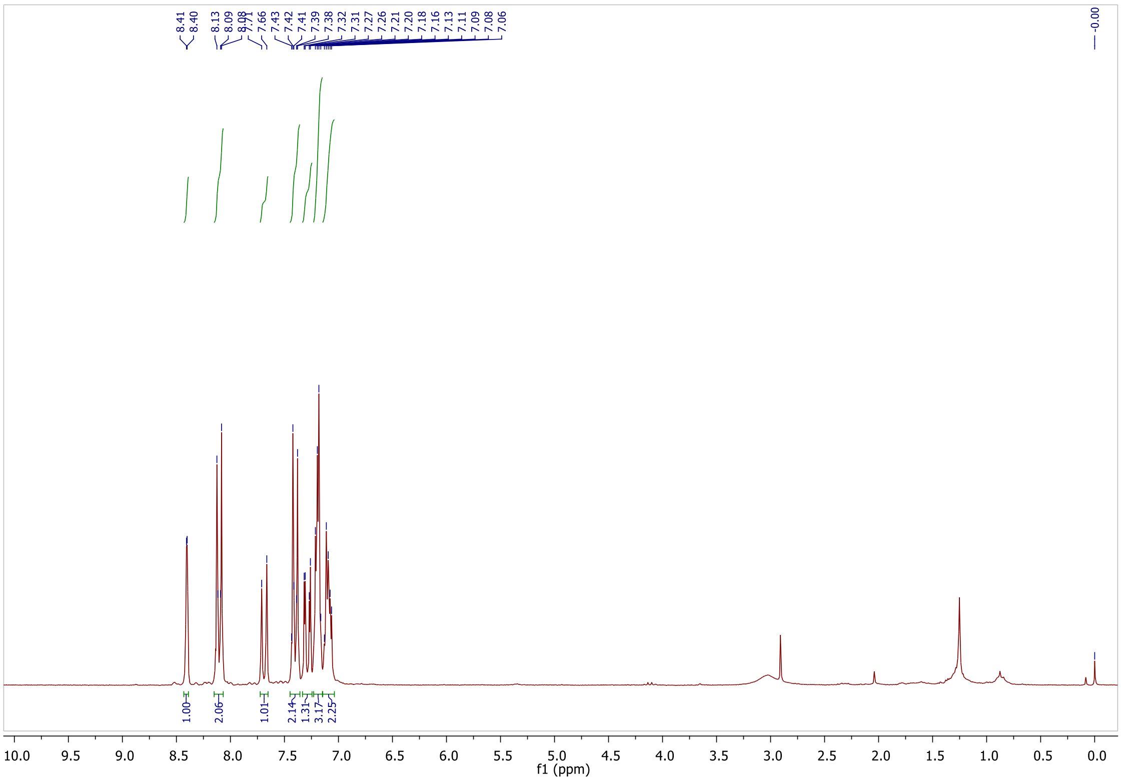

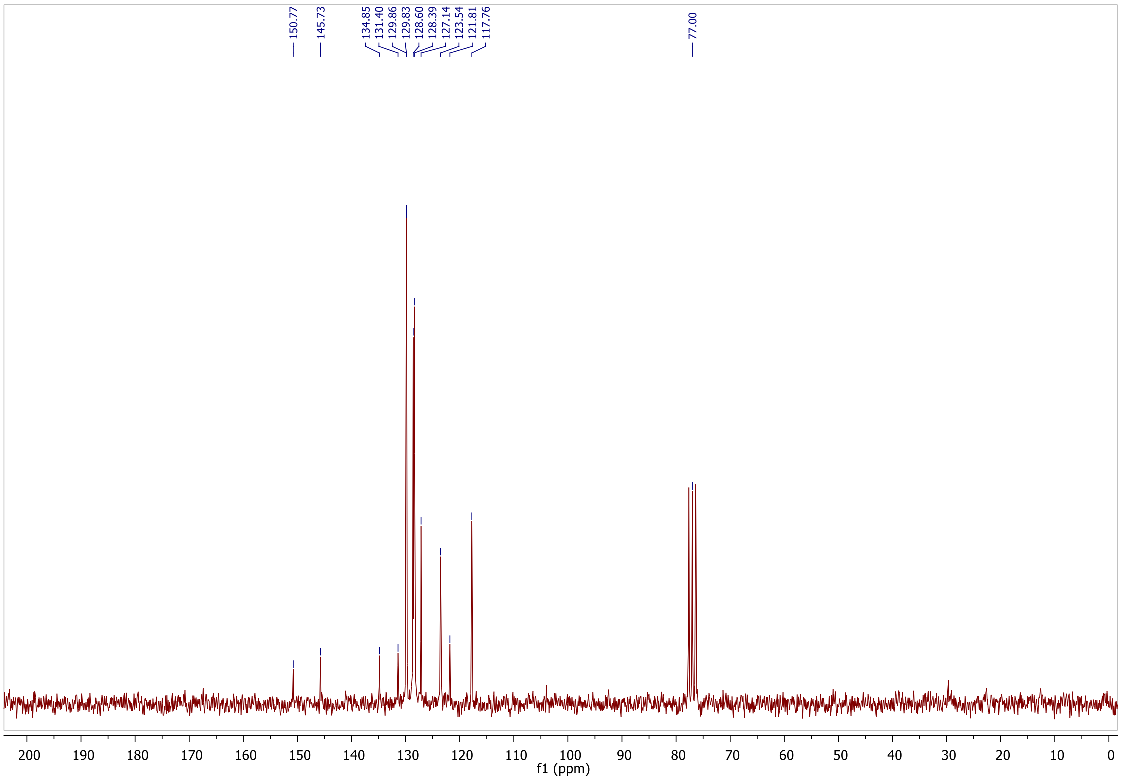

^{1}H NMR (top) ^{13}C NMR (bottom) $CDCl_3$ spectra of compound **4n**

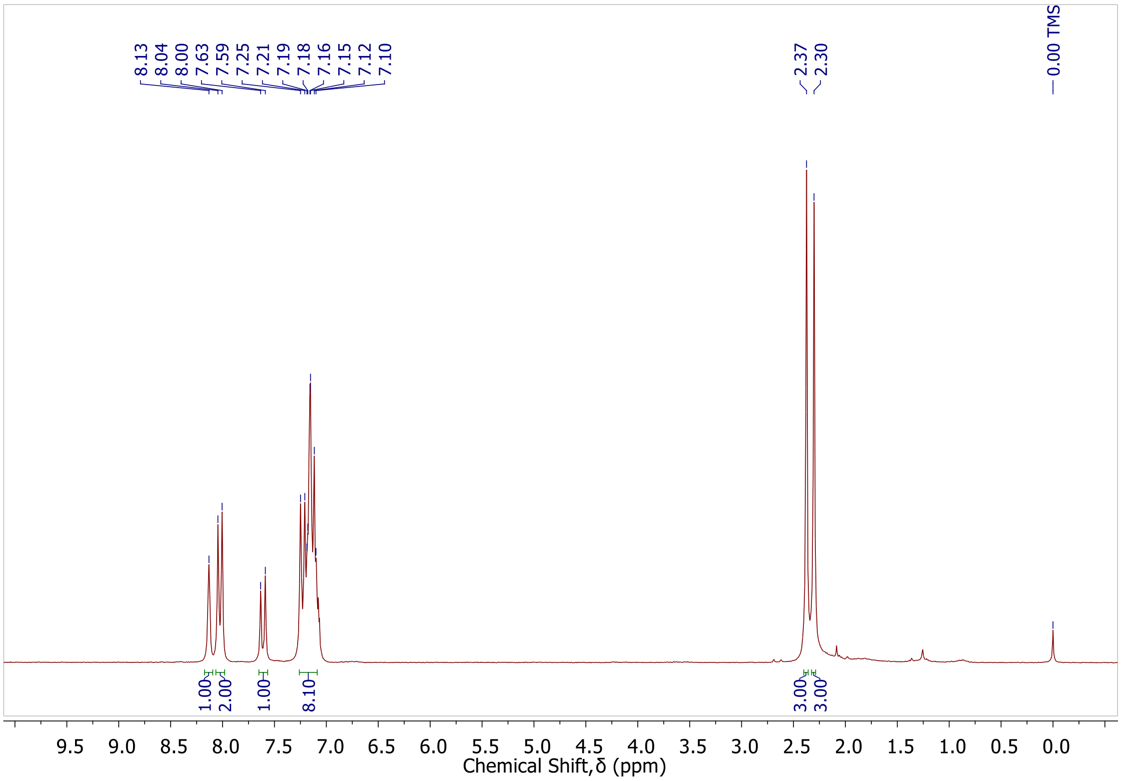

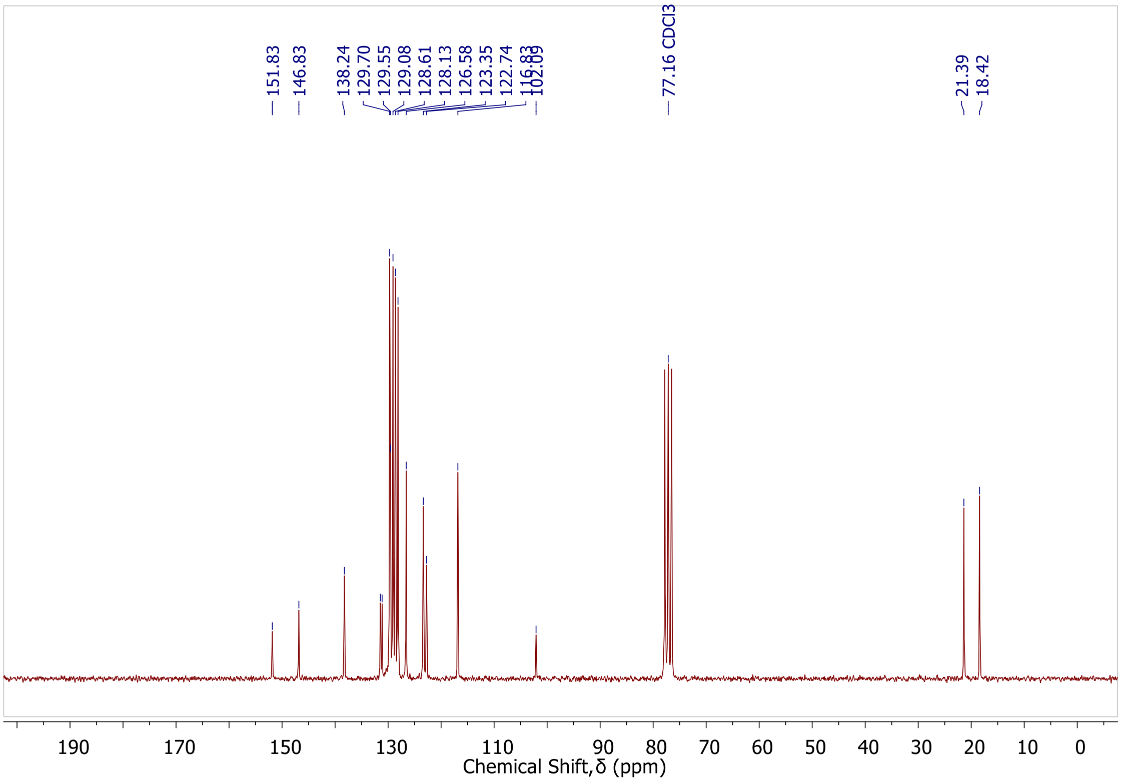

^{1}H NMR (top) ^{13}C NMR (bottom) $CDCl_3$ spectra of compound **4o**

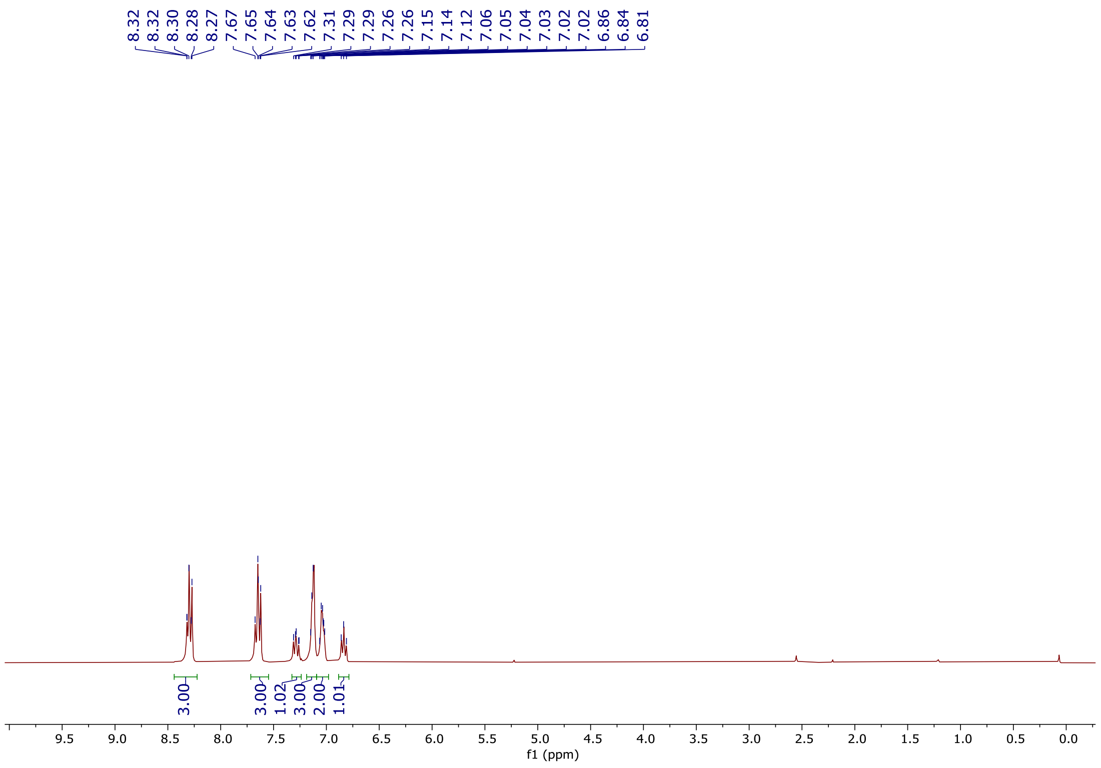

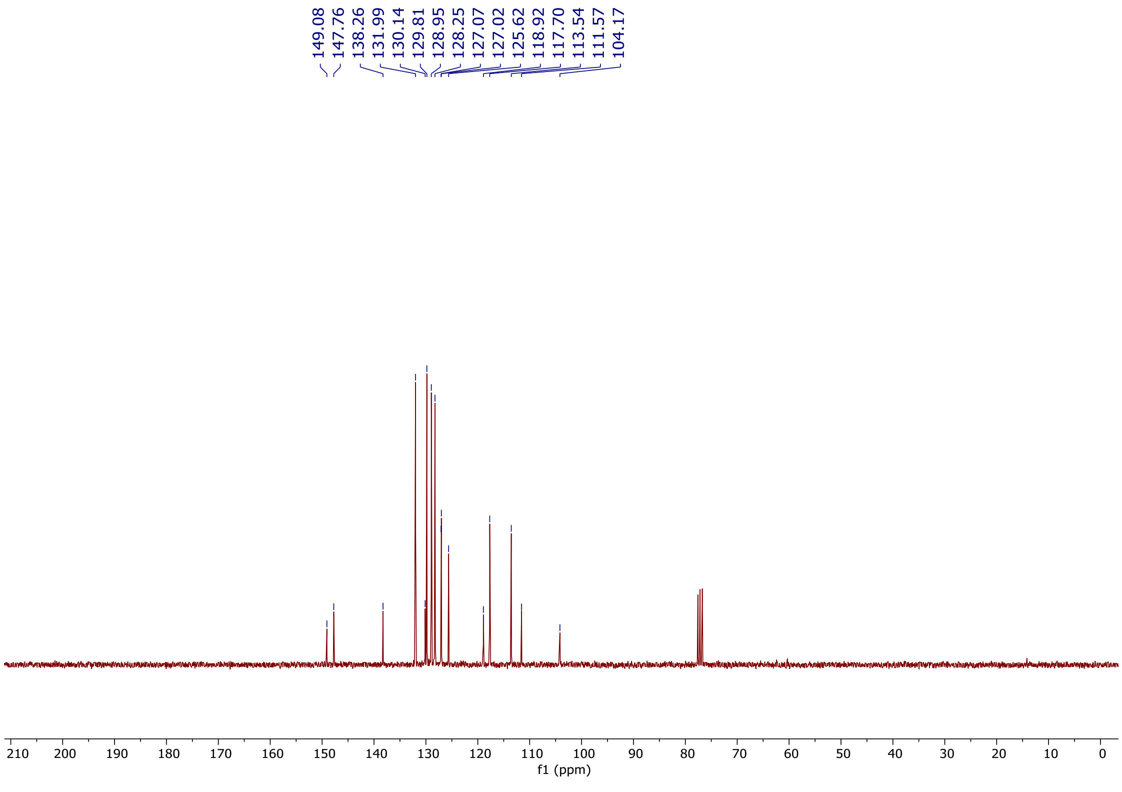

^{1}H NMR (top) ^{13}C NMR (bottom) $CDCl_3$ spectra of compound **4p**

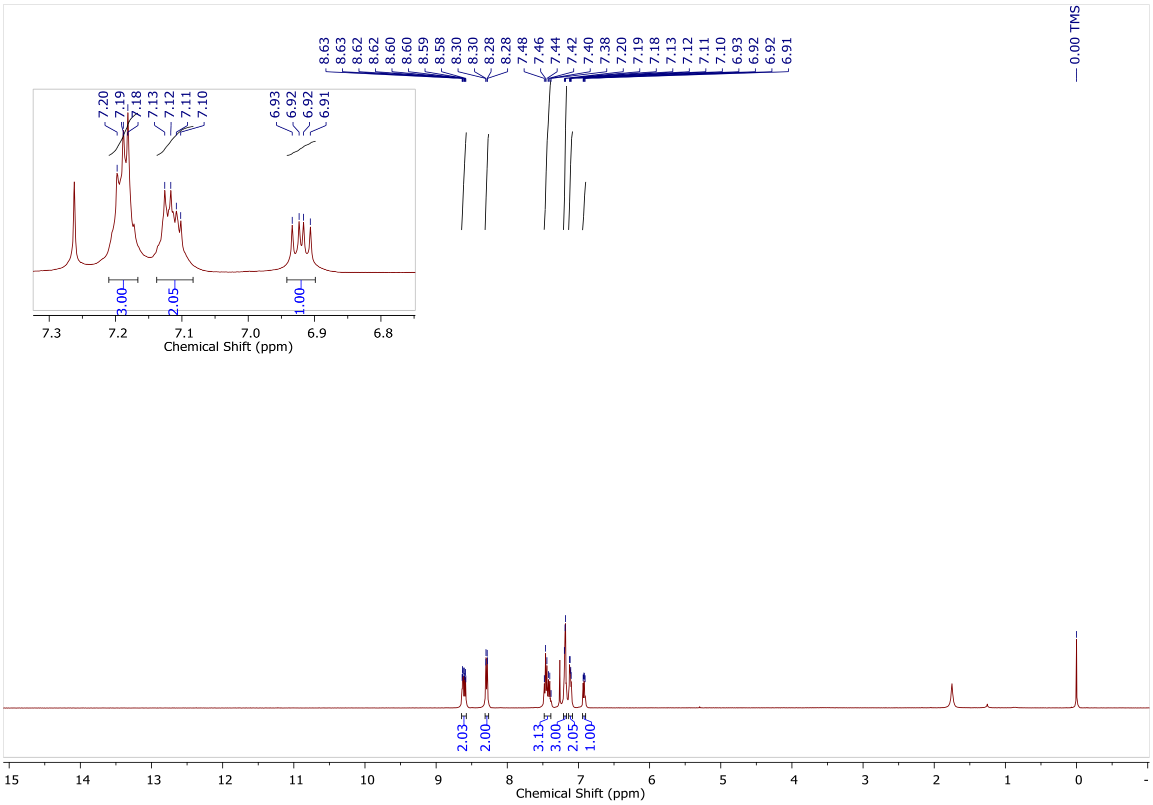

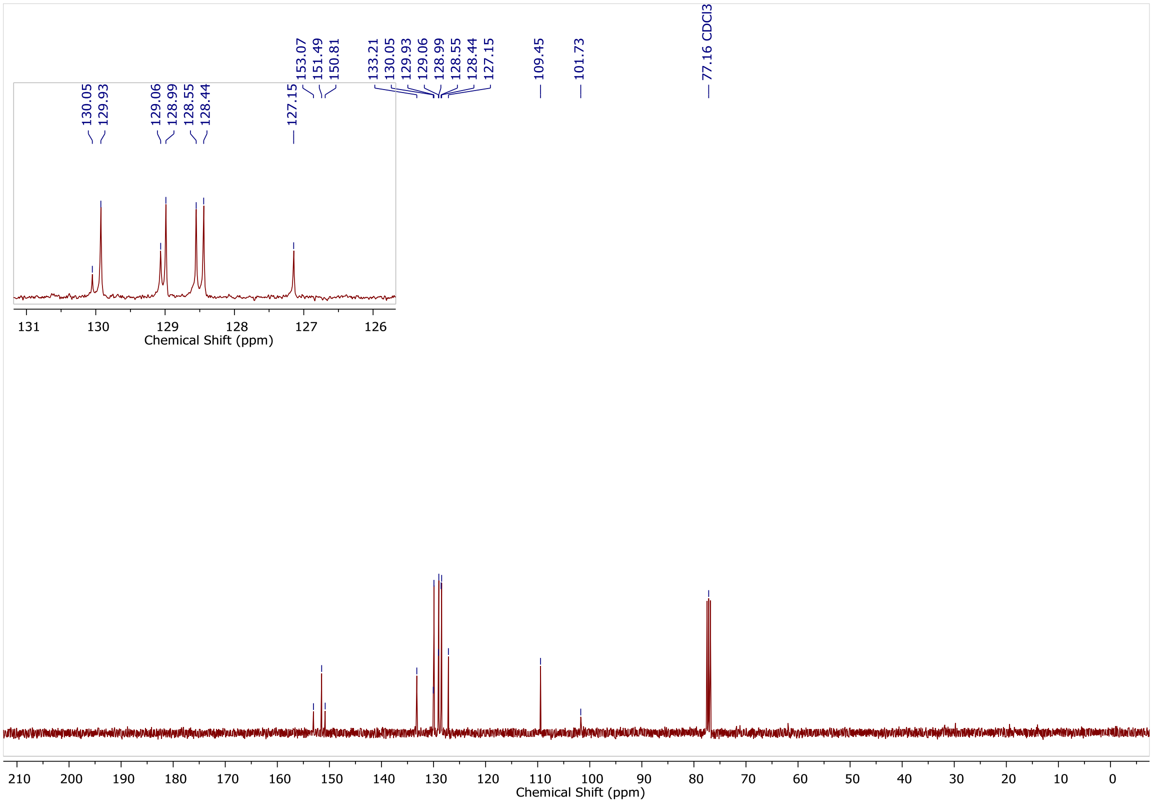

^{1}H NMR (top) ^{13}C NMR (bottom) $CDCl_3$ spectra of compound **6a**

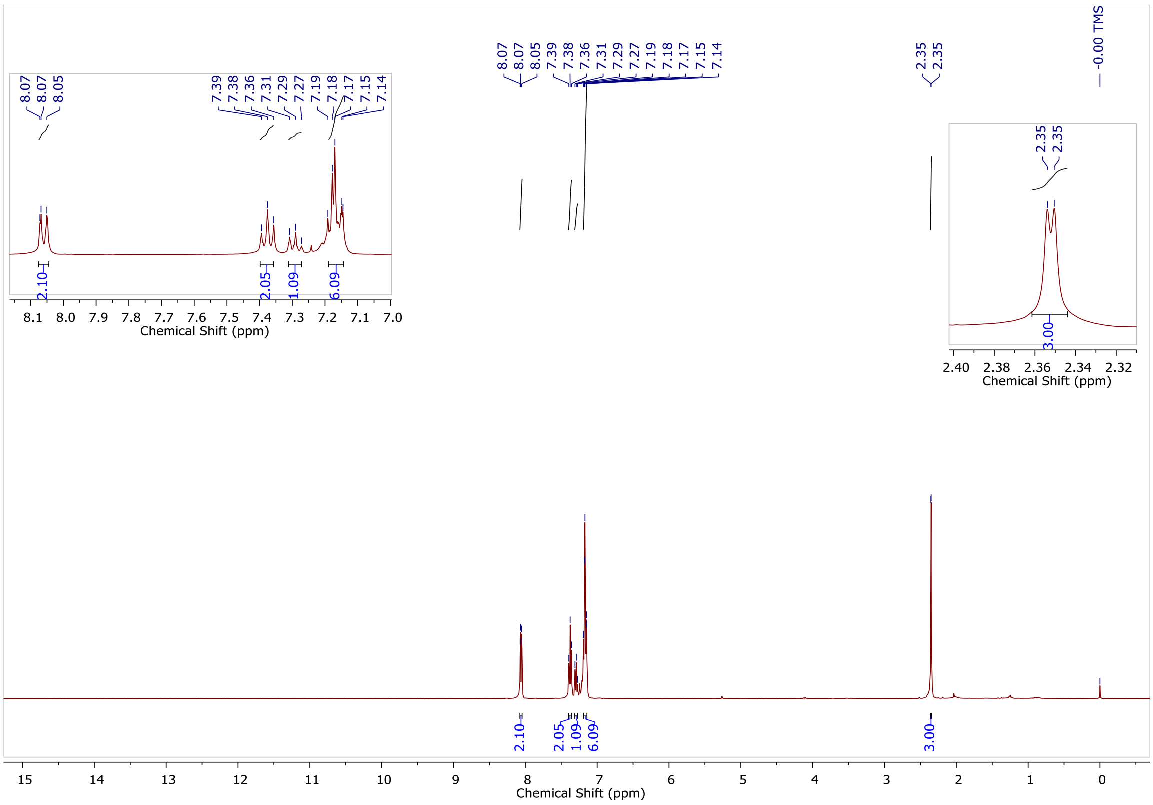

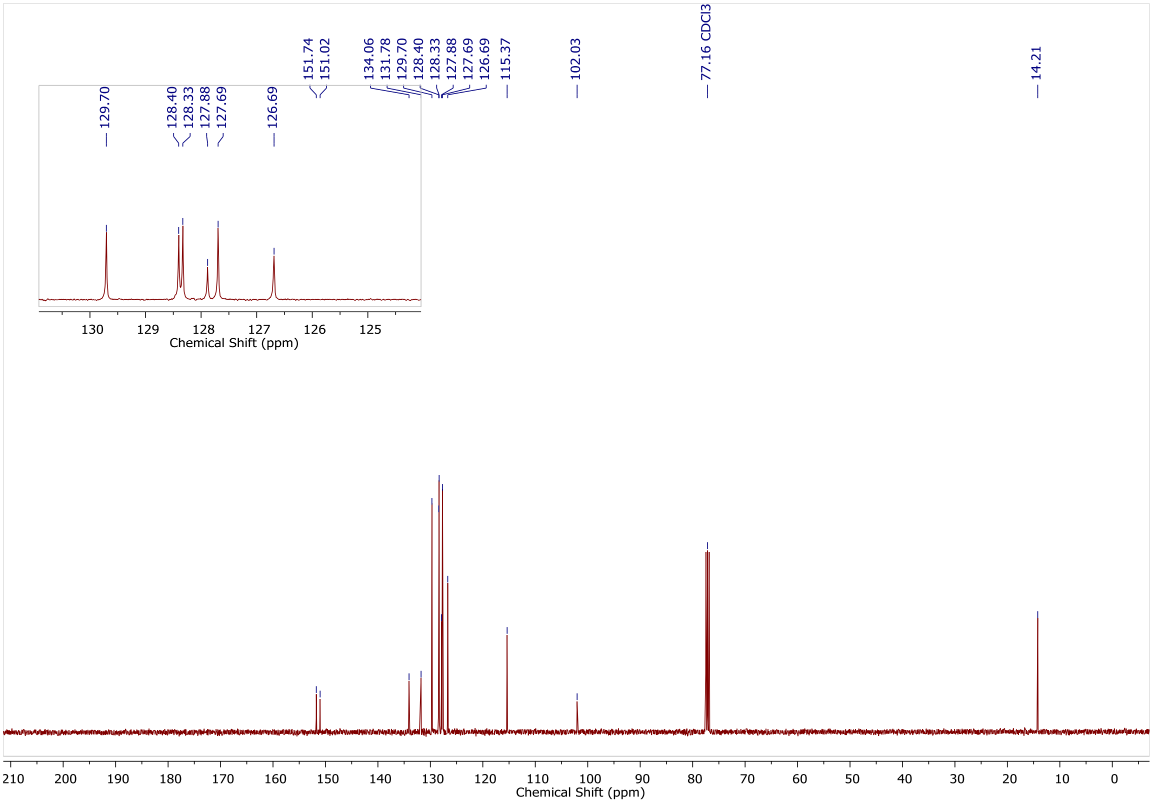

^{1}H NMR (top) ^{13}C NMR (bottom) $CDCl_3$ spectra of compound **6b**

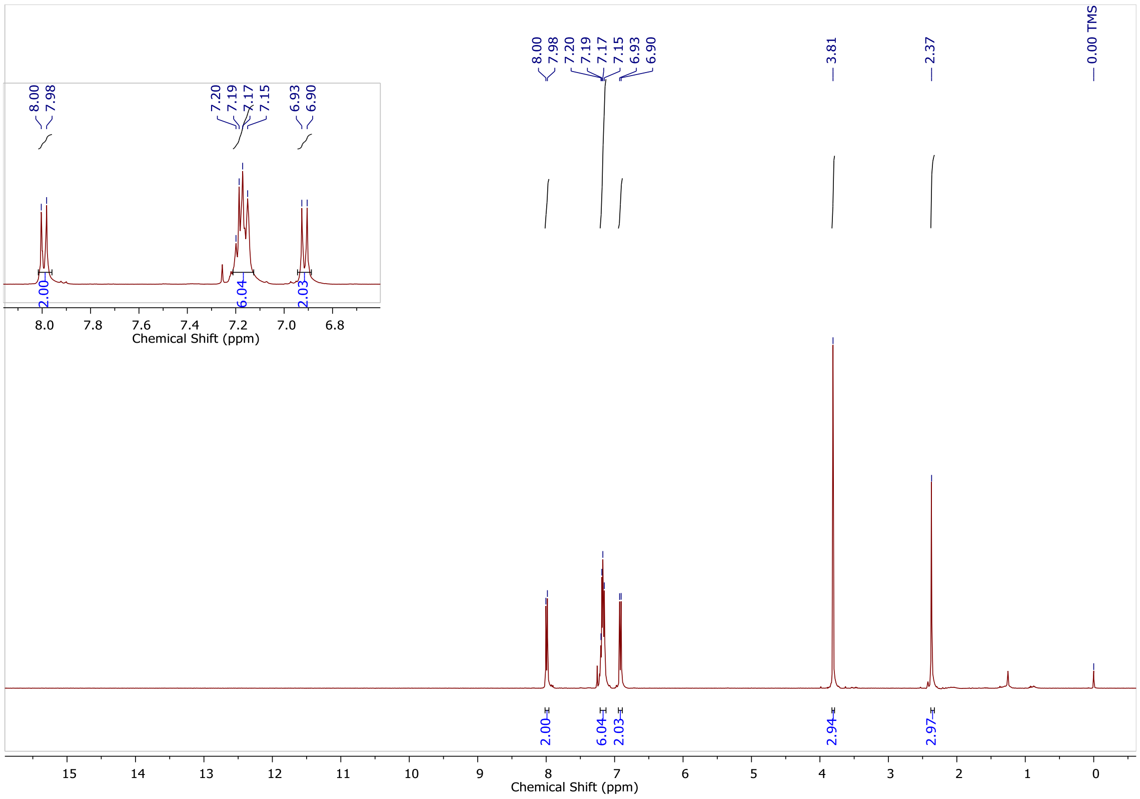

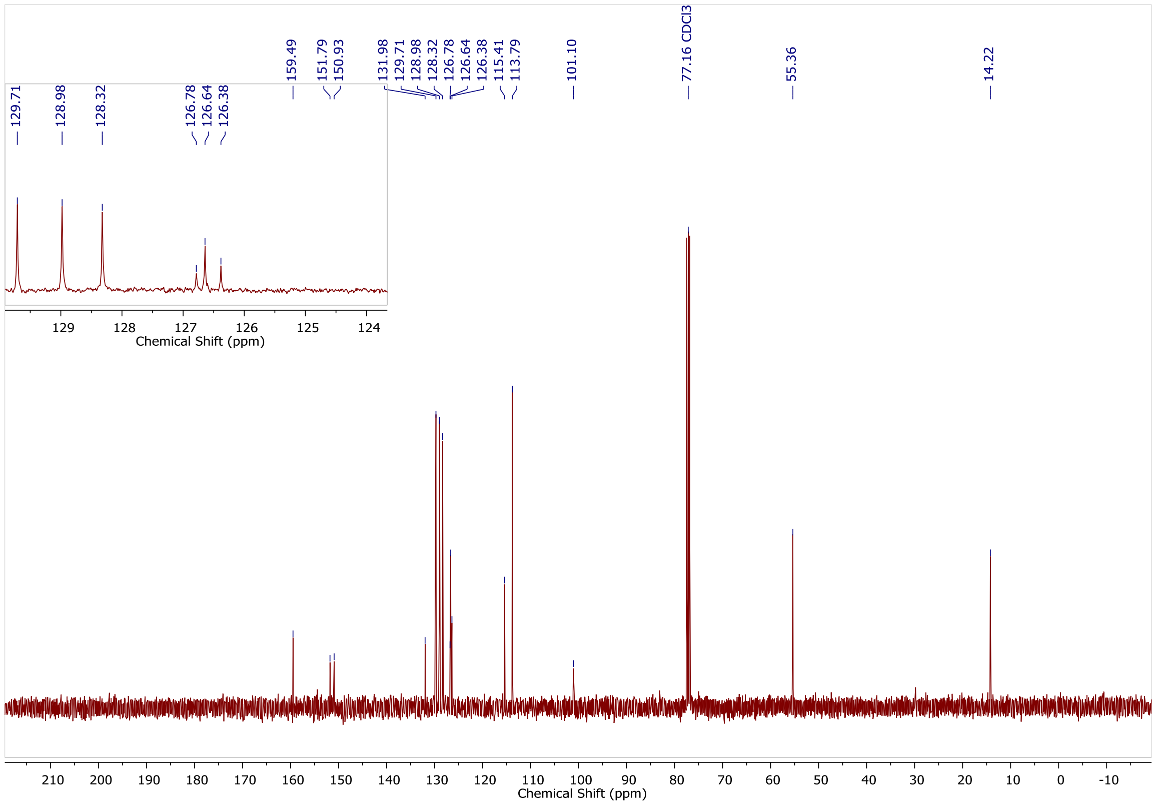

^{1}H NMR (top) ^{13}C NMR (bottom) $CDCl_3$ spectra of compound **6c**

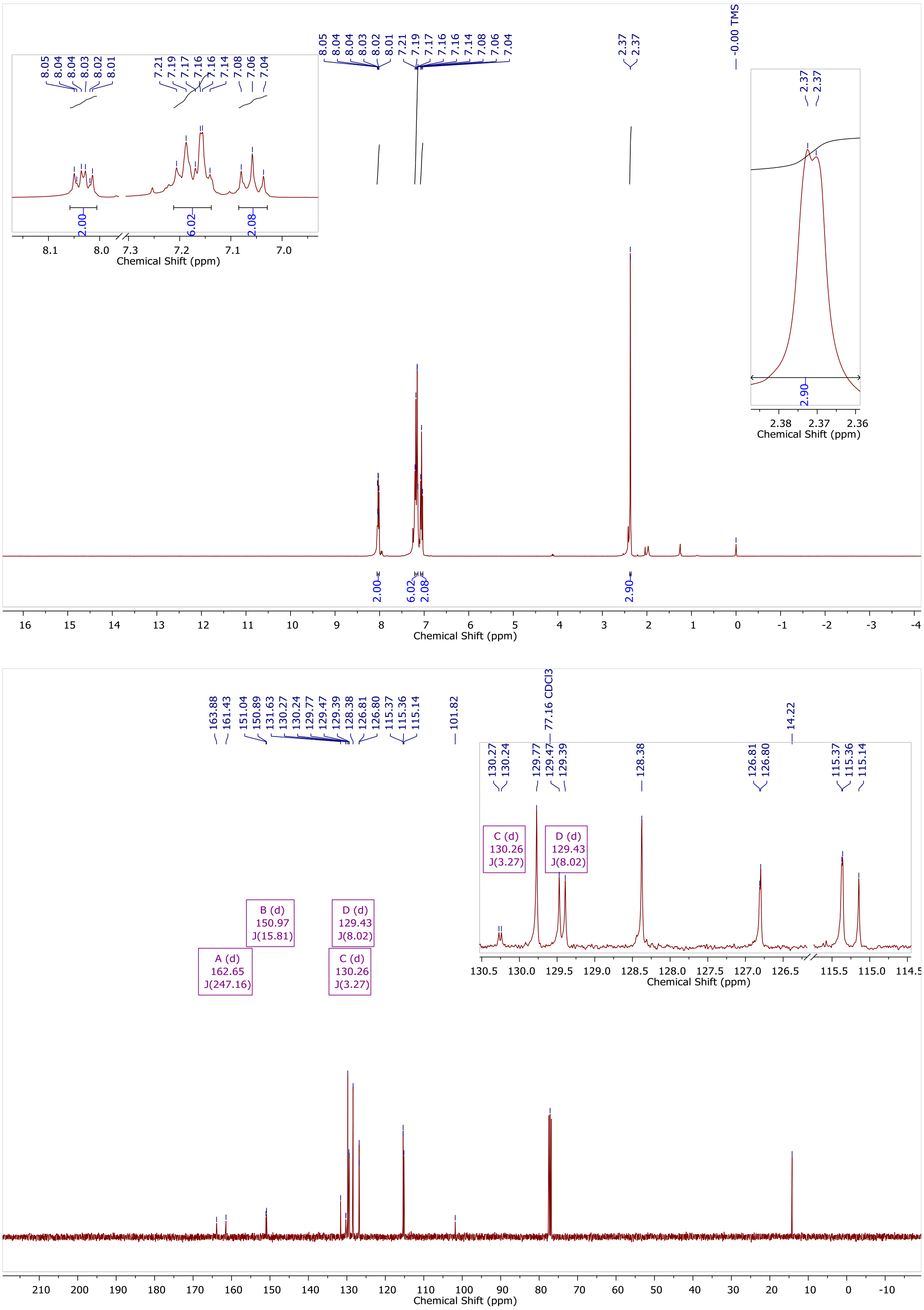

^{1}H NMR (top) ^{13}C NMR (bottom) $CDCl_3$ spectra of compound **6d**

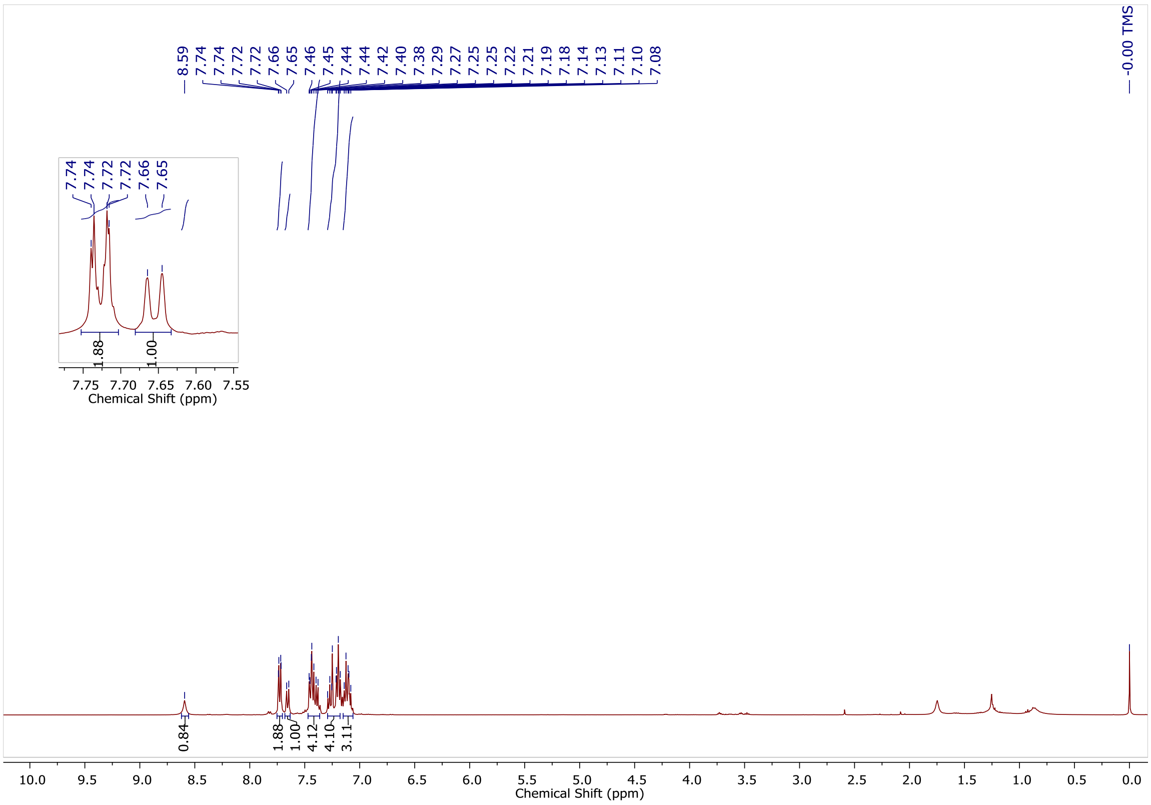

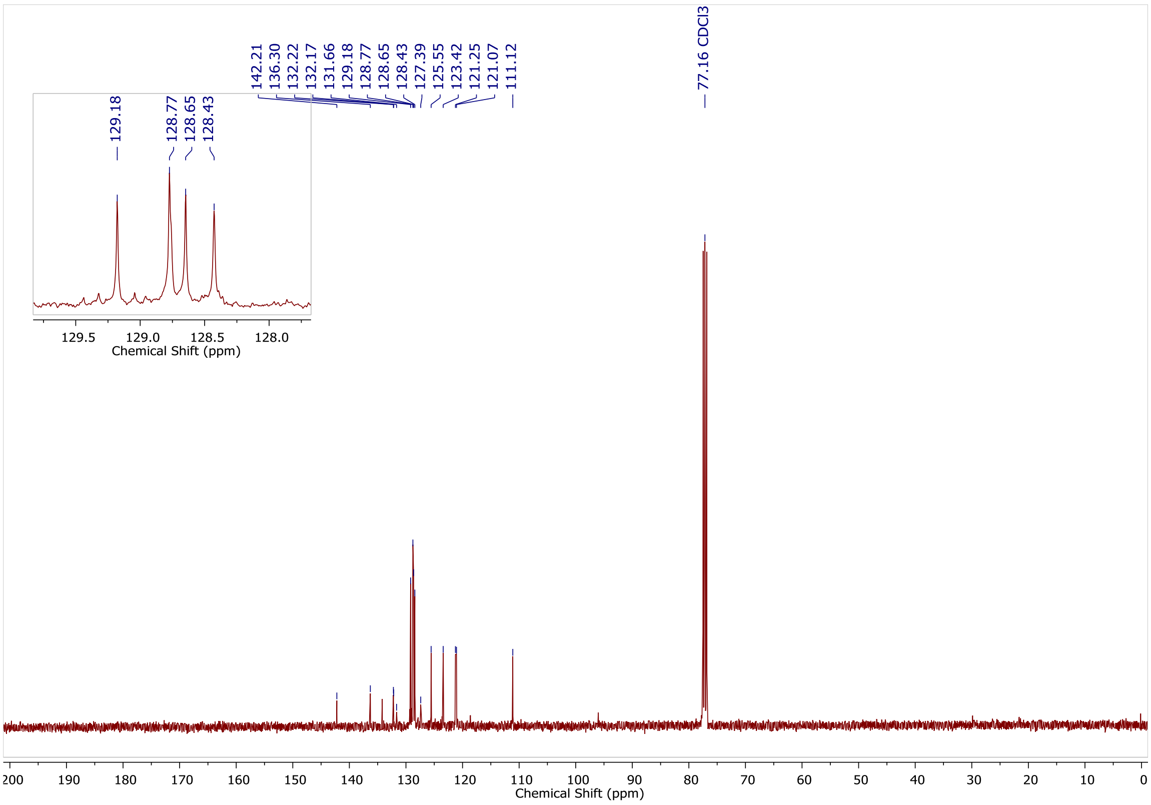

^{1}H NMR (top) ^{13}C NMR (bottom) $CDCl_3$ spectra of compound **6e**

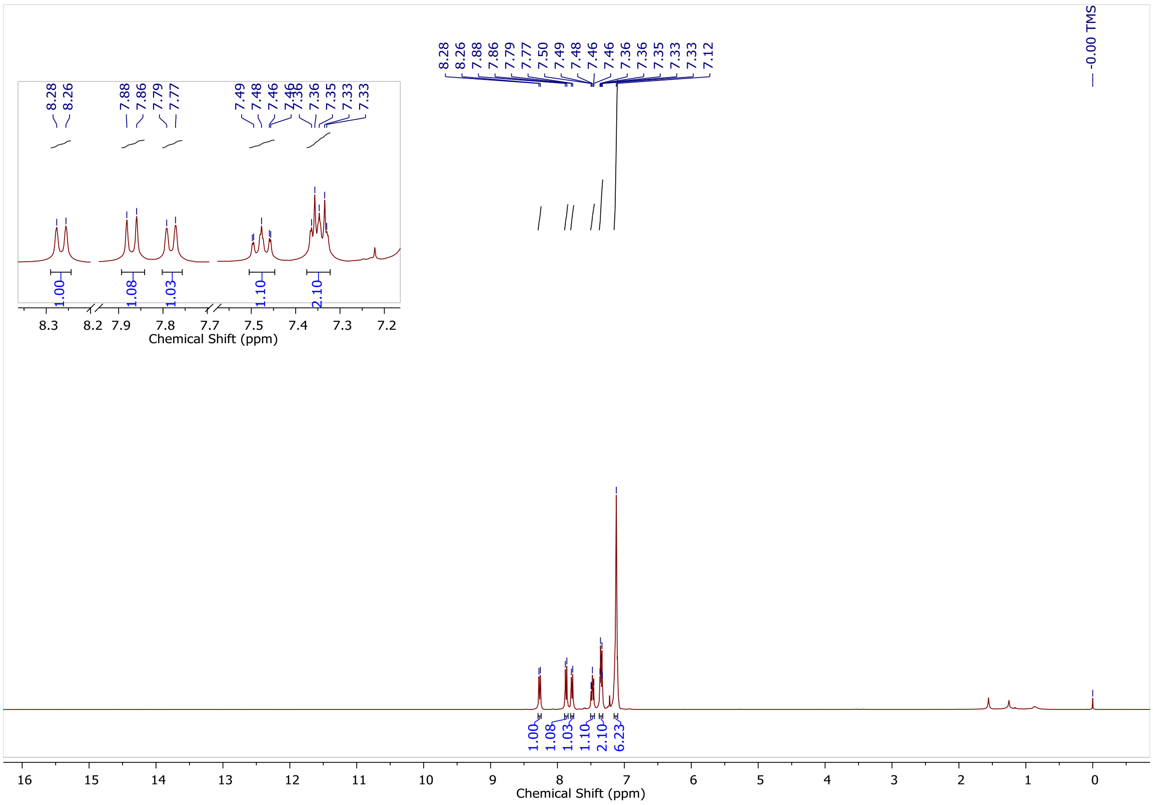

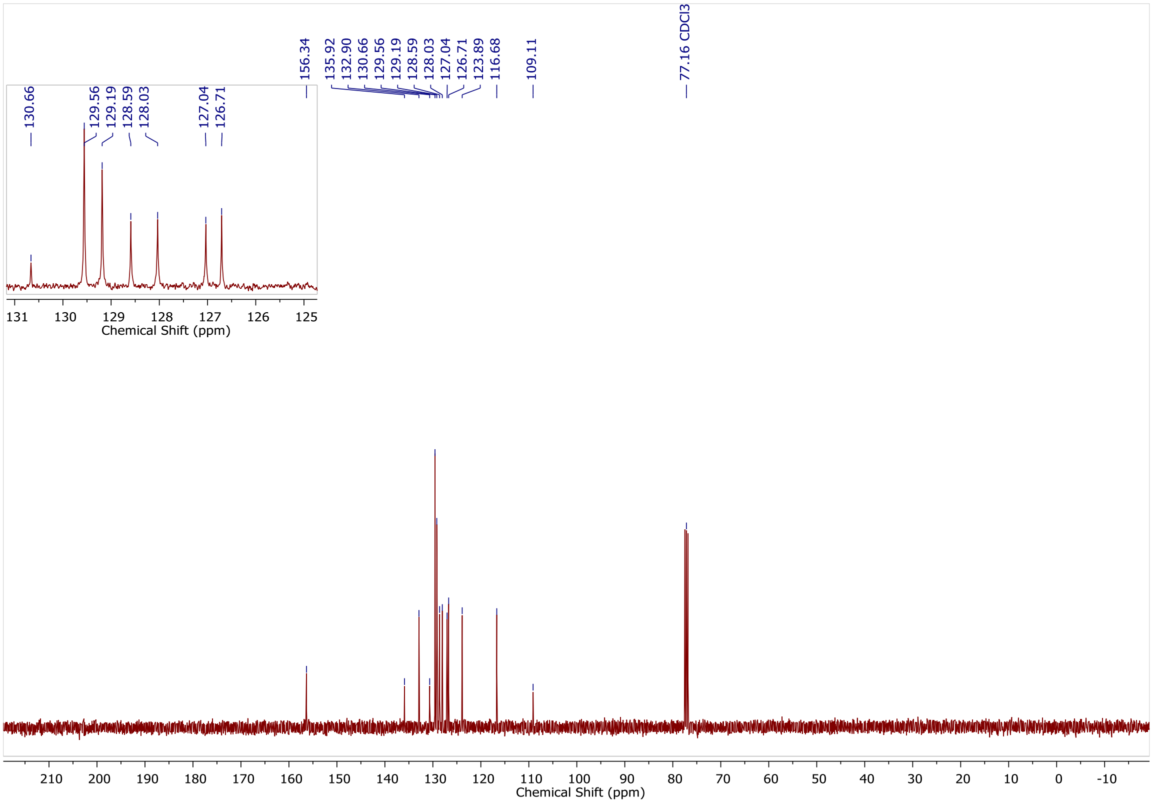

^{1}H NMR (top) ^{13}C NMR (bottom) $CDCl_3$ spectra of compound **6f**

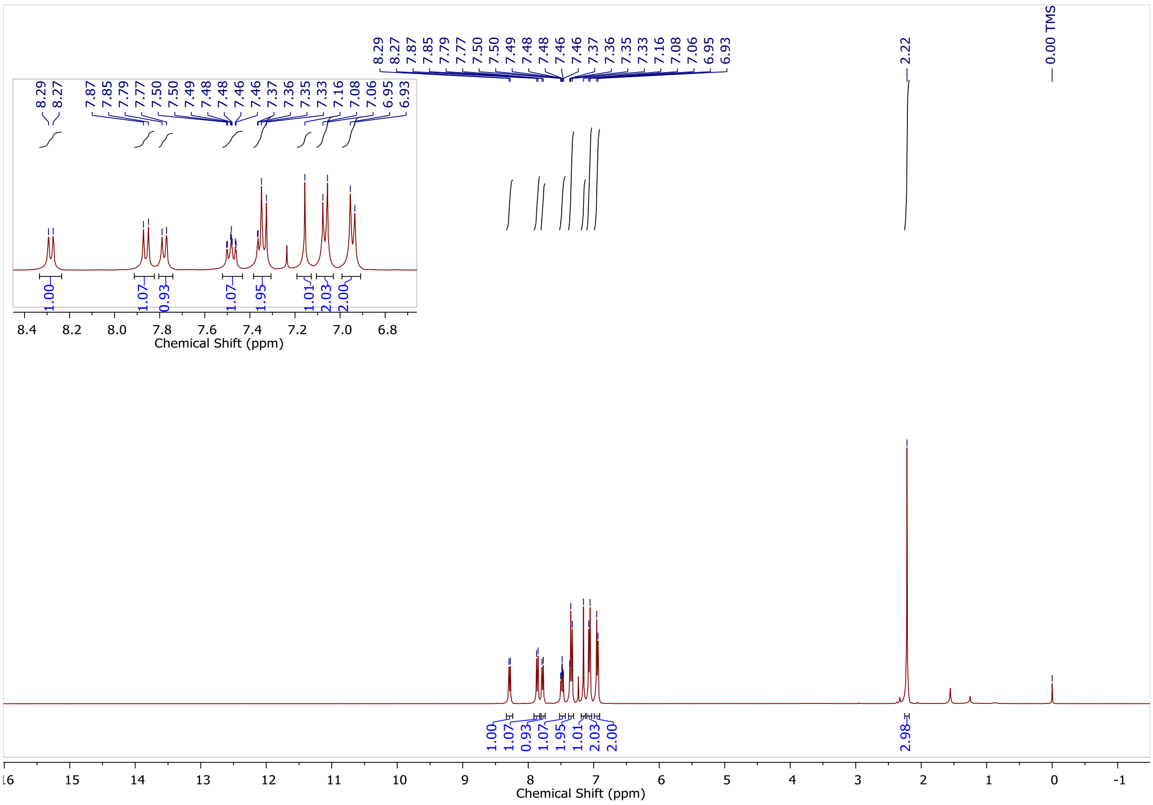

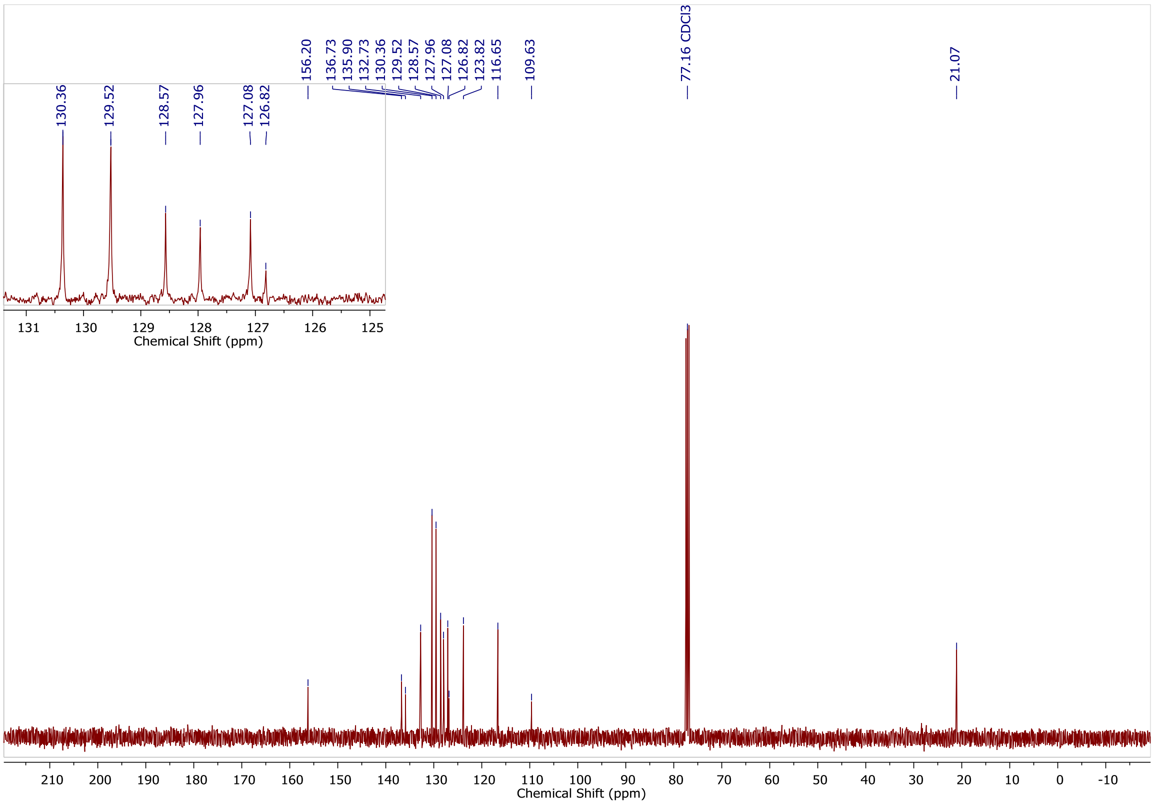

^{1}H NMR (top) ^{13}C NMR (bottom) $CDCl_3$ spectra of compound **6g**

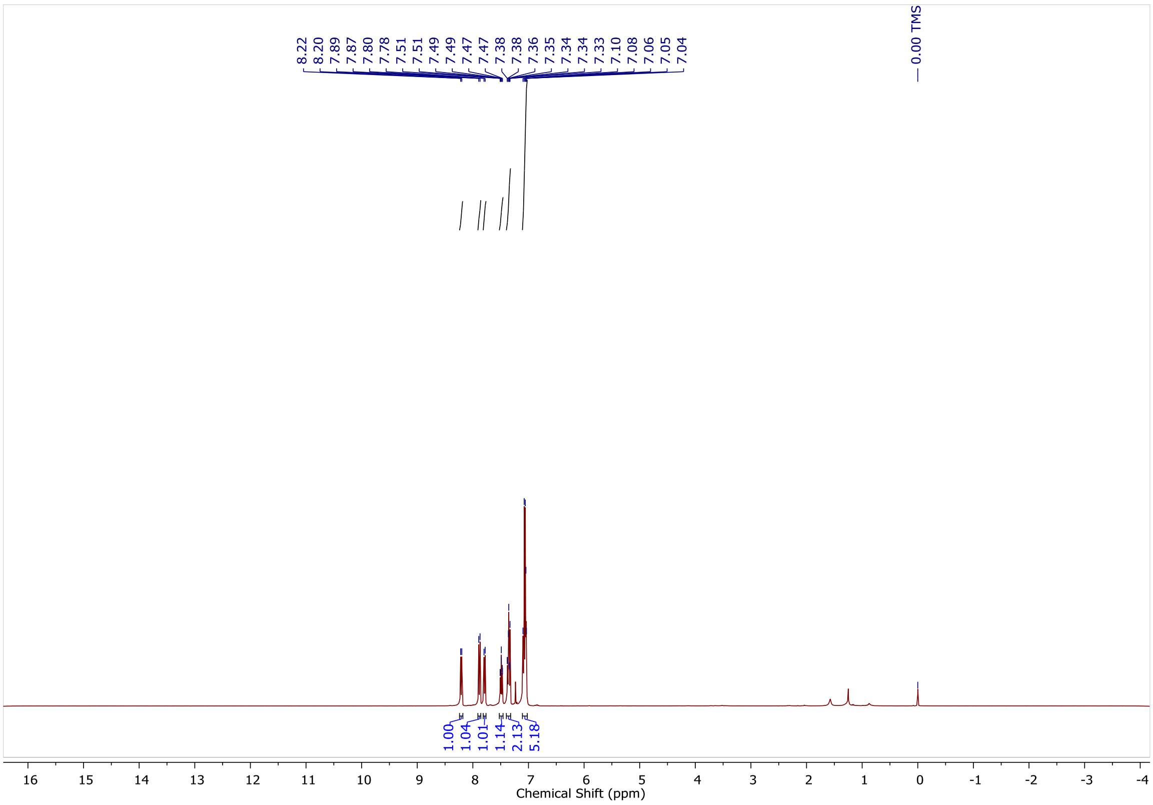

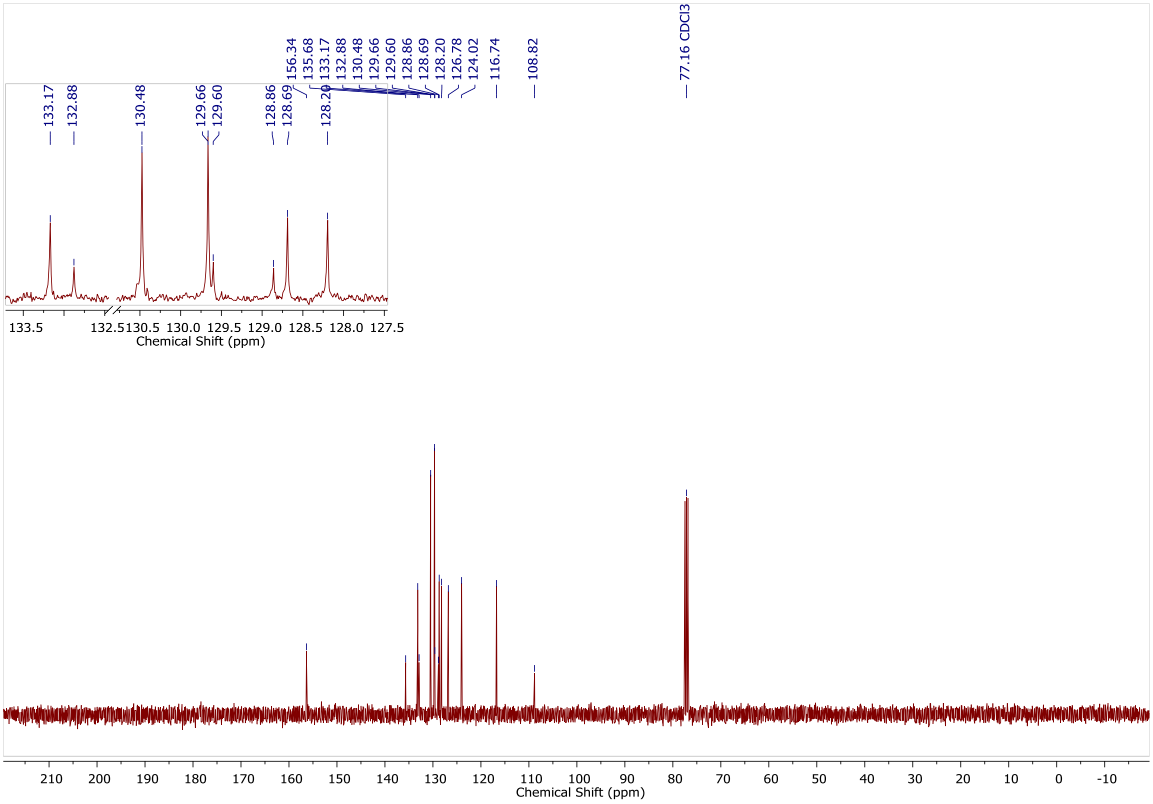

^{1}H NMR (top) ^{13}C NMR (bottom) $CDCl_3$ spectra of compound **6h**

References

[1] F.-J. Wang, H. Xu, M. Xin, Z. Zhang, *Mol. Divers.* **2016**, 20, 659–666.

[2] H. Huang, X. Ji, X. Tang, M. Zhang, X. Li, H. Jiang, *Org. Lett.* **2013**, 15, 6254–6257.

[3] J. L. Bescont, C. B.-Patient, S. Piguel, *Eur. J. Org. Chem.* **2020**, 2101–2109.

[4] S. K. Samanta, M. K. Bera, *Org. Biomol. Chem.* **2019,** 17, 6441–6449.

[5] N. Gunaganti, A. Kharbanda, N. R. Lakkaniga, L. Zhang, R. Cooper, H.-y. Li, B. Frett, *Chem. Commun.* **2018**, 54, 12954–12957.

[6] Q. Li, M. Zhou, L. Han, Q. Cao, X. Wang, L. Zhao, J. Zhou, H. Zhang, *Chem. Biol. Drug. Des.* **2015**, 86, 849– 856.

[7] A. J. Stasyuk, M. Banasiewicz, M. K. Cyranski, D. T. Gryko, *J. Org. Chem.* **2012**, 77, 5552−5558.

[8] M. V. P. S. Vishnuvardhan, I. B. Sayeed, V. L. Nayak, M. A. Shareef, A. Kamal, *J. Chem. Pharm. Res.* **2017**, 9, 210–219.

[9] R. Moszczynski-Petkowski, J. Majer, M. Borkowska, L. Bojarski, S. Janowska, M. Matloka, F. Stefaniak, D. Smuga, K. Bazydlo, K. Dubiel, M. Wieczorek, *Eur. J. Med. Chem.* **2018**, 155, 96–116.

[10] A. Kamal, G. B. Kumar, V. L. Nayak, V. S. Reddy, A. B. Shaik, Rajender, M. K. Reddy, *Med. Chem. Commun.* **2015**, 6, 606–612.

[11] T. Pyl, R. Giebelmann, H. Beyer, *Liebigs Ann. Chem.* **1961**, 643, 145–153.

[12] A. Kamal, D. Dastagiri, M. J. Ramaiah, J. S. Reddy, E. V. Bharathi, C. Srinivas, S. N. C. V. L. Pushpavalli, D. Pal, M. P.-Bhadra, *Chem. Med. Chem.* **2010**, 5, 1937–1947.
